# A genetically targeted reporter for PET imaging of deep neuronal circuits in mammalian brains

**DOI:** 10.15252/embj.2021107757

**Published:** 2021-10-12

**Authors:** Masafumi Shimojo, Maiko Ono, Hiroyuki Takuwa, Koki Mimura, Yuji Nagai, Masayuki Fujinaga, Tatsuya Kikuchi, Maki Okada, Chie Seki, Masaki Tokunaga, Jun Maeda, Yuhei Takado, Manami Takahashi, Takeharu Minamihisamatsu, Ming‐Rong Zhang, Yutaka Tomita, Norihiro Suzuki, Anton Maximov, Tetsuya Suhara, Takafumi Minamimoto, Naruhiko Sahara, Makoto Higuchi

**Affiliations:** ^1^ Department of Functional Brain Imaging National Institutes for Quantum and Radiological Science and Technology Chiba Japan; ^2^ Department of Radiopharmaceuticals Development National Institutes for Quantum and Radiological Science and Technology Chiba Japan; ^3^ Department of Neurology Keio University School of Medicine Tokyo Japan; ^4^ Department of Neuroscience The Scripps Research Institute La Jolla CA USA

**Keywords:** fluorescence, neural circuit, non‐invasive, PET, reporter imaging, Neuroscience

## Abstract

Positron emission tomography (PET) allows biomolecular tracking but PET monitoring of brain networks has been hampered by a lack of suitable reporters. Here, we take advantage of bacterial dihydrofolate reductase, ecDHFR, and its unique antagonist, TMP, to facilitate *in vivo* imaging in the brain. Peripheral administration of radiofluorinated and fluorescent TMP analogs enabled PET and intravital microscopy, respectively, of neuronal ecDHFR expression in mice. This technique can be used to the visualize neuronal circuit activity elicited by chemogenetic manipulation in the mouse hippocampus. Notably, ecDHFR‐PET allows mapping of neuronal projections in non‐human primate brains, demonstrating the applicability of ecDHFR‐based tracking technologies for network monitoring. Finally, we demonstrate the utility of TMP analogs for PET studies of turnover and self‐assembly of proteins tagged with ecDHFR mutants. These results establish opportunities for a broad spectrum of previously unattainable PET analyses of mammalian brain circuits at the molecular level.

## Graphical abstract

Application of bacterial dihydrofolate reductase ecDHFR and its unique antagonist TMP achieves a broad spectrum of previously unattainable *in vivo* PET analyses of mammalian brain circuits at the molecular level.

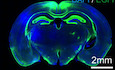

## Introduction

Contemporary methods for live imaging of genetically encoded reporters have transformed basic and translational neuroscience (Huang & Zeng, 2013; Lin & Schnitzer, 2016). Many excellent fluorescence‐based tools have recently been introduced for the visualization of neuronal ensembles and individual synapses and for understanding the roles of neuronal cells in circuit development, function, and dysfunction (Hayashi‐Takagi *et al*, 2015; Iwano *et al*, 2018; Busche *et al*, 2019). Despite its tremendous utility, fluorescence‐based imaging remains largely unsuitable for simultaneous non‐invasive monitoring of reporters in deep brain regions or across the entire brain because of light scattering and relatively narrow fields of view. One promising strategy for solving this technical bottleneck is to complement optical microscopy with positron emission tomography (PET). While PET itself has a limited spatiotemporal resolution, a combination of the two techniques may offer comprehensive insights into the dynamics of molecules of interest on scales ranging from subcellular to global levels (Piel *et al*, 2014; Shimojo *et al*, 2015b). The possibility for such a bimodal analysis of xenografted cells has been demonstrated in peripheral tissues with a virally driven triple reporter comprised of a fluorescent protein, luciferase, and thymidine kinase derived from herpes simplex virus 1 (HSV‐TK) (Ray *et al*, 2007; Sun *et al*, 2009). However, no reports currently exist regarding bimodal imaging of the central nervous system with an intact blood‐brain barrier (BBB).

A major challenge for such a bimodal analysis of exogenous markers genetically targeted in the brain is the development of selective radioactive ligands that efficiently penetrate the BBB (Massoud *et al*, 2008). Indeed, even for the best‐characterized PET reporter, herpes simplex virus 1 (HSV‐TK), no BBB‐permeable compounds have been identified to date (Yaghoubi & Gambhir, 2006; Yaghoubi *et al*, 2012). Several groups have attempted PET with mutant forms of dopamine and cannabinoid receptors, but these methods have suffered from poor signal‐to‐noise ratio due to ligand interaction with endogenous proteins (Vandeputte *et al*, 2011; Yoon *et al*, 2014). A recent study reported brain PET imaging with an isoform of pyruvate kinase, PKM2, and its associated radiotracer [^18^F]DASA‐23 (Haywood *et al*, 2019). Our laboratory has also demonstrated that a designer receptor exclusively activated by designer drugs (DREADD) and its artificial ligands, such as clozapine‐N‐oxide (CNO) and deschloroclozapine (DCZ), labeled with ^11^C could be employed for PET of neurons and transplanted iPS cells in animal brains (Ji *et al*, 2016; Nagai *et al*, 2016, 2020). Nonetheless, these compounds cannot be applied to bimodal imaging, and they may also suffer from undesired effects on endogenous substrates (Gomez *et al*, 2017; Haywood *et al*, 2019).

E. coli dihydrofolate reductase (ecDHFR) is an enzyme whose activity is specifically blocked by a small antibiotic, trimethoprim (TMP). TMP rapidly diffuses through tissues, passes the BBB, and, importantly, lacks native targets in mammals (Nau *et al*, 2010; Liu *et al*, 2013). Moreover, TMP can be conjugated with fluorophores, and its radioactive analogs, [^11^C]TMP and [^18^F]TMP, are compatible with PET (Miller *et al*, 2005; Sellmyer *et al*, 2017a, 2017b, 2020). These features make ecDHFR and TMP attractive candidates for the design of versatile probes for PET and fluorescent imaging of intact brains. Here, we construct the first generation of such reporters and characterize their performance in brains of mice and non‐human primates.

## Results

### *In vivo* fluorescence imaging of ecDHFR reporter

**Figure 1:**
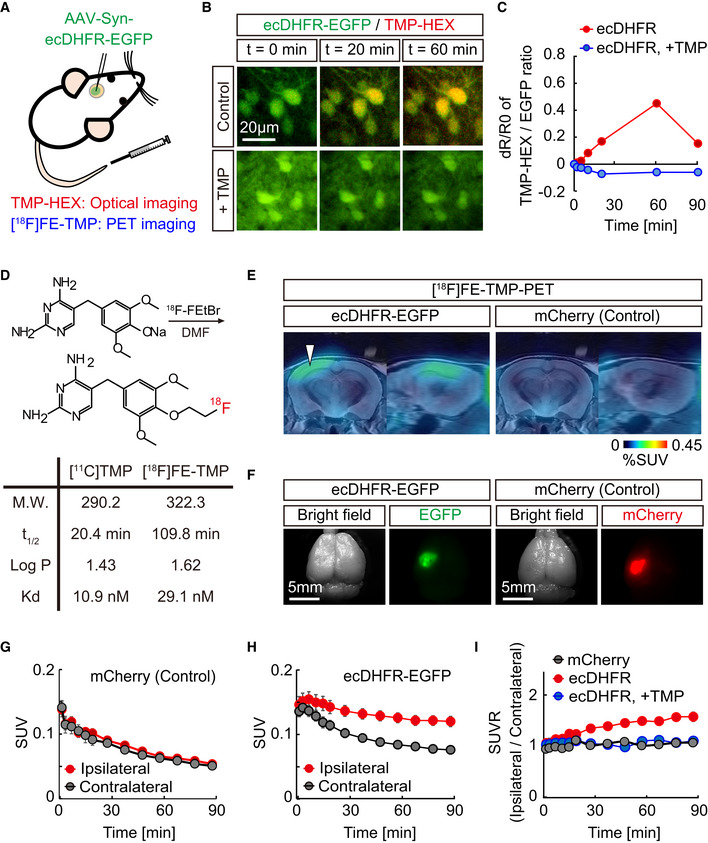
*In vivo* visualization of ecDHFR with TMP‐HEX and [^18^F]FE‐TMP ASchematic illustration of bimodal optical and PET reporter imaging in the brain of living mice. Recombinant ecDHFR‐EGFP reporter proteins were introduced into the somatosensory cortex with AAV. Two‐photon imaging and PET scan were conducted after intravenous administration of TMP analogs, TMP‐conjugated fluorescence dye (TMP‐HEX), and TMP labeled with radioisotope ([^18^F]FE‐TMP), respectively.B, CPeripherally delivered TMP‐HEX penetrates the BBB and labels exogenously expressed ecDHFR in the mouse brain *in vivo*. Brains of awake animals were imaged under a two‐photon microscope following tail vein administration of TMP‐HEX (500 μg/kg). (B) Typical merged images of EGFP (green) and TMP‐HEX (red) fluorescence in mice that were given TMP‐HEX alone (Control) or together with conventional TMP (i.p., 100 mg/kg). Each image is an average of 10 frames from serial z‐stacks. (C) Ratios of TMP‐HEX fluorescence intensity relative to EGFP, plotted as a function of time. Averaged values from two independent experiments (20 cells/2 mice for each condition) are shown.DChemical structure and physical parameters of [^18^F]FE‐TMP compared with conventional [^11^C]TMP.E–IAAVs encoding ecDHFR‐EGFP or mCherry (as control) were unilaterally injected into the somatosensory cortex. PET scans were performed after peripheral administration of radioactive TMP analog, [^18^F]FE‐TMP alone, or [^18^F]FE‐TMP together with conventional TMP (100 mg/kg). (E) Representative PET images (coronal and sagittal sections) generated by averaging dynamic scan data at 60–90 min after i.v. injection of [^18^F]FE‐TMP. Template MRI images were overlaid for spatial alignment. Arrowhead indicates the areas of accumulation of the radioactive ligand in animals carrying ecDHFR‐EGFP (left). Radiosignals around the hypothalamus are considered to be non‐specific, as they were also noted in the control mouse brains. (F) Representative images of postmortem brain tissues carrying ecDHFR‐EGFP (left) or mCherry (right) expression in the somatosensory cortex. (G) [^18^F]FE‐TMP labeling kinetics in the brain of control mice expressing mCherry, plotted as mean ± SEM (*n* = 6). (H) [^18^F]FE‐TMP labeling kinetics in the brain of ecDHFR‐EGFP‐expressing mice (mean ± SEM, *n* = 16). *F*(1, 30) = 8.753; *P* < 0.01 (two‐way, repeated‐measures ANOVA). VOIs were manually placed on ipsilateral and contralateral areas for quantification. SUV data at *t* = 1.5, 3.5, 7, 11, 15, 19, 27.5, 37.5, 47.5, 57.5, 67.5, 77.5, and 87.5 min are plotted. (I) SUVR (SUV ratio) of ipsilateral/contralateral signals in the brain of mCherry‐ (*n* = 6) or ecDHFR‐EGFP‐expressing mice after bolus injection of [^18^F]FE‐TMP alone (*n* = 16), or together with conventional TMP (i.p., 100 mg/kg, *n* = 4). Data at *t* = 1.5, 3.5, 7, 11, 15, 19, 27.5, 37.5, 47.5, 57.5, 67.5, 77.5, and 87.5 min are plotted as mean ± SEM. *F*(2, 23) = 9.778; *P* < 0.05 (two‐way, repeated‐measures ANOVA). Source data are available online for this figure.

**Figure EV1:**
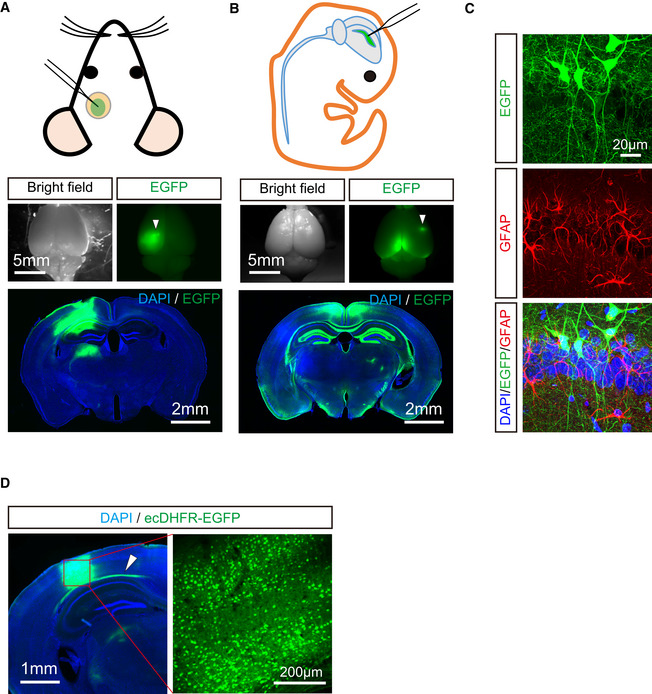
Comparative assessment of transgene expression by AAV injection into rodent brain with two different protocols Schematic diagram of AAV injection into one side of somatosensory cortex with cranial window in adult mouse brain. EGFP expression was introduced under control of synapsin promoter. Spatial distribution of EGFP was analyzed in whole brain (upper) and coronal brain slices (lower). Note that EGFP expression was highly restricted to injection site in this protocol. Arrowhead indicates AAV injection site.Schematic diagram of AAV injection into one side of lateral cerebral ventricle at postnatal day 0. Spatial distribution of EGFP was analyzed in whole brain (upper) and coronal slices (lower). Note that high‐level expression of EGFP is observed in circumferentially arranged area of the ventricle system including corpus callosum, hippocampus, and parietal cortex.Neuron‐specific expression of EGFP under control of synapsin promoter is verified by immunostaining using anti‐GFAP antibody. Representative images of neurons (green) and astrocytes (red) with DAPI staining (blue) in hippocampal CA1 captured by confocal microscopy are displayed.AAVs encoding ecDHFR‐EGFP were unilaterally injected into somatosensory cortex. Enriched neuronal expression of ecDHFR‐EGFP at injection site was assessed by fluorescence imaging of brain slices. Note arrowhead indicates that ecDHFR‐EGFP was also weakly distributed to axonal fibers derived from neurons of injection site.

We hypothesized that ecDHFR, along with TMP, could be a suitable candidate for bimodal optical and PET imaging of the neuronal circuits and their function in intact brains (Fig 1A). To explore this possibility, we first examined the utility of a red fluorophore‐conjugated TMP derivative, TMP‐hexachlorofluorescein (TMP‐HEX), as a probe for optical imaging of ecDHFR in the brains of living mice. We locally introduced ecDHFR‐EGFP into the mouse somatosensory cortex by an adeno‐associated virus (AAV) vector under control of a pan‐neuronal synapsin promoter (Fig EV1A and C). Awake animals were examined by intravital two‐photon laser microscopy after a single bolus tail vein administration of TMP‐HEX. Individual neurons carrying ecDHFR‐EGFP retained TMP‐HEX, indicating that the compound penetrates the BBB similarly to conventional TMP (Fig 1B). Fluorescence signals in these neurons peaked at ∼60 min postinjection, followed by a gradual washout during a 3‐h imaging session (Appendix Fig S1). Importantly, intraperitoneal administration of a saturating dose of unlabeled TMP prior to the imaging session almost completely blocked the binding of TMP‐HEX to ecDHFR, supporting the specificity of TMP‐HEX for ecDHFR *in vivo* (Fig 1B and C, Appendix Fig S1).

### PET imaging of ecDHFR with radioactive TMP analogs

**Figure EV2:**
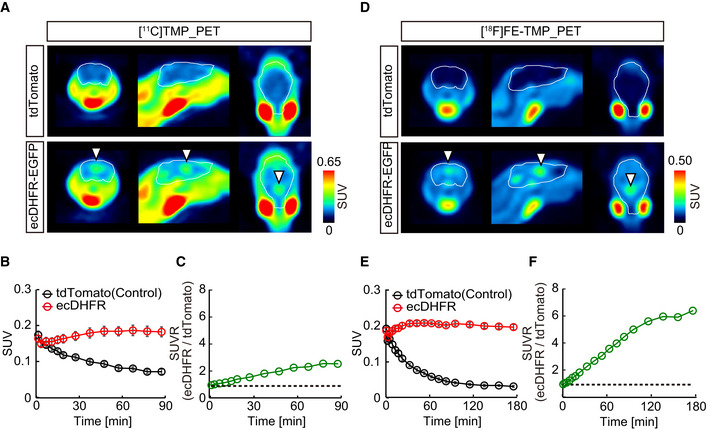
PET imaging of ecDHFR in mouse brain with radioactive TMP analogs Mice expressing ecDHFR‐EGFP or tdTomato (as control) from AAVs in forebrain were subjected to PET scans after peripheral administration of radioactive TMP analogs [^11^C]TMP and [^18^F]FE‐TMP (40 MBq/mouse).
ARepresentative PET images (coronal, sagittal, and horizontal sections from left) generated by averaging dynamic scan data at 0–90 min after i.v. injection of [^11^C]TMP. Arrowheads indicate areas of accumulation of radioactive ligand in animals carrying ecDHFR‐EGFP (lower). White lines mark whole brain area as determined by MRI.B[^11^C]TMP labeling kinetics. Volumes of interest (VOI) of fixed sizes were manually placed on paraventricular region exhibiting high‐level radioactive signals. Data from control (*n* = 7) and ecDHFR‐EGFP‐expressing mice (*n* = 7) were plotted as mean ± SEM. *F*(1, 12) = 22.05; *P* < 0.01 (two‐way ANOVA).CRatios of averaged [^11^C]TMP radioactive signals in ecDHFR versus control brains.DRepresentative PET images (coronal, sagittal, and horizontal sections from left) generated by averaging dynamic scan data at 0–180 min after i.v. injection of [^18^F]FE‐TMP. Arrowheads indicate areas of accumulation of radioactive ligand in animals carrying ecDHFR‐EGFP (lower). White lines mark whole brain.E[^18^F]FE‐TMP labeling kinetics. VOI analysis was performed as described in panel c. Data from control (*n* = 6) and ecDHFR‐EGFP‐expressing mice (*n* = 6) were plotted as mean ± SEM. *F*(1, 10) = 326.1; *P* < 0.01 (two‐way ANOVA).FRatios of averaged [^18^F]FE‐TMP radioactive signals in ecDHFR versus control brains. Source data are available online for this figure.

To accomplish an *in vivo* macroscopic analysis of ecDHFR distribution in the brain by PET, we next radiosynthesized [^11^C]TMP and characterized its performance in a PET analysis of mice that expressed ecDHFR‐EGFP or red fluorescent protein, tdTomato, as a control generated by a single AAV injection into cerebral lateral ventricles at birth (Fig 1D). At 2 months of age, mice were given [^11^C]TMP via tail vein bolus injection and subjected to dynamic PET scans. While animals carrying tdTomato exhibited minimal accumulation of [^11^C]TMP radioactivity, mice expressing ecDHFR‐EGFP had strong radiosignals in the brain (Fig EV2A–C). Quantitative kinetic measurements revealed that [^11^C]TMP detection contrast was maintained over the course of 90‐min scanning sessions (Fig EV2B). Moreover, the ratio of radioactive signals between ecDHFR and control brains increased up to 2.5 at the end of dynamic scans, suggesting that a static scan within this time frame would suffice for tracing of a target protein (Fig EV2C). Considering the longer half‐life of ^18^F, we further synthesized an ^18^F‐labeled TMP derivative, [^18^F]fluoroethoxy‐TMP ([^18^F]FE‐TMP) (Fig 1D). Although the dissociation constant of [^18^F]FE‐TMP (∼30 nM) against recombinant ecDHFR was slightly higher than that of [^11^C]TMP (∼10 nM), the PET imaging contrast for ecDHFR was profoundly improved when mice were given [^18^F]FE‐TMP. The ratio of radioactive signals between ecDHFR and control brains increased up to ∼6.0 in a steady state, and this was primarily attributed to a much faster washout of unbound [^18^F]FE‐TMP from the brain after initial uptake (Fig EV2D–F). The distribution of radioactive signal reasonably matched the uniform and enriched expression patterns of the reporter regions adjacent to ventricles, including the retrosplenial cortex, hippocampus, and ventral parts of the brain (Fig EV1B).

We also unilaterally delivered AAVs encoding ecDHFR‐EGFP or red fluorescent protein, mCherry, as a control into the somatosensory cortex of mice, and we conducted PET scans with [^18^F]FE‐TMP 1 month after the viral injections. Remarkably, mice expressing ecDHFR‐EGFP exhibited strong radiosignals on an ipsilateral side of the somatosensory cortex locally delivered with AAV encoding ecDHFR‐EGFP, but not in uninfected areas of the contralateral side or ipsilateral side expressing mCherry (Fig 1E, F, G and H). The SUVR value of ipsilateral/contralateral signals was highly correlated with BP_ND_ determined by Logan reference models (Appendix Fig S2). Pre‐administration of unlabeled TMP significantly attenuated the enriched radioactive signals of [^18^F]FE‐TMP on the ipsilateral side expressing ecDHFR‐EGFP (Fig 1I), indicating saturable and specific binding of [^18^F]FE‐TMP to ecDHFR. Taken together, these results demonstrate that TMP analogs can be used for multiscale optical and PET imaging of ecDHFR‐based reporters in the brains of live animals.

### Binding specificity of TMP analogs and metabolic profile of [^18^F]FE‐TMP

**Figure EV3:**
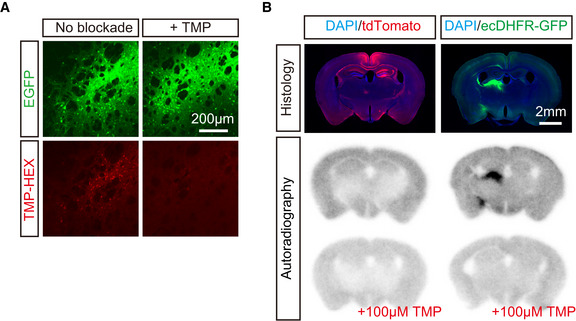
*In vitro* validation of TMP‐HEX and [^18^F]FE‐TMP for ecDHFR Representative images of striatal neurons expressing high‐level ecDHFR‐EGFP in fixed brain slices labeled with TMP‐HEX. Note that incubation with excess amount of non‐labeled TMP markedly inhibits fluorescence labeling.
*In vitro* autoradiography of mouse brain sections with [^18^F]FE‐TMP. Expression of transgenes in samples collected from mice treated with control and ecDHFR‐EGFP vectors is fluorescently visualized with DAPI counterstaining (upper). Representative images of *in vitro* autoradiography using [^18^F]FE‐TMP demonstrate that this radioligand specifically labels brain areas overexpressing ecDHFR‐EGFP but not tdTomato (middle), and that this radioligand binding to putative ecDHFR is profoundly blocked by an excess amount of non‐labeled TMP (lower).

To obtain further evidence to support our *in vivo* imaging data on the specificity of fluorescent and radioactive TMP analogs for ecDHFR, we examined *in vitro* heterologous blockade of the reporter labeling by a well‐characterized ecDHFR ligand, TMP. Readouts of both fluorescence microscopy and autoradiography consistently demonstrated that, in brain sections derived from mice carrying ecDHFR‐EGFP, TMP‐HEX (Fig EV3A), [^11^C]TMP (data not shown), and [^18^F]FE‐TMP (Fig EV3B) were selectively retained in areas of transgene expression and their detection was strongly suppressed in the presence of saturating amounts of unconjugated or non‐radiolabeled compound.

**Figure EV4:**
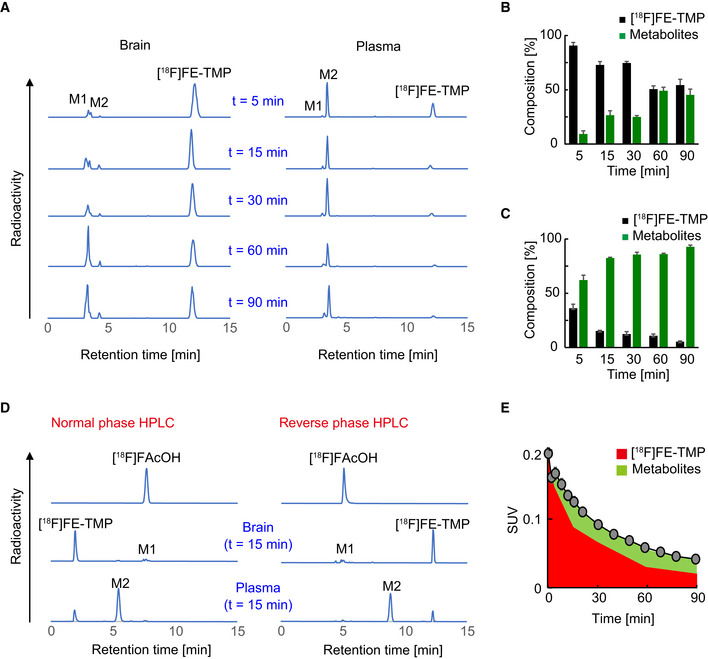
Characterization of [^18^F]FE‐TMP radioactive metabolites ARepresentative reverse phase radio‐HPLC charts showing [^18^F]FE‐TMP and its radiometabolites, M1 and M2, in brain (left) and plasma (right) of mice at 5, 15, 30, 60, and 90 min after i.v. injection of [^18^F]FE‐TMP. M1 and M2 are detected as major radiometabolites in brain and plasma, respectively.B, CTime‐course changes in the composition of [^18^F]FE‐TMP and its radiometabolites in brain (B) and plasma (C). Relative amounts of radiomaterials are indicated as % of total radioactivities at each time point. Data from 3 mice at each time point were plotted as mean ± SD.DIdentification of a major metabolite, M1, by normal (left) and reverse (right) phase radio‐HPLC. Retention times in HPLC charts were compared between radiosynthesized [^18^F]fluoroacetate ([^18^F]FAcOH) and radioactive metabolites derived from [^18^F]FE‐TMP in mouse brain and plasma at 15 min after i.v. administration of [^18^F]FE‐TMP. Retention time of [^18^F]FAcOH was identical to that of M1, a major radiometabolite of [^18^F]FE‐TMP in brain.ERadioactivity derived from [^18^F]FE‐TMP (red area) and its radiometabolites (green area) in control cortex was calculated by applying temporal changes in their relative abundance (B) to the time‐radioactivity curve shown in Fig EV2E. Data from control mice (*n* = 6) were plotted as mean ± SEM. Source data are available online for this figure.

We also assessed the metabolic profile of [^18^F]FE‐TMP in wild‐type mice by radio‐HPLC and identified its two major radiometabolites (M1 and M2) (Fig EV4A). M2 was present in plasma; its fraction reached a plateau at 15 min after i.v. injection of [^18^F]FE‐TMP and did not undergo efficient transfer to the brain (Fig EV4C). In contrast, M1 was a major radioactive metabolite in the brain, and its fraction gradually increased over 90 min after i. v. injection of [^18^F]FE‐TMP (Fig EV4B). We identified M1 as [^18^F]fluoroacetate ([^18^F]FAcOH; see Fig EV4D), *in vivo* characteristics of this radiochemical were analyzed in previous work (Mori *et al*, 2009). Estimated accumulation of [^18^F]FAcOH in the brain was relatively slow (Fig EV4E), and this metabolite should not react with ecDHFR in consideration of its chemical structure. Hence, it was unlikely that [^18^F]FAcOH noticeably contributed to the radiosignal retentions in the brain as assessed by PET.

### PET monitoring of neuronal ensemble activities

**Figure 2:**
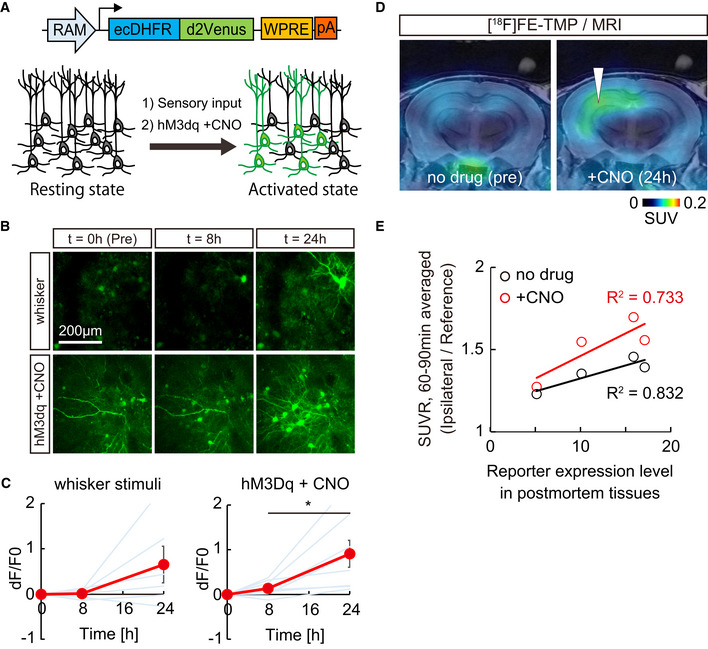
*In vivo* visualization of neuronal ensemble activation in response to sensory stimuli and hM3Dq‐DREADD ASchematic illustration of virus construction and marking of neuronal ensemble activation by CNO‐mediated hM3Dq‐DREADD activation.B, CAAVs encoding ecDHFR fused to destabilized Venus (ecDHFR‐d2Venus) with RAM promoter and hM3Dq were co‐introduced into two segregated regions of the somatosensory cortex and imaged under two‐photon microscope. (B) Fluorescent intensity of ecDHFR‐d2Venus in the brain of awake animals was analyzed before (0 min) and after (8 or 24 h) air‐puff mediated whisker stimulation (10 s, five times) or chemogenetic activation of hM3Dq via peripheral administration of CNO (10 mg/kg). Averaged images of 10 stacked frames in serial z position are shown. (C) Averaged ecDHFR‐d2Venus fluorescence intensities, plotted as dF/F0 ratios at different time points after neuronal activation. Data from 6–8 regions / 3–4 mice for each condition are plotted as mean ± SD (red line) and individual regions (blue lines). 8 h vs 24 h, *F*(2, 21) = 7.715; *P < 0.05 (one‐way ANOVA).D, EMice expressing ecDHFR‐d2Venus and hM3Dq in a side of hippocampus was sequentially analyzed by [^18^F]FE‐TMP PET imaging before (left) and 24 h after (right) intraperitoneal CNO injection. To avoid epileptic seizures and immediate death of mice, we used a minimized dose of CNO (0.3 mg/kg) in this experimental condition. About 2 weeks after PET scan, the mice were sacrificed for histochemical analysis. To determine the expression level of the reporter, postmortem brain slice images, as shown in Appendix Fig S3B, were captured by confocal microscope, ROIs were manually placed on the hippocampal region, and averaged fluorescence intensity of ecDHFR‐d2Venus was measured. The background value of non‐infected hippocampal region was set as 1. (D) Representative PET images demonstrate that 0.3 mg/kg CNO‐mediated activation of hM3Dq enhanced the accumulation of radioactive signals in the hippocampus. Averaged images of dynamic scan data at 60–90 min after i.v. injection of [^18^F]FE‐TMP are shown. Template MRI images were overlaid for spatial alignment. Arrowhead indicates selective accumulation of radioactive signals in the AAV injection site after CNO administration. (E) SUVR (SUV ratio) of ipsilateral hippocampi / reference region (brain stem) signals during dynamic PET scans. Data from ecDHFR‐d2Venus expressing mice (*n* = 4) before and after 0.3 mg/kg CNO i.p. injection are plotted. Correlation with *R* ^2^ values between SUVR of [^18^F]FE‐TMP_PET (y‐axis) and relative expression levels of ecDHFR‐d2Venus (x‐axis, fluorescence) in postmortem tissues was determined by linear regression analysis. Source data are available online for this figure.

To take advantage of the advanced utility of ecDHFR reporter for PET monitoring of neural circuit activations, we incorporated the robust activity marking (RAM) system, an optimized synthetic activity‐regulated element with the immediate early gene (IEG) cFos minimal promoter, which can drive the expression of a reporter protein in response to sensory stimuli and epileptic seizure (Sorensen *et al*, 2016) (Fig 2A). RAM‐dependent efficient marking of neuronal activation was successfully validated in cultured neurons (Appendix Fig S3A). We then co‐introduced AAVs encoding ecDHFR‐d2Venus with RAM promoter and the excitatory DREADD, hM3Dq, into one side of the somatosensory cortex and examined the time‐course change in the reporter expression by two‐photon laser microscopy after air‐puff stimulation of whiskers or hM3Dq activation via an intraperitoneal bolus administration of CNO. Repetitive sensory inputs by air‐puff stimulation of whiskers robustly enhanced the fluorescence signals of ecDHFR‐d2Venus in neurons, whereas the activation of hM3Dq more prominently induced signal enhancement in neurons expressing ecDHFR‐d2Venus at 24 h after systemic administration of CNO (Fig 2B and C). We then prepared mice co‐expressing RAM‐ecDHFR‐d2Venus and hM3Dq on one side of hippocampi and tested whether PET can successfully capture CNO‐provoked activations of a neural circuit. Remarkably, sequential PET scans with [^18^F]FE‐TMP demonstrated significant accumulation of [^18^F]FE‐TMP radiosignals at 24 h after injection of CNO (Fig 2D and E). A postmortem analysis of brain tissues collected from these mice following PET experiments demonstrated induced expression of ecDHFR‐d2Venus primarily in the hippocampal dentate gyrus and CA3 neurons (Appendix Fig S3B), and there was good correlation between PET signals and reporter expressions (Fig 2E). These data indicate that [^18^F]FE‐TMP PET successfully captured the chemogenetic activation of the hippocampal network with high sensitivity and specificity.

### *In vivo* illumination of a deep neural circuit

**Figure 3:**
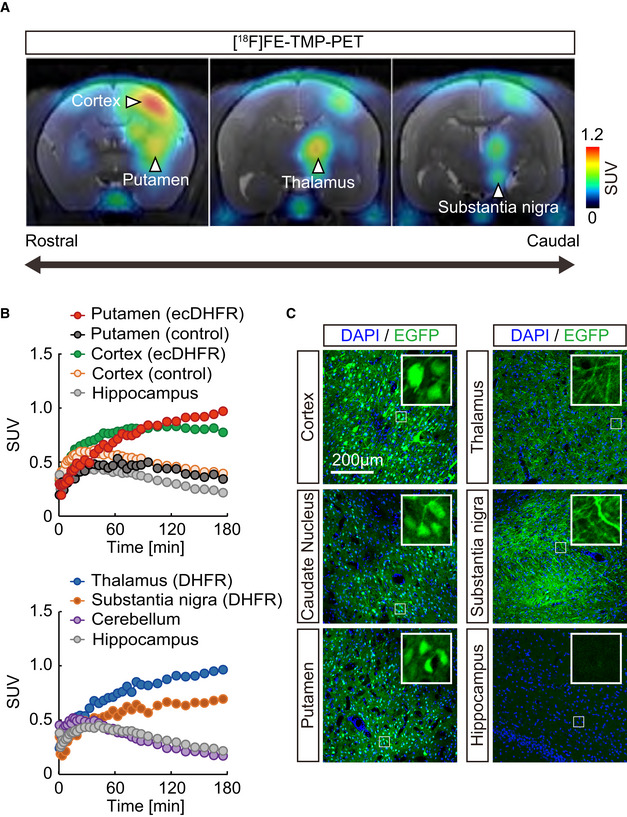
PET imaging of ecDHFR/TMP reporters in primate brain ecDHFR‐EGFP was expressed in the brain of a 3.4‐year‐old common marmoset with an AAV. PET scans of [^18^F]FE‐TMP were performed 45 days after virus injection.
Representative coronal PET images generated by averaging dynamic scan data at 60 ‐ 180 min after i.v. injection of [^18^F]FE‐TMP. Note that reporter molecules were densely distributed in thalamus and substantia nigra pars compacta, which are connected to the neocortex and putamen via direct neuronal tract, respectively. PET images are overlaid with an individual MRI. Scale bar represents SUV.Time‐radioactivity curves for [^18^F]FE‐TMP in the putamen carrying ecDHFR‐EGFP (red symbols) or control AAV (black symbols), neocortex carrying ecDHFR‐EGFP (green symbols) or control AAV (orange symbols), and hippocampus (gray symbols) are displayed in the upper panel. Curves in the thalamus (blue symbols), substantia nigra (orange symbols), and cerebellum (purple symbols) along with hippocampus data are also shown in the lower panel.Postmortem analysis of ecDHFR‐EGFP expression in brain slices. Representative images illustrate EGFP fluorescence in the cortex, caudate nucleus, putamen, thalamus, substantia nigra, and hippocampus. High‐magnification image frames are shown in inserts. Source data are available online for this figure.

We next sought to determine whether our imaging techniques can be leveraged to trace neural circuits in deep brain regions of live non‐human primates in greater detail than in rodents. Given the similarity of anatomies of monkey and human brains, we reasoned that this approach could further validate the current method for future medical applications. An AAV encoding ecDHFR‐EGFP was delivered to one side of the neocortex and striatum of a 3.4‐year‐old male common marmoset to express the reporter in the telencephalon. PET scans of this animal with [^18^F]FE‐TMP were performed 45 days after virus injection. To our surprise, the PET scans revealed robust accumulation of [^18^F]FE‐TMP not only in brain regions proximal to the injection site (i.e., neocortex, caudate nucleus, and putamen) but also in other spatially segregated but interconnected areas, including the thalamus and midbrain substantia nigra (Fig 3A). The marmoset brain displayed more efficient radioligand uptake than the mouse brain, and the radioactive signals of the cortex and putamen increased up to 4 times higher than that of the hippocampus at the end of the dynamic PET scan (Fig 3B and Appendix Fig S9). Again, distribution of the radioactive signal was consistent with the localization of transgene expression, as assessed by postmortem microscopic imaging of brain sections (Fig 3C). Accordingly, it is likely that the current ecDHFR‐based reporters, particularly in combination with the RAM system, are capable of pursuing chemogenetic activation of a specific neural connection in non‐human primates, which have been technically enabled by DREADD and DCZ in our recent work (Nagai *et al*, 2020).

### PET analysis of protein turnover with ecDHFR mutant

**Figure 4:**
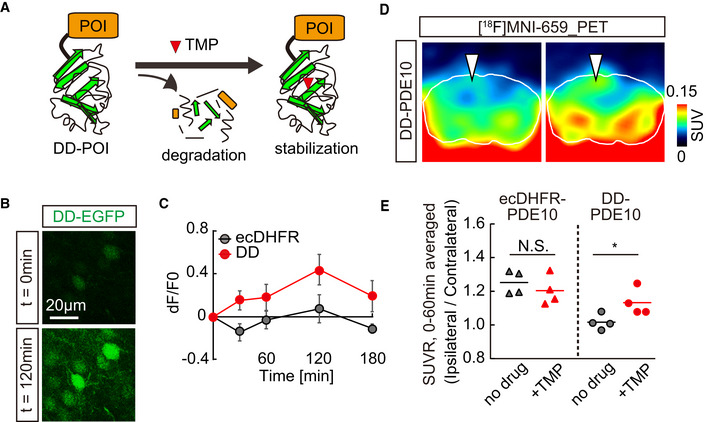
PET analysis of TMP‐inducible protein stabilization ASchematic illustration of TMP‐dependent control of DD‐POI protein turnover. A protein of interest (POI) fused to a destabilizing domain (DD) undergoes rapid degradation by ubiquitin‐proteasome system, while POI can be stabilized in the presence of TMP.B, CMice expressing fusion proteins comprised of EGFP and either wild‐type or destabilized mutant ecDHFR (ecDHFR‐EGFP or DD‐EGFP, respectively) were imaged by two‐photon microscope. The constructs were introduced into the somatosensory cortex with AAVs. (B) Fluorescent intensity of DD‐EGFP in intact brains of awake animals was analyzed before (0 min) and after (120 min) peripheral administration of TMP (100 mg/kg). Averaged images of 10 stacked frames in serial z position are shown. (C) Averaged ecDHFR‐EGFP and DD‐EGFP fluorescence intensities plotted as dF/F0 ratios at different time points after TMP delivery. Data from 10 neurons for each genotype are plotted as mean ± SD. *F*(1, 18) = 64.36; *P* < 0.01 (two‐way, repeated‐measures ANOVA).D, EecDHFR‐PDE10 and DD‐PDE10 were expressed in a caudal region of cortical hemisphere from an AAV. Accumulation of the radioactive signal was monitored by PET scan with [^18^F]MNI659, a selective radioactive PET ligand against PDE10. (D) Representative PET images demonstrate the detection of TMP‐stabilized DD‐PDE10 in caudal area of neocortex. Averaged images of dynamic scan data at 30–60 min after i.v. injection of [^18^F]MNI659 are shown. 90‐min scans were conducted before (left) or 2 h after (right) intraperitoneal TMP injection (100 mg/kg). White lines mark the whole brain area as determined by MRI. Note selective accumulation of radioactive signals in the AAV injection site by TMP administration (arrowheads). (E) Averaged SUVR (SUV ratio) of ipsilateral/contralateral radioactive signals during 0–60‐min scans in individual animals expressing ecDHFR‐PDE10 (*n* = 4) or DD‐PDE10 (*n* = 4). Data represent mean (horizontal bar) and values from individual animals (dots) for each group under control condition (no drug) or after TMP treatment. *t*(6) = 2.521; *P < 0.05 (Student’s *t*‐test). Note a significant TMP‐dependent increase in the ratio in animals carrying a destabilized reporter. Source data are available online for this figure.

In the final set of experiments, we aimed to explore further applications of unique ecDHFR mutants for PET assays of the protein dynamics in the brain circuit. To this end, we employed an ecDHFR mutant with a destabilized domain (DD) that enabled us to control gene expression and function of a protein of interest (POI) fused to DD in a manner inducible by TMP administration (Fig 4A and Appendix Fig S4) (Sando *et al*, 2013; Pieraut *et al*, 2014). In agreement with previous studies, treatments with TMP rapidly restored the levels of DD‐EGFP in the mouse brain (Fig 4B and C). We subsequently utilized a cyclic phosphodiesterase, PDE10A, as a POI, which can be detected by PET with a specific radioligand, [^18^F]MNI659 (Russell *et al*, 2014). The catalytic domain of human PDE10A (hPDE10A_CD; Appendix Fig S5) was incorporated in AAVs encoding hPDE10A_CD‐EGFP with N‐terminal ecDHFR or DD tags and was inoculated into the neocortex. PET scans demonstrated a significant accumulation of [^18^F]MNI659 in the ipsilateral area expressing DD‐hPDE10A_CD‐EGFP following peripheral delivery of TMP, suggesting that PET can be used to monitor acute drug‐inducible stabilization of DD‐POIs in a brain circuit (Fig 4D and E). Hence, the current technology would serve for manipulation of molecular signaling by switching POI on and off and for assessing the functionality of protein degradation systems in healthy and diseased neural networks.

### PET analysis of protein complex formation

**Figure 5:**
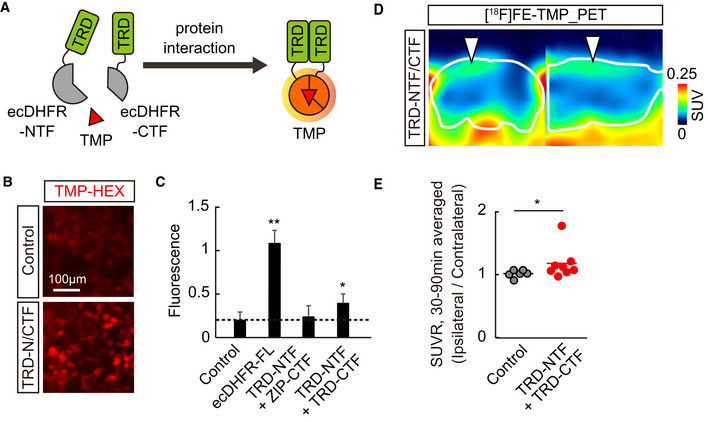
PET analysis of protein–protein interaction in brain ASchematic representation of reporter containing N‐ and C‐terminal ecDHFR fragments (NTF and CTF). These fragments reassemble after interaction of fused human tau repeat domain (TRD), thereby restoring TMP‐binding ability.BRepresentative images illustrate selective retention of TMP‐HEX in cells expressing ecDHFR‐NTF and ecDHFR‐CTF fused with human tau repeat domain (TRD).CTRD oligomerization‐dependent TMP‐HEX labeling was quantitatively analyzed in cells expressing various combinations of ecDHFR‐NTF and CTF fragments as normalized fluorescence intensities. Mean value of full‐length ecDHFR (ecDHFR‐FL) was set as 1. Data from seven independent experiments are shown as mean ± SD. *F*(3, 24) = 77.86; **P < 0.01, *P < 0.05 (one‐way ANOVA followed by Dunnett post hoc test).D, EPET analysis of Tau oligomerization in the brain. 90‐min [^18^F]FE‐TMP PET scans were conducted after co‐injection of AAVs encoding TRD‐NTF and TRD‐CTF into one hemisphere of somatosensory cortex. (D) Representative PET images (coronal and sagittal sections from left) were generated by averaging dynamic scan data at 30‐90 min after i.v. injection of [^18^F]FE‐TMP. Images show selective accumulation of radioactive signals in AAV injection site (arrowheads). White lines mark whole brain area as determined by template MR image. (E) Averaged SUVR (SUV ratio) of ipsilateral/contralateral radioactive signals during 30‐ to 90‐min dynamic PET scans. Data from control mice (*n* = 6; mixture of uninfected mice, *n* = 4 and TRD‐NTF/ZIP‐CTF‐expressing mice, *n* = 2) or TRD‐NTF/TRD‐CTF‐expressing mice (*n* = 8) are plotted as mean (horizontal bar) and individual animals (dots). *P < 0.05 (Mann–Whitney *U*‐test). Source data are available online for this figure.

**Figure EV5:**
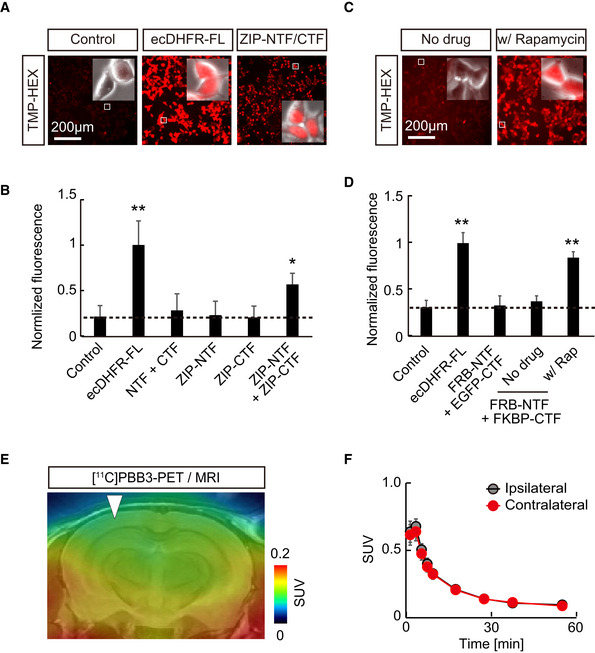
Design and characterization of protein fragment complementation assay using a split DHFR system A–DCultured HEK293 cells expressing various constructs were incubated with 100 nM TMP‐HEX and analyzed by fluorescent microscopy. TMP‐HEX efficiently labeled cultured cells co‐expressing NTF and CTF conjugated to a self‐assembling leucine zipper (ZIP) motif, or a rapamycin‐dependent heterodimerization motifs FKBP12‐rapamycin binding domain (FRB) and FK506 binding protein (FKBP) in the rapamycin‐dependent manner, indicating that the cDHFR‐PCA system functions under these conditions as designed. (A) Representative images illustrate a selective retention of TMP‐HEX in cells carrying full‐length ecDHFR (as positive control) or a combination of ZIP‐tagged ecDHFR‐NTF and ecDHFR‐CTF. Insets demonstrate high‐magnification fluorescence images overlaid with phase‐contrast pictures of individual cells. Note that ZIP‐tagged ecDHFR‐NTF and CTF preferentially localize in nucleus. (B) Normalized fluorescence intensities of TMP‐HEX in cells transfected with indicated constructs. Mean value of full‐length ecDHFR (ecDHFR‐FL) was set as 1. Note that only ecDHFR‐FL and the split protein that reassembles via ZIP interaction retain the marker above background levels. Data from six independent experiments are plotted as mean ± SD. *F*(5, 28) = 21.12; *P < 0.05, **P < 0.01 (one‐way ANOVA followed by Dunnett post hoc test). (C) HEK293 cells expressing various constructs were incubated with 100 nM TMP‐HEX with or without 500 nM rapamycin (LC Laboratories) and imaged by fluorescence microscopy. Representative images illustrate selective labeling of TMP‐HEX in cells co‐expressing ecDHFR‐NTF and CTF tagged with FRB and FKBP in the presence of rapamycin. Insets demonstrate high‐magnification fluorescence photomicrographs overlaid with phase‐contrast images. (D) Rapamycin (Rap)‐induced FRB‐FKBP interaction was assessed as TMP‐HEX labeling efficiency in cells expressing various combinations of ecDHFR‐NTF and ecDHFR‐CTF fragments. Fluorescence intensities were normalized by mean value of labeling of full‐length ecDHFR (ecDHFR‐FL) with TMP‐HEX. Data from four independent experiments are presented as mean ± SD. *F*(4, 15) = 55.08; **P < 0.01 (one‐way ANOVA followed by Dunnett post hoc test).EA representative coronal PET image captured with [^11^C]PBB3, a potent PET tracer for aggregated tau fibrils, in mice expressing TRD‐NTF and TRD‐CTF by AAV injection into one side of somatosensory cortex. Averaged image of dynamic scan data at 0‐60 min after i.v. injection of [^11^C]PBB3 is shown. A template MRI image was overlaid for spatial alignment of the PET image. Arrowhead indicates injection site.FKinetics of [^11^C]PBB3 in mouse brain during 60‐min dynamic PET scan. VOIs were manually placed on ipsilateral and contralateral cortical areas for quantification. Data from four mice expressing TRD‐NTF and TRD‐CTF are plotted as mean ± SEM. Note there is no significant difference in radioactive signals of [^11^C]PBB3 between ipsilateral and contralateral sides. Source data are available online for this figure.

We also relied on a protein fragment complementation assay (PCA) to clarify the applicability of ecDHFR split mutants for monitoring the oligomerization of misfolded microtubule‐associated protein tau, which may propagate through the neural network, leading to neurodegeneration in the pathological cascade of Alzheimer’s disease (Shekhawat & Ghosh, 2011; Goedert *et al*, 2017). We constructed an ecDHFR‐PCA system in which the N‐ and C‐terminal fragments of the ecDHFR protein (NTF and CTF, respectively) reassemble, thereby restoring its capability to bind to TMP, through interaction between a bait and prey (Figs 5A and EV5A–D). As a proof‐of‐principle experiment, TMP‐HEX efficiently labeled cultured cells co‐expressing NTF and CTF conjugated to a human tau repeat domain (TRD), suggesting that the self‐assembly of TRD was spontaneously initiated and could be detected by our method *in vitro* (Fig 5B and C). We therefore co‐injected AAVs encoding TRD‐NTF and TRD‐CTF in the somatosensory cortex, and we then conducted PET scans with [^18^F]FE‐TMP to detect potential tau oligomerization and propagation *in vivo* more than 1 month after the surgical procedure. Remarkably, we found an accumulation of [^18^F]FE‐TMP in the proximity of the inoculation site (Fig 5D), and an ipsilateral‐to‐contralateral ratio of PET signals in a steady state was significantly increased in brains of these mice relative to control brains (Fig 5E). A PET scan with [^11^C]PBB3, a potent PET tracer for fibrillary deposits of pathological tau proteins (Maruyama *et al*, 2013), did not reveal any significant accumulation of radioactive signals in the areas receiving the AAV delivery (Fig EV5E and F). Hence, PET scans with [^18^F]FE‐TMP could capture low‐order, PBB3‐undetectable tau assemblies, which potentially propagate along a specific brain circuit in the brains of intact animals.

## Discussion

In summary, we have designed and validated new ecDHFR‐based reporters and their ligands for complementary fluorescence and PET analyses of the spatial distribution, stability, and aggregation of genetically targeted fusion proteins in brain circuits of live animals. These proteins can be labeled with structurally similar TMP analogs that penetrate the BBB and selectively bind to ecDHFR with high affinity. Our approaches enable high‐contrast imaging and operations of a specific molecule at a network level in physiological and pathophysiological conditions, which is a task that could not be easily accomplished by conventional techniques (Yaghoubi *et al*, 2012). In conjunction with prior studies showing that neither overexpression of ecDHFR nor repetitive treatments with TMP affect neuronal physiology and animal behavior (Iwamoto *et al*, 2010; Sando *et al*, 2013), our findings will facilitate the design of new biosensors for basic and translational neuroscience research.

The recent growing availability of multi‐photon excitation techniques has enabled intravital fluorescence imaging of structural and physiological parameters of neuronal cells in animal brains, which can typically be captured up to a depth of 250–500 μm with a 1–2 mm^2^ field of view. While the optical diffusion limits its applicability onto the surface of the neocortex even in the mouse brain, this technical bottleneck can be overcome with PET technology that is especially adept at quantifying pharmacokinetics and pharmacodynamics of the radioactive tracers in deep neuronal circuits across the whole brain. Although the spatiotemporal resolution of PET imaging is still limited (< 1 mm for spatial and seconds for temporal in animal PET), advanced features of PET provide informative insight regarding the macroscopic dynamics of the biological target in living subjects with a non‐invasive manner, indicating its superior utility for biomedical and clinical studies (Piel *et al*, 2014). Therefore, bimodal fluorescence and PET neuroimaging enable simultaneous tracking of a target molecule of interest in a broad range of views and promises to be a suitable approach for future translational research. We recently demonstrated that tau depositions in a tauopathy model mouse can be visualized with tau PET tracers on a scale of subcellular to whole brain by bimodal two‐photon microscope and PET imaging, providing important insight into the sensitivity and disposition of these unique compounds (Tagai *et al*, 2021).

The acute interactions of peripherally delivered TMP analogs with neuronal ecDHFR documented herein are in agreement with previous reports that conventional TMP rapidly diffuses through tissues and accumulates in the brain within minutes after intraperitoneal injections (Iwamoto *et al*, 2010; Sando *et al*, 2013). Of note, the uptake of [^11^C]TMP and [^18^F]FE‐TMP in the brain was lower than that of widely used PET tracers, such as [^11^C]raclopride (Takuwa *et al*, 2015; Sellmyer *et al*, 2017a). Moreover, our two‐photon imaging experiments suggest that, similar to [^11^C]TMP and [^18^F]FE‐TMP, TMP‐HEX labels neurons with relatively slow binding kinetics. Nonetheless, all three ligands permitted reliable detection of ecDHFR in the brain, presumably due to slow dissociation from the binding site. Despite the dissociation constant of [^11^C]TMP against ecDHFR being better than that of [^18^F]FE‐TMP, our PET imaging revealed that unbound [^18^F]FE‐TMP undergoes faster washout in a non‐displaceable compartment and produces a higher image contrast than [^11^C]TMP (Fig EV2), indicating that this probe may be a better choice for PET. The *in vivo* contrast for site‐directed radiotracer binding could depend on the *B*_max_/*K*_d_ value and the levels of background (non‐displaceable) tracer retentions. According to previous reviews (Patel & Gibson, 2008; McCluskey *et al*, 2020), *B*_max_/*K*_d_ ≧ 10 is considered preferable for *in vivo* imaging, whereas several radioligands as exemplified by [^11^C]cocaine and [^11^C]raclopride with *in vitro* and *in vivo* Bmax/Kd values below 10 have been utilized for PET studies. The non‐displaceable binding potential of [^18^F]FE‐TMP, which could be equivalent to Bmax/Kd and could be estimated as [(target‐to‐reference ratio of radiotracer retention) – 1.0], was ∼5.0 in the mouse brain following intraventricular AAV injection (Fig EV2F) and may vary as a function of ecDHFR expression levels. Considering the suitability as CNS PET tracers, [^18^F]FE‐TMP is still in its first generation and has areas that need to be improved in terms of clearance half‐time and equilibrium conditions. Since the pharmacokinetics of the tracer along with the reactivity of ecDHFR can be greatly improved by further chemical and protein engineering, continuous future effort to refine the performance of the reporter system would explore a broader range of applications in neuroscience and biomedical research.

As for further improvement of the reporter performance, the susceptibility to efflux transporters represented by P‐glycoprotein (P‐gp) essential for BBB penetration of the compounds and the radioactive metabolic profile involved in off‐target radiosignals are also important issues. Regarding the former, our preliminary experiment demonstrated that the brain uptake of [^18^F]FE‐TMP was significantly enhanced by pre‐treatment of animals with 3mg/kg Elacridar, a potent inhibitor of P‐gp (Kuntner *et al*, 2010), indicating that [^18^F]FE‐TMP is a substrate of P‐gp‐mediated efflux activity (Appendix Fig S6). In mice, half of the [^18^F]FE‐TMP radiosignals was cleared from the brain approximately 30 min after their peak uptake (Fig EV2E), which may be suitable for imaging of the target with reasonably high contrasts. In a marmoset, the radiotracer uptake was higher, but the radioactivity clearance was slower than in mice, raising the possibility that the marmoset P‐gp reacts with [^18^F]FE‐TMP more weakly than does the murine P‐gp. This implies that the chemical modification of TMP to minimize the P‐gp susceptibility may drastically improve its utility as PET tracer. In contrast, regarding the latter case, the major contribution of [^18^F]FE‐TMP to PET radiosignals in ecDHFR‐rich regions was also indicated by the present metabolic analyses (Fig EV4). We learned that [^18^F]FAcOH is the almost sole radiometabolite of [^18^F]FE‐TMP existing in the brain, which is structurally dissimilar to [^18^F]FE‐TMP and should therefore be unreactive to ecDHFR. Although a previous report implied possible accumulations of [^18^F]FE‐TMP in reactive astrocytes (Marik *et al*, 2009), marked effects of local astrogliosis on imaging data were ruled out by homogeneously low retentions of radiosignals throughout the brains of mice receiving a topical injection of AAVs encoding mCherry.

Recently, rapidly degrading ecDHFR mutants have been exploited for TMP‐inducible control of LoxP recombination in the brain with destabilized Cre (Sando *et al*, 2013; Pieraut *et al*, 2014). We were unable to directly visualize DD‐EGFP by PET with [^18^F]FE‐TMP, perhaps due to transient stabilization and/or low copy of the protein. However, the proof‐of‐principle experiments in which hPDE10_CD was simultaneously stabilized with TMP and visualized with [^18^F]MNI659 suggest that construction of sensitive DD‐POI PET reporters is technically feasible. When driven by cell type‐specific or activity‐regulated promoters, ecDHFR and DD reporters may become instrumental for understanding how various biologically active molecules as represented by optogenetic and chemogenetic tools acutely impact neural circuit structure and brain function in mice and non‐human primates. These future tool development efforts may benefit from the synthesis of radioactive TMP analogs with improved pharmacokinetics and detectability.

Another important aspect of our work is that the technique is also applicable to non‐invasive studies of protein–protein interactions in normal and diseased neuronal networks. Aberrant formation of protein complexes has been implicated in several neurodegenerative disorders. For example, it is well‐established that progression of AD correlates with deposition of neurofibrillary tangles composed of aggregated microtubule‐binding protein Tau (Brunden *et al*, 2009; Morris *et al*, 2011). Although we and other groups have already developed PET probes to visualize mature Tau fibrils (Maruyama *et al*, 2013), there have been no sensitive readouts for the detection of soluble Tau oligomers that are thought to act as major neurotoxic species in AD (Kopeikina *et al*, 2012; Takashima, 2013). Our observations in mice expressing TRD‐NTF and TRD‐CTF suggest that [^18^F]FE‐TMP‐PET is suitable for monitoring early stages of Tau aggregation. Although further efforts would be necessary to determine the precise time‐course change in tau pathologies from oligomerization to mature fibril formation, this approach could help to elucidate the mechanisms and consequences of low‐order Tau self‐assembly in the pathogenesis of neurodegenerative tauopathies. Interestingly, Holmes *et al* recently established a fluorescence resonance energy transfer (FRET)‐based assay to monitor the seeding ability of Tau aggregates *in vitro* (Holmes *et al*, 2014). Since PCA is a simple alternative to FRET, bimodal imaging of ecDHFR‐based split biosensors could facilitate the development of versatile platforms to monitor the same effects in cell culture and *in vivo*.

Our study has demonstrated that ecDHFR/TMP‐based reporters are suitable for bimodal imaging in mice and non‐human primates. In genetically engineered animals, our technology allows a longitudinal whole brain assessment of cell type distribution, brain‐wide circuit reorganization, and pharmacological effects on the circuit integrity in living animals. For instance, a Cre‐ or Tetracycline‐inducible technology can be incorporated into animal genetics to achieve the expression and monitoring of ecDHFR reporter gene expression in specific cell types including neurons and glia (Daigle *et al*, 2018). In addition, as several types of IEG promoters such as E‐SARE and NPAS4 have been developed (Kawashima *et al*, 2013; Sun *et al*, 2020), the activity‐dependent induction of ecDHFR reporter expression in distinct neuronal subpopulations can also be visualized during the use of a variety of physiological stimuli and/or specific behavior tasks. Recently, a variety of non‐invasive neuroimaging modalities such as functional magnetic resonance imaging (fMRI) and functional near‐infrared spectroscopy has been utilized to assess the activated brain networks (Scarapicchia *et al*, 2017). Although these approaches rely on transient hemodynamic‐based measurement and lack information concerning cell type‐specific events, our reporter imaging can track a specific cell population over a long period in a specific brain network and will facilitate the construction of new assaying systems that elucidate the link between *in vivo* imaging and postmortem analysis of brain tissues, a process not feasible by fMRI.

Along with the recent advances in gene therapy and regenerative medicine, genetic reporter imaging could become a principal pillar of future biomedical applications. In a few studies, HSV1‐tk reporter imaging was indeed tested to track a tumor or infused cytolytic CD8^+^ T cells in human patients (Peñuelas *et al*, 2005; Yaghoubi *et al*, 2009), highlighting the feasibility and advantage of genetic reporter imaging for future clinical applications. At this stage, substantial, continuous effort will still be necessary to overcome the challenges in terms of safety, cost‐effectiveness, and ethics, although recent advances in the design of viral tools for non‐invasive gene delivery will make it feasible to eventually apply these reporters to biomedical PET imaging of human brains (Chan *et al*, 2017).

## Materials and Methods

### Reagents

The following chemicals and antibodies were commercially purchased and used in this study: TMP (Sigma, T7883); TMP‐Hexachlorofluorescein, TMP‐HEX (Active Motif, 34104); chicken anti‐GFP (AVES, GFP‐1020); mouse anti‐βActin (Sigma, A1978); mouse anti‐FLAG, clone M2 (Sigma, F1804); mouse anti‐HA (Covance, MMS‐101R); rabbit anti‐NeuN (Cell Signaling Tech, #12943S); rabbit anti‐HA (Cell Signaling Tech, #3724); rabbit anti‐cFos (Cell Signaling Tech, #2250); mouse anti‐GFAP (Millipore, MAB360); rabbit anti‐PDE10 (Protein Tech, 18078‐1‐AP); and chicken anti‐MAP2 (Abcam, AB5392).

### Animals

The animal studies here were maintained and handled according to the National Research Council’s Guide for the Care and Use of Laboratory Animals and our institutional guidelines. Experiments were performed and reported in accordance with the ARRIVE (Animal Research: Reporting *in vivo* Experiments) guidelines. All procedures involving animals and their care were approved by the Animal Ethics Committee of the National Institutes for Quantum and Radiological Science and Technology. We used postnatal day 0 or 2‐month‐old C57BL/6j mice (Japan SLC) for the genetic manipulation by Adeno Associate Virus (AAV)‐mediated gene delivery followed by two‐photon microscopy, PET scan, histology, and metabolite analysis. For *in vivo* injection of AAV into neonatal pups, animals were deeply anesthetized on ice, and 1.0 μl of purified AAV stocks (titer ranging from 0.5 × 10^9^ to 2.0 × 10^9^ viral genomes, Appendix Table S1) was injected directly into the side of a lateral cerebral ventricle via glass capillary. This technique was established as a relatively simple and fast method to manipulate gene expression in extensive brain regions with minimal long‐term damages (Chakrabarty *et al*, 2013; Kim *et al*, 2013), and we indeed observed that mice receiving AAV injection grew normally and exhibited uniform and enriched expression patterns of constructs in regions adjacent to ventricles, including the retrosplenial cortex, hippocampus, and some ventral parts of the brain without obvious neuroanatomical abnormalities at 2 months of age (Fig EV1B). We performed PET scans of these animals at 2 months of age. For AAV injection into adult mouse cortex, mice at 2–3 months of age were deeply anesthetized with 1.5–2.0% isoflurane and underwent a surgical procedure to attach cranial windows to one side of the somatosensory cortex (center was positioned 1.8 mm caudal and 2.5 mm lateral from bregma). 1.0–1.5 μl of AAV (titer ranging from 2.0 × 10^9^ to 8.0 × 10^9^ viral genomes; Appendix Table S1) was slowly injected via glass capillary into parenchyma positioned at a depth of 0.250–0.375 mm from the cortical surface during this procedure, and this model allowed us to perform side‐by‐side comparative imaging within a single animal (Fig EV1A). Alternatively, stereotactic injection was coordinated for AAV delivery into somatosensory cortex (stereotactic coordinates: anteroposterior, −1.5 mm; mediolateral, 2.0 mm; dorsoventral, 0.3 mm; relative to bregma) or hippocampus (stereotactic coordinates: anteroposterior, −2.5 mm; mediolateral, 2.0 mm; dorsoventral 1.5 mm; relative to bregma). We performed two‐photon microscopic and PET imaging more than 1 month after the surgical procedure. At this time point, intact BBB integrity was validated as radioactive signals of PET probes [^11^C]GF120918 and [^11^C]verapamil, well‐characterized substrates for P‐glycoprotein (Yamasaki *et al*, 2010), and there was no accumulation at the AAV injection site and neighboring areas (Appendix Fig S7). All mice were housed with littermates (2–5 mice depending on litter size) and maintained in a 12‐h light/dark cycle with ad libitum access to standard diet and water.

A 3.4‐year‐old male common marmoset was used as model for tracing the neuronal circuit in primate brain. The animal was born at the National Institutes for Quantum and Radiological Science and Technology and was housed as one of a pair, with one family member of the same sex, in a cage (0.66 (h) × 0.33 (w) × 0.6 (d) m). Environmental lighting was provided from 8:00 a.m. to 8:00 p.m. in this colony room. Room temperature was maintained at 24–30°C and relative humidity at 40–60%. Balanced marmoset food pellets (CMS‐1, CLEA Japan, Tokyo, Japan) were provided at sufficient amounts once a day, and water was available ad libitum in their home cage. For AAV injection, the marmoset was immobilized by intramuscular (i.m.) injection of ketamine (25 mg/kg) and xylazine (0.2 mg/kg) and intubated with an endotracheal tube. Anesthesia was maintained with isoflurane (1–3%, to effect). The marmoset then underwent a surgical procedure to open burr holes (∼5 mm diameter) for the injection needle. During surgery, CT images were captured and overlaid on magnetic resonance (MR) images using PMOD software (PMOD Technologies Ltd, Zurich, Switzerland) to obtain stereotaxic coordinates for spatial alignment between the injection needle and target location. AAV encoding ecDHFR‐EGFP expression cassette was slowly delivered (3 μl at 0.25 μl/min) into one side of the neocortex and striatum, and control AAV encoding kappa‐opioid receptor DREADD (KORD) was injected into the other, by means of a microsyringe (NanoFil, WPI, Sarasota, FL, USA) with a 33‐G beveled needle that was mounted into a motorized microinjector (Legato130, KD Scientific or UMP3T‐2, WPI, Sarasota, FL, USA). Other surgical procedures and viral injection were as described previously (Nagai *et al*, 2016).

### Radiosynthesis

[^11^C]TMP (trimethoprim) was radiosynthesized using the precursor compound 4‐((2,4‐diaminopyrimidine‐5‐yl)methyl)‐2,6‐dimethoxyphenol (Sundia). [^11^C]Methyl iodide was produced and transferred into 300 μl of N,N‐dimethylformamide (DMF) containing 1 mg of precursor and 7.2 μl of 0.5 N aqueous sodium hydroxide at room temperature. The reaction mixture was heated to 80°C and maintained for 5 min. [^18^F]fluoroethoxy‐TMP ([^18^F]FE‐TMP) was radiosynthesized using the precursor compound sodium 4‐((2,4‐diaminopyrimidine‐5‐yl)methyl)‐2,6‐dimethoxyphenolate (Sundia). [^18^F]Fluoroethyl bromide was produced and transferred into 250 μl of DMF containing 1.5 mg of precursor at room temperature. The reaction mixture was heated to 100°C and maintained for 10 min. After cooling the reaction vessel, the radioactive mixture was transferred into a reservoir for HPLC purification (Atlantis Prep T3, 10 x 150 mm; [^11^C]TMP: methanol/50 mM ammonium acetate = 40/60, 5.0 ml/min; [^18^F]FE‐TMP: methanol/50 mM ammonium acetate = 35/65, 4.5 ml/min; UV: 254 nm). The fraction corresponding to [^11^C]TMP or [^18^F]FE‐TMP was collected in a flask containing 100 μl of 25% ascorbic acid solution and Tween 80 and was evaporated to dryness under a vacuum. The residue was dissolved in 3 ml of saline (pH 7.4) to obtain [^11^C]TMP or [^18^F]FE‐TMP as an injectable solution. The final formulated products were radiochemically pure (95%) as determined by HPLC (Atlantis T3, 4.6 x 150 mm; methanol/50 mM ammonium acetate = 40/60, 1 ml/min; UV: 254 nm). Radiosynthesis of [^11^C]PBB3, a specific PET tracer for aggregated filamentous tau protein, was described previously (Maruyama *et al*, 2013). [^18^F]MNI659, a highly selective antagonist against phosphodiesterase 10A (PDE10A), was radiosynthesized by previously described methods (Mori *et al*, 2017). [^11^C]GF120918 and [^11^C]verapamil were radiosynthesized as described previously (Yamasaki *et al*, 2010). Ethyl [^18^F]fluoroacetate was prepared in a manner similar to that reported (Mori *et al*, 2009). Briefly, an anhydrous acetonitrile solution of ethyl 2‐(*p*‐toluenesulfonyloxy)acetate (3 mg/500 μl, Tokyo Chemical Industry Co., Ltd, Tokyo, Japan) was added to a reaction vessel containing dry [K/Kryptofix 2.2.2]^+18^F^–^, and the reaction mixture was left standing for 10 min at 105°C. The reaction solution was then applied to HPLC (column, XBridge C_18_ (Waters Corporation; Milford, MA, USA), 5 μm, 10 i.d × 250 mm; mobile phase; saline/ethanol=90/10; flow rate, 4 ml/min (t_R_: ca. 8.0 min)). The fraction containing ethyl [^18^F]fluoroacetate was collected in an injection vial. Total reaction time after bombardment was around 45 min. The yield was 47 ± 7.2% (*n* = 3) at the end of synthesis, with a radiochemical purity of more than 99%.

### Plasmid construction and virus preparation

pFUGW lentivirus shuttle plasmids with human Ubiquitin C promoter with customized multi‐cloning site were described previously (Shimojo *et al*, 2019). cDNAs of wild‐type ecDHFR (ecDHFR) and leucine zipper motif of Saccharomyces cerevisiae GCN4 (235‐281 aa) were synthesized as a gBlocks gene fragment (Integrated DNA Technologies). pBMN‐ecDHFR(DD)‐YFP was a kind gift from Dr. Thomas Wandless (Addgene plasmid #29325). pAAV‐RAM‐d2Venus plasmid was a kind gift from Drs. Tetsuo Yamamori and Masanari Ohtsuka (RIKEN). cDNA of Human Phosphodiesterase type 10 (hPDE10) was amplified by conventional nested PCR using human brain cDNA (Clontech). cDNA template encoding wild‐type and D674A mutant form of hPDE10 catalytic domain (CD, 449–789 aa), FKBP12‐rapamycin binding domain (FRB), and FK506 binding protein (FKBP) were synthesized as gBlock gene fragments. cDNAs encoding full‐length or repeat domain (RD) of human 2N4R tau protein were as described previously (Sahara *et al*, 2007). For protein complementation assay of ecDHFR reporter, N‐terminal fragment (NTF, 1–87 aa) or C‐terminal fragment (CTF, 88–159 aa) of ecDHFR was conjugated to ZIP or human Tau repeat domain (TRD) via linker sequence of GGGGSGGGGS. To generate plasmid vectors for expression of fluorescent or fusion proteins including EGFP, tdTomato, d2Venus, ecDHFR‐EGFP, ecDHFR‐d2Venus, DD‐EGFP, full‐length hPDE10 746 aa variant, ecDHFR‐hPDE10_CD‐EGFP, ecDHFR‐hPDE10(D674A)_CD‐EGFP, DD‐hPDE10_CD‐EGFP, FLAG‐ecDHFR‐HA, ZIP‐FLAG‐ecDHFR(NTF), ZIP‐ecDHFR(CTF)‐HA, TRD‐FLAG‐ecDHFR(NTF), and TRD‐ecDHFR(CTF)‐HA, each cDNA fragment was amplified by PCR and reassembled in the multi‐cloning site of pFUGW plasmid. Sequences of these plasmid DNAs were verified and directly used for transfection.

For production of recombinant lentiviruses, pFUGW shuttle vector, pVSVg, and pCMVΔ8.9 plasmids were co‐introduced into human embryonic kidney 293T cells using the FuGENE reagent (Promega). 48 h after transfection, secreted viruses in medium were collected, filtered through a 0.45‐µm filter, and infected to neuronal culture as described previously (Shimojo *et al*, 2019). Recombinant AAV plasmid with rat synapsin promoter (pAAV‐Syn) followed by customized multi‐cloning site, woodchuck posttranscriptional regulatory element (WPRE), and polyA signal flanked with ITRs was described previously (Pieraut *et al*, 2014). The cDNAs described above were subcloned directly into the multi‐cloning site of pAAV‐Syn plasmid from pFUGW‐based lentivirus plasmids. *In vitro* validation well confirmed the specific expression of EGFP in neurons from a locally injected AAV under control of the pan‐neuronal synapsin promoter (Fig EV1C). For activity‐dependent marking of neuronal ensemble, Syn promoter was substituted with RAM promoter. AAV encoding hM3Dq was described previously (Nagai *et al*, 2020). All other AAVs were prepared in large‐scale HEK293T cell cultures that were transfected with mixture plasmids of AAV encoding full‐length cDNAs and the serotype DJ packaging plasmids pHelper and pRC‐DJ with polyethylenimine (Polysciences). 48 h after transfection, cells were harvested and extracted, and then, AAV particles were purified using HiTrap heparin column (GE Healthcare) as described previously (McClure *et al*, 2011). The virus titer of purified AAV stocks was determined by AAVpro® Titration kit (for real‐time PCR) ver2 (TaKaRa).

### Cell culture

HEK293T cells were grown and maintained in DMEM (Invitrogen) supplemented with 10% fetal bovine serum (Sigma) and penicillin/streptomycin (Invitrogen) at 37°C in a 5% CO_2_ incubator. For all transfection experiments, cells were plated in 24‐well plates and transfected with 0.25 μg plasmid DNA and 1.25 μl FuGENE (Promega). Primary neuronal culture was prepared as described previously (Shimojo *et al*, 2019). Briefly, cortex and hippocampus from E17.5 mouse embryos were dissociated and plated onto poly‐D‐lysine (Sigma) coated 18‐mm glass coverslip at a density of 200,000–250,000 cells/cm^2^. Neurons were grown for 3–4 days in Neurobasal medium (Thermo Fisher) supplemented with 2% FBS (HyClone), GlutaMAX (Thermo Fisher), and B27 (Thermo Fisher), infected with recombinant lentivirus encoding d2Venus with RAM promoter, and further maintained in serum‐free medium. At DIV21, the medium was replaced by solution containing 10 mM HEPES‐NaOH, pH 7.4, 140 mM NaCl, 5 mM KCl, 0.8 mM MgCl_2_, 2 mM CaCl_2_, and 10 mM glucose, and neurons were stimulated by addition of 15 mM KCl or 100 μM picrotoxin (Tocris) for 6 h followed by fixation for immunocytochemical analysis as described previously (Shimojo *et al*, 2015a). Fluorescence images were then acquired by FV1000 laser scanning confocal microscopy (Olympus) with x60 (NA 1.35) oil immersion objectives.

### Live cell imaging and immunoblotting

To assess the stabilization of DD‐EGFP with TMP analogs, HEK293T cell culture medium was replaced by fresh medium with or without 10 μM TMP (Sigma) or FE‐TMP at 4 h after transfection and further incubated for additional 24 h. The next day, images of EGFP signal were captured, and cells were lysed in 1× SDS–PAGE sample buffer in Tris–HCl, pH 7.4, 150 mM NaCl, 2 mM EDTA for immunoblot analysis. The protein extracts were separated by either 10% or 10–20% Tris‐glycine gel, transferred to a nitrocellulose membrane, and subsequently probed with primary antibodies and HRP‐conjugated secondary antibodies (Jackson IR Lab). Chemiluminescent signal was elicited using ECL (PIERCE) reagents and detected by c‐Digit blot scanner (LI‐COR) or Amersham Imager 600 (GE Healthcare). Signal intensity was quantified by ImageJ software. For fluorescence time‐lapse imaging, transfected HEK293T cells on 24‐well plates were detached by trypsin and re‐plated onto poly‐D‐lysine (Sigma)‐coated 25‐mm glass coverslips. 24 h after plating, the coverslips were transferred to a custom‐built perfusion chamber mounted on the stage of an inverted Olympus IX83‐based fluorescence microscope controlled by Olympus Cell Sense Dimension software. Time‐series images were acquired at 0.2 Hz for 15 min at room temperature with an Orca‐Flash4.0 cMOS camera (Hamamatsu Photonics) using a ×20 objective lens (NA 0.45). At the beginning and ending time points, EGFP images were captured as references of transgene expression in each cell. Cells were perfused with HEPES‐buffered solution (10 mM HEPES‐NaOH, pH 7.4, 140 mM NaCl, 5 mM KCl, 0.8 mM MgCl_2_, 2 mM CaCl_2_, 10 mM glucose) at 2–3 ml/min. For pulse labeling of ecDHFR in living cells, cell permeable TMP‐hexachlorofluorescein (TMP‐HEX, active motif) was applied at a concentration of 100 nM for 2 min, followed by quick washout. To competitively block the labeling, DMSO (control) or 10‐μM TMP was applied to the imaging solution (final concentration of DMSO was 0.1% [v/v]). Acquired image stacks were analyzed with NIH ImageJ/Fiji. After brief image registration, regions of interest (ROI) were generated based on EGFP images by thresholding and were used for quantification. After extraction of the average fluorescence intensity in each ROI, normalized fluorescence change (ΔF/F_0_) was plotted and used for quantitative analysis. In this experimental condition, TMP‐HEX efficiently led to bright labeling of cells expressing the recombinant ecDHFR fused to EGFP but not control cells transfected with EGFP alone (Appendix Fig S8A). Fluorescence intensity reached a plateau within ∼15 min, but it remained at a background level when TMP‐HEX was mixed with an excess of unconjugated compound, supporting the saturability and specificity of TMP‐HEX binding (Appendix Fig S8B and C).

### Two‐photon microscope

*In vivo* labeling kinetics of ecDHFR reporter with TMP‐HEX in living animal brain was analyzed by two‐photon laser scanning microscopy (Tomita *et al*, 2005). We used 900‐nm wavelength pulse laser to elicit excitation of fluorophores. A single image plane was acquired at a fixed size (1,024 × 1,024 pixels square) with a depth of 0.2–0.4 mm from the surface. For simultaneous imaging of EGFP and TMP‐HEX, emission signals were separated by beam splitter and detected with band‐pass filters for green (525/50 nm) and red (610/75 nm), respectively. For kinetic analysis of DD‐EGFP stabilization by TMP administration, TMP (Sigma, T7883) dissolved in 10% DMSO / normal saline was prepared and immediately used for i.p. injection at a final concentration of 100 mg/kg. Time‐course changes in ecDHFR‐d2Venus fluorescence in response to air‐puff stimulation of whiskers or chemogenetic activation of hM3Dq with 10 mg/kg CNO i.p. injection were performed according to previously described methods (Takuwa *et al*, 2011; Ji *et al*, 2016).

### Animal PET

PET scans were conducted with a microPET Focus220 system (Siemens Medical Solutions USA, Malvern, USA) as described previously (Maeda *et al*, 2011). Briefly, animals were anesthetized with 1.0–3.0% isoflurane during all PET procedures. For injection of PET tracers, dental needles conjugated to polyethylene tubing were inserted into the tail vein of mice and SURFLO F&F (26G, Terumo Corp., Tokyo, Japan) was inserted into the marmoset saphenous vein. After intravenous bolus injection of radioactive ligands (mice: 42.6 ± 5.6 MBq for [^11^C]TMP, 40.7 ± 13.2 MBq for [^18^F]FE‐TMP, 37.0 ± 1.3 MBq for [^11^C]GF120918, 32.3 ± 6.1 MBq for [^11^C]verapamil, 25.5 ± 15.8 MBq for [^18^F]MNI659, or 19.2 ± 0.7 MBq for [^11^C]PBB3; marmoset: 112.7 MBq for [^18^F]FE‐TMP), dynamic emission scanning with 3D acquisition mode was conducted for 60 min ([^11^C]PBB3), 90 min ([^11^C]TMP, [^18^F]FE‐TMP [^11^C]GF120918, [^11^C]verapamil, and [^18^F]MNI659) or 180 min ([^18^F]FE‐TMP), respectively. Data were histogrammed into a 3D set of sonograms, and images were reconstructed by either maximum a posteriori method or filtered back‐projection using a Hanning filter cutoff at a Nyquist frequency (0.5 mm^–1^). All captured image data were subsequently analyzed using PMOD software (PMOD Technologies, Zurich, Switzerland). For spatial alignment of PET images, template MR images generated previously (Maeda *et al*, 2007) were used in this study. For quantification of radioactive signal during PET scan, VOIs were manually placed on the image fields. In the analysis of P0‐injected mouse brains, we placed VOIs in a region adjacent to the corpus callosum and hippocampus, which contain a maximum radioactive signal, and placed the reference in the striatal region with background signal. In the analysis of locally injected adult mouse brains, we manually set the same size of VOIs in the somatosensory‐motor cortical region on both ipsilateral and contralateral sides. Average radioactive signal in each VOI was calculated as standardized uptake value (SUV), representing the injected dose per cm^3^ volume compensated by body weight. PET analysis of monkey was performed as described previously (Nagai *et al*, 2016).

### Histology

Mice were deeply anesthetized and sacrificed by 4% PFA/PBS perfusion and fixation, and brains were subsequently dissected and further fixed in 4% PFA/PBS for 3 h at room temperature. For acquisition of whole brain imaging, fixed brains were imaged using M205FA fluorescence stereoscopic microscopy (Leica). After cryo‐protection in PBS containing 20% sucrose, brains were embedded and frozen in OCT compound (SaKuRa), and 20‐μm fixed frozen sections were prepared by cryostat. Alternatively, 100‐μm free‐floating coronal sections were sliced with a Leica VT1200S vibratome. All sections were blocked and permeabilized in PBS‐supplemented 4% BSA, 2% horse serum, and 0.25% Triton X‐100 at room temperature, incubated with primary antibodies, and then probed with secondary antibodies labeled Alexa488, 546, or 633. After extensive wash‐off of antibodies, nuclei were counter‐stained with DAPI (Invitrogen) and mounted on glass slides. For postmortem verification of reporter distribution in the marmoset brain, the monkey was anesthetized and sacrificed by 4% PFA/PBS perfusion and fixation. Dissected brain tissue was cryo‐protected in 30% sucrose/PBS followed by sectioning by cryostat. Slices of 20‐μm thickness were then mounted on glass slides, and fluorescence images were captured. For capturing low‐magnification images of entire brain slices, sections were imaged using BZ‐X710 fluorescence microscopy (Keyence) with ×10 objective (NA 0.45). For high‐magnification images to assess detailed cell type and/or structure, sections were analyzed by FV1000 laser scanning confocal microscopy (Olympus) with either ×40 (NA 1.30) or ×60 (NA 1.35) oil immersion objectives.

### *In vitro* binding assay

*In vitro* binding affinity of [^11^C]TMP ligand or [^18^F]FE‐TMP ligand against ecDHFR reporter and MNI659 against PDE10 variants in HEK293T cell lysate was assessed as described previously (Ono *et al*, 2017). To assay radioligand binding with homologous blockade, these homogenates (50 µg protein) were incubated with 5 nM [^11^C]TMP, 1 nM [^18^F]FE‐TMP, or 1 nM [^18^F]MNI659 in the presence of unlabeled ligands at varying concentrations ranging from 10^‐11^ to 10^‐6^ M in Tris–HCl buffer, pH 7.4, for 30 min at room temperature. These radioligand concentrations were determined in consideration of the radioactive half‐lives of ^11^C (∼20.4 min) and ^18^F (∼109.8 min). Samples were run in duplicates or triplicates. Inhibition constant (Ki) and percentage of displacement were determined by using non‐linear regression to fit a concentration‐binding plot to one‐site binding models derived from the Cheng‐Prusoff equation with Prism software (GraphPad), followed by F‐test for model selection. Dissociation constant (Kd) was calculated using this function: *K*_d_ = *K*_i_ = IC50 – [radioligand concentration].

For *in vitro* autoradiography, fixed frozen brain sections from mice expressing ecDHFR‐EGFP or tdTomato as control were pre‐incubated in 50 mM Tris–HCl buffer, pH 7.4, containing 5% ethanol at room temperature for 30 min, and incubated in 50 mM Tris–HCl buffer, pH 7.4, containing 5% ethanol and [^18^F]FE‐TMP (10 mCi/l) at room temperature for 30 min. Sections were then rinsed with ice‐cold Tris–HCl buffer containing 5% ethanol twice for 2 min and dipped into ice‐cold water for 10 sec. Sections labeled with [^18^F]FE‐TMP were subsequently dried with warm air and exposed to an imaging plate (BAS‐MS2025; Fuji Film). The imaging plate was scanned with a BAS‐5000 system (Fuji Film) to acquire autoradiograms.

### Metabolite analysis

To analyze the radioactive metabolite of [^18^F]FE‐TMP in plasma and brain of mice, blood was collected from the heart at 5, 15, 30, 60, and 90 min after intravenous injection of [^18^F]FE‐TMP (100–250 MBq) under anesthesia, followed by quick removal of the brain. Blood samples were centrifuged at 21,000 *g* for 3 min at 4°C. Plasma (200 μl) was transferred to a test tube containing acetonitrile (200 μl). The mixture was vortexed and centrifuged at 21,000 *g* for 3 min at 4°C for deproteinization. The brain was homogenized in ice‐cold saline. The homogenate (500 μl) was mixed with acetonitrile (500 μl) and centrifuged at 21,000 *g* for 3 min at 4°C for deproteinization. More than 93% of radioactivity in the tissues was extracted in the supernatants. Radioactive components in the supernatants were analyzed using an HPLC system equipped with a high‐sensitive positron detector (Ohyo Koken Kogyo Co. Ltd., Tokyo, Japan). The HPLC system and conditions were as follows: pump, PU‐2089 plus (Jasco); UV detector, UV‐2075 (Jasco); the high‐sensitive positron detector; column, Capcell Pak C_18_ AQ (Shiseido Co., Ltd, Tokyo, Japan), 5 μm, 10 i.d. × 250 mm; mobile phase, MeCN/0.1% phosphoric acid = 1/9; flow rate 4 ml/min; retention time, 12 min. Since TMP undergoes *O*‐demethylation and the preliminary metabolite analysis described above indicated that metabolite M1 was highly hydrophilic, M1 was expected to be [^18^F]fluoroacetic acid ([^18^F]FAcOH; see Fig EV4D) (Mori *et al*, 2009; Sellmyer *et al*, 2017a). Then, we compared the retention time of M1 with that of [^18^F]fluoroacetic acid, which was generated by the incubation of ethyl [^18^F]fluoroacetate in plasma of mouse at 37°C for 30 min (Mori *et al*, 2009), using a normal phase HPLC (column, Cosmosil HILIC (Nacalai Tesque Inc., Kyoto, Japan), 5 μm, 4.6 i.d. × 150 mm; mobile phase, MeCN/AcONH_4_ (10 mM, pH 7.0) = 1/1; flow rate, 1 ml/min) and a reverse phase HPLC (column, Capcell Pak C_18_ AQ (Shiseido Co., Ltd, Tokyo, Japan), 5 μm, 10 i.d. × 250 mm; mobile phase, MeCN/0.1% phosphoric acid (0–5 min, 9/1; 5–10 min, 9/1 to 1/1; 10–13 min, 1/1; 13–15 min, 1/1 to 9/1); flow rate, 4 ml/min).

### Statistics

Statistical significance of the data was analyzed with GraphPad Prism software (GraphPad Inc.). Unpaired Student’s *t*‐test or Mann–Whitney *U*‐test was used for comparison of two‐group data. For comparison of multiple groups, data were analyzed by one‐way ANOVA post hoc Dunnett test or Tukey–Kramer test. For analysis of time series data, statistical significance was determined by two‐way, repeated‐measures ANOVA followed by Bonferroni’s multiple comparison test.

## Author contributions

MS and MH conceived the study; MS generated expression vectors and recombinant viruses, and performed biochemical and histological analyses; MS, HT, MT, YS, YT, and NSu carried out *in vitro* and *in vivo* fluorescence imaging studies; MOn performed autoradiography and *in vitro* binding assays; JM, TMinamih, MOk, and TK analyzed TMP metabolites; M‐RZ and MF supervised chemical design, synthesis, and radioactive probing; MS, MT, CS, and YT performed PET and data analysis; KM, YN, and TMinamim performed PET and histological analysis of the primate. MH, AM, NSa, TS, and MS wrote the manuscript.

## Conflict of interest

The authors declare that they have no conflict of interest.

## Supporting information



Appendix

Expanded View Figures PDF

Source Data for Expanded View

Source Data for Figure 1

Source Data for Figure 2

Source Data for Figure 3

Source Data for Figure 4

Source Data for Figure 5

## Data Availability

This study contains no data deposited in external repositories.
