# Supplementary material for: A genetically targeted reporter for PET imaging of deep neuronal circuits in mammalian brains
Source: EMBO J. 2021 Oct 12;40(22):e107757. doi: 10.15252/embj.2021107757 (PMC8591537; doi:10.15252/embj.2021107757)
Supplement: Supplementary file 1 — Appendix [file EMBJ-40-e107757-s005.pdf]

Appendix

A genetically targeted reporter for PET imaging of deep neuronal circuits in mammalian brains

Authors

Masafumi Shimojo[1, *], Maiko Ono[1], Hiroyuki Takuwa[1], Koki Mimura[1], Yuji Nagai[1], Masayuki Fujinaga[2], Tatsuya Kikuchi[2], Maki Okada[2], Chie Seki[1], Masaki Tokunaga[1], Jun Maeda[1], Yuhei Takado[1], Manami Takahashi[1], Takeharu Minamihisamatsu[1], Ming-Rong Zhang[2], Yutaka Tomita[3], Norihiro Suzuki[3], Anton Maximov[4], Tetsuya Suhara[1], Takafumi Minamimoto[1], Naruhiko Sahara[1], Makoto Higuchi[1, *]

Table of Contents

Appendix Figure S1 and S1 legend....................page 3
Appendix Figure S2 and S2 legend....................page 4
Appendix Figure S3 and S3 legend....................page 5
Appendix Figure S4 ....page 6
Appendix Figure S4 legend................................page 7
Appendix Figure S5 and S5 legend....................page 8
Appendix Figure S6 and S6 legend....................page 9
Appendix Figure S7 ....page 10
Appendix Figure S7 legend..............................page 11
Appendix Figure S8 ....page 12
Appendix Figure S8 legend..............................page 13
Appendix Figure S9 and S9 legend..................page 14
Appendix Table S1 ....page 15

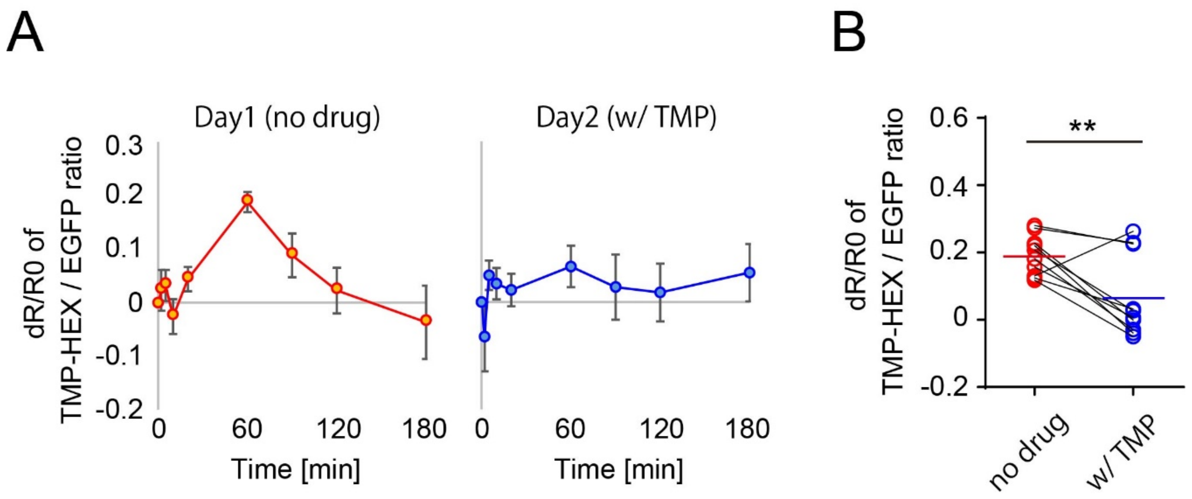

Appendix Figure S1. Longitudinal kinetics of TMP-HEX labeling in the same neurons

Extended two-photon microscopic analysis of an awake mouse brain was coordinated after TMP-HEX i.v. injection with or without a saturated amount of conventional TMP peripheral administration. All imaging experiments were performed by PFM450 microscope (Thorlabs, Tokyo, Japan). Laser pulses were generated by Mai Tai HP laser (SpectrPhysics, Santa Clara, CA). The excitation wavelength was set to 900 nm for simultaneous imaging of EGFP and TMP-HEX, and emission signals were separated by a beam splitter and detected with band-pass filters for green (525/50nm) and red (607/70nm), respectively.

A) On day 1, the kinetics of TMP-HEX labeling on neuronal cells expressing ecDHFR were monitored for a 3-hour imaging session. On day 2, the mouse received 100mg/kg TMP i.p. administration 45 min before conducting the same experimental session. Ratios of TMP-HEX fluorescence intensity relative to EGFP in 10 cells were quantified and plotted as Mean ± S.E.M.

B) Averaged ratios of TMP-HEX fluorescence intensity relative to EGFP in individual cells at 60 min. Data represent Mean (horizontal bar) and values from individual cells (dots) for each condition. $t(9) = 3.421$; $^{**}p < 0.01$ (paired t-test).

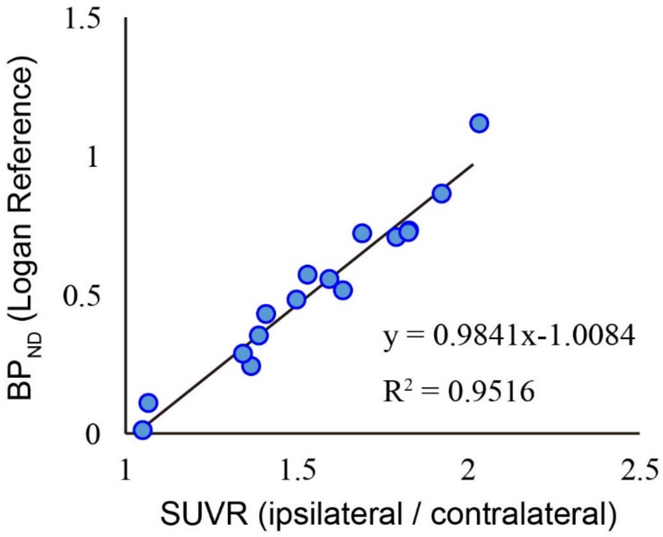

Appendix Figure S2. Scatter plot analysis between SUVR vs BP$_{ND}$ in PET imaging with [^{18}F]FE-TMP

SUVR (SUV ratio, averaged between 60-90 min scan) of ipsilateral/contralateral signals in the neocortex region of ecDHFR-EGFP-expressing individual animals shown in Fig 1H (n=16) were re-plotted with BP$_{ND}$ determined by Logan reference models. Each dot represents the value of an individual animal. Correlation with R^2 values was determined by linear regression analysis.

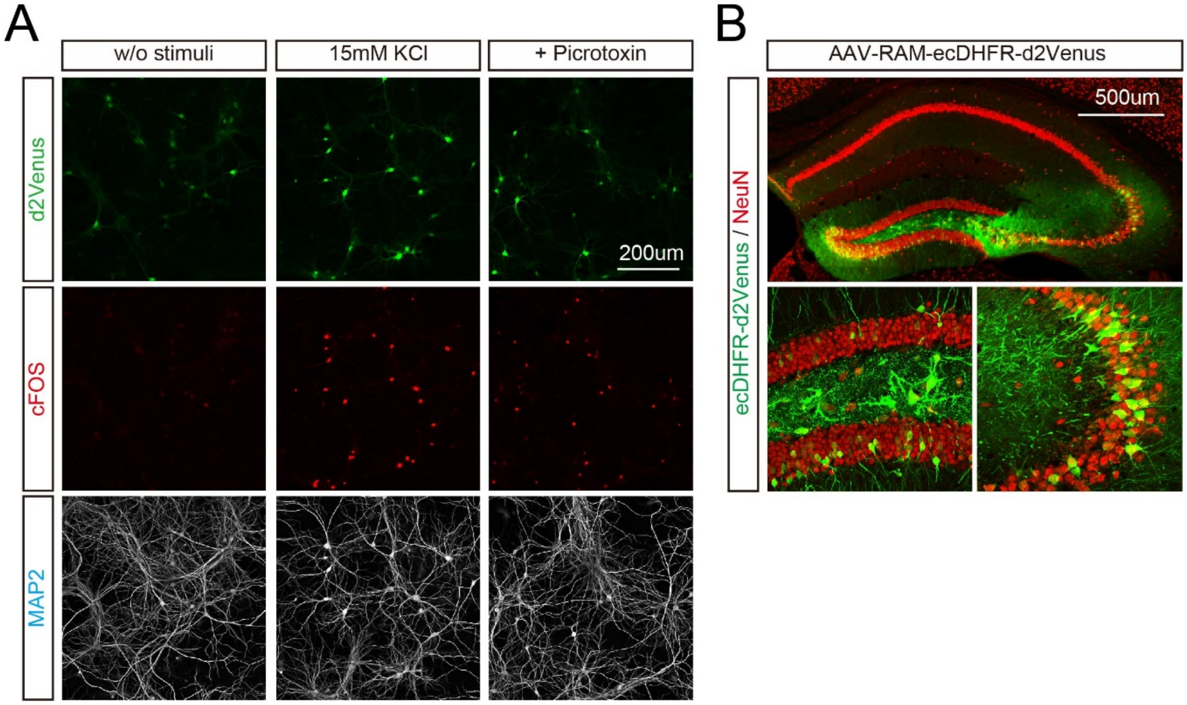

Appendix Figure S3. Efficient labeling of activated neurons with RAM promoter *in vitro* and *in vivo*

A) DIV21 neuronal culture expressing d2Venus under control of RAM promoter was incubated with 15 mM KCl (middle) or picrotoxin (right) for 6 h. Fixed neurons were immunostained with anti-cFOS and anti-MAP2 antibodies and analyzed by confocal fluorescence microscopy. Representative images demonstrate enhanced signals of d2Venus fluorescence and cFos immunoreactivity in neurons after chemical stimuli.

B) AAV-RAM-ecDHFR-d2Venus and AAV-CAG-M3-DREADD were co-injected into one side of the hippocampus. The animal was sacrificed one week after 0.3 mg/kg CNO i.p. injection for PET analysis, and distribution of ecDHFR-d2Venus in brain slices was assessed by confocal microscopy.

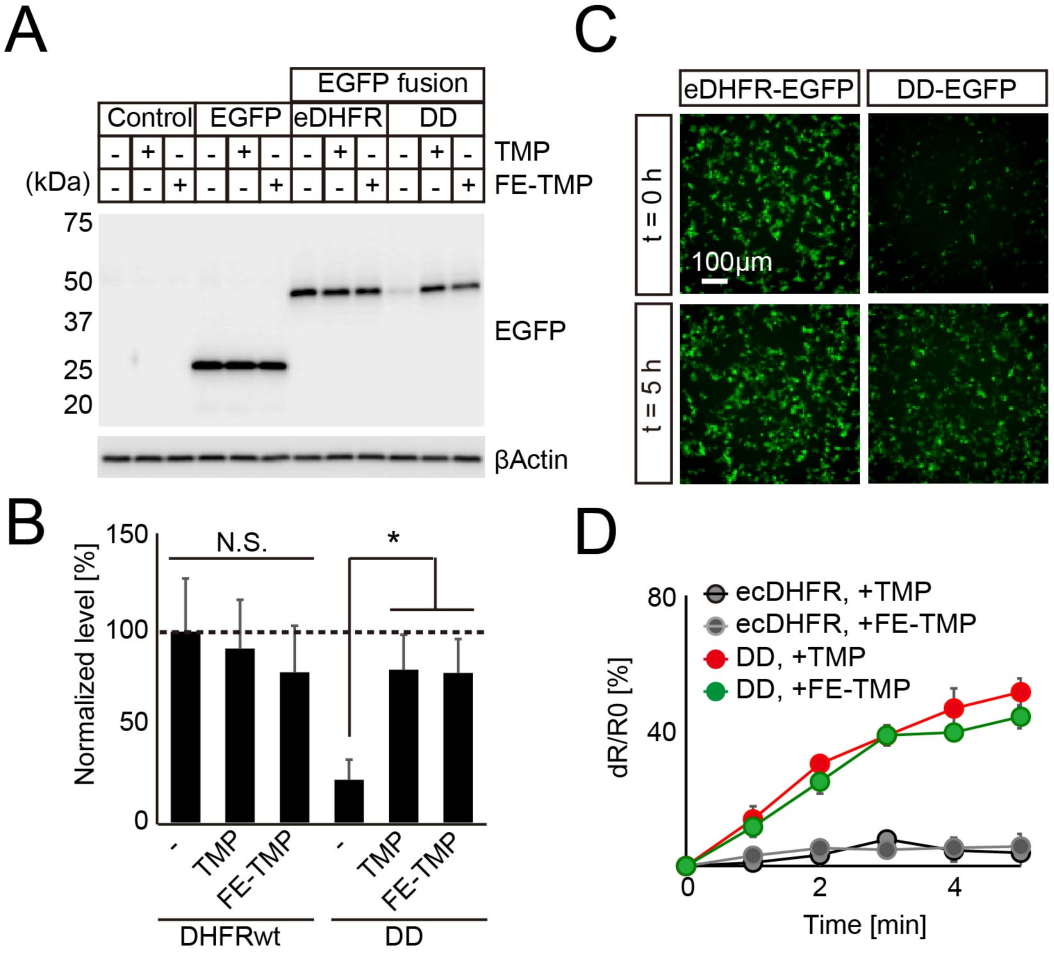
A
EGFP fusion
Control
EGFP
eDHFR
DD
TMP
(kDa)
FE-TMP
75
50
37
25
20
EGFP
βActin
B
150
100
50
0
Normalized level [%]
N.S.
*
-
TMP
FE-TMP
DHFRwt
DD
C
eDHFR-EGFP
DD-EGFP
t = 0 h
t = 5 h
100μm
D
80
40
dR/R0 [%]
ecDHFR, +TMP
ecDHFR, +FE-TMP
DD, +TMP
DD, +FE-TMP
0
2
4
Time [min]

Appendix Figure S4. Stabilization of destabilized ecDHFR mutant by TMP analogs

A-D) Cultured HEK293 cells expressing ecDHFR-EGFP or DD-EGFP were incubated with 10 μM TMP or FE-TMP and analyzed by immunoblotting and fluorescence microscopy.

A) Protein stabilization of wild-type ecDHFR and DD fused to EGFP were analyzed by immunoblotting with anti-GFP chicken IgY. For loading control, protein levels of β-actin were assessed with specific antibodies. Note that incubation with TMP and FE-TMP did not affect protein levels of wild-type ecDHFR-EGFP but remarkably induced stabilization of DD during 24-h observation.

B) Quantitative analysis of immunoblot data. Data from three independent experiments are shown as Mean ± SD. $F(5, 12) = 4.746$; $^{*}p < 0.05$ (one-way ANOVA followed by Dunnett post-hoc test).

C) Time-course analysis of DD-EGFP stabilization by TMP in HEK293T cells. Plasmid DNA vectors encoding either wild-type ecDHFR-EGFP or DD-EGFP were transfected into cells with control vectors coding mCherry. From 30 h after transfection, protein stabilization of DD-EGFP by 10 μM ligand addition to the culture medium was monitored hourly. Note that only DD-EGFP fluorescence signal was specifically upregulated by TMP addition.

D) Ratiometric quantitative analysis of fluorescence signals of EGFP relative to mCherry fluorescence. Changes of EGFP-to-mCherry ratio at each time point is shown as % of the ratio at $t = 0$. Data from three independent experiments are displayed as Mean ± SD. Note that TMP and FE-TMP possess similar ability to stabilize DD proteins in this condition.

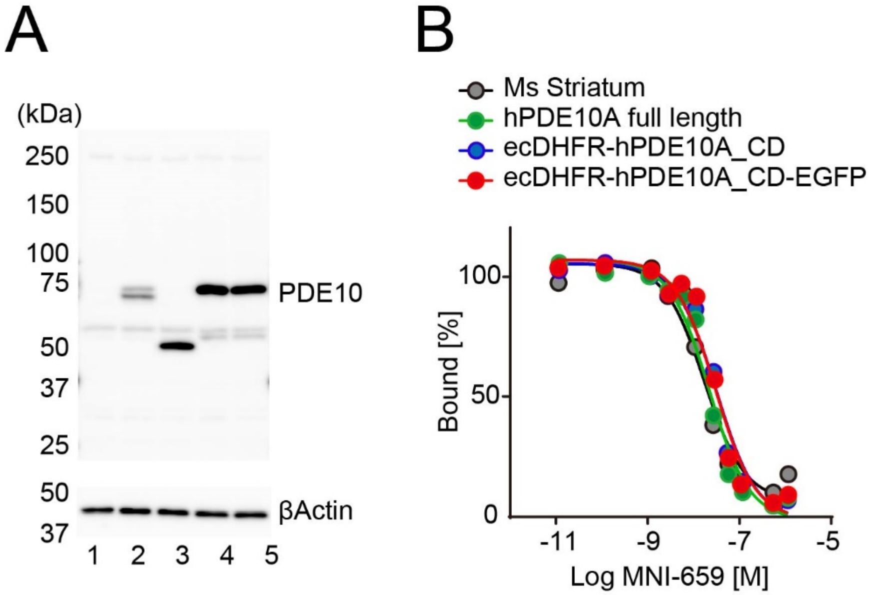

Appendix Figure S5. Analyses of ecDHFR reporters fused with cyclic phosphodiesterase (PDE10A)

A) Expression of indicated constructs in cultures of HEK293 cells. Protein extracts were analyzed by immunoblotting with antibodies against PDE10 or β-Actin (as loading control). Lane 1: vector control, 2: full-length PDE10A, 3: ecDHFR-PDE10A_CD, 4: ecDHFR-PDE10A_CD-EGFP, 5: ecDHFR-PDE10A(D674A)_CD-EGFP.

B) Binding of [^{18}F]MNI659 to various recombinant PDE10A reporters was assessed by competition assay with various concentrations of non-labeled MNI659 compound. Mouse striatum homogenate, which contains endogenous PDE10A, was used as positive control. Apparent Bmax (Maximum Binding) was set at 100%.

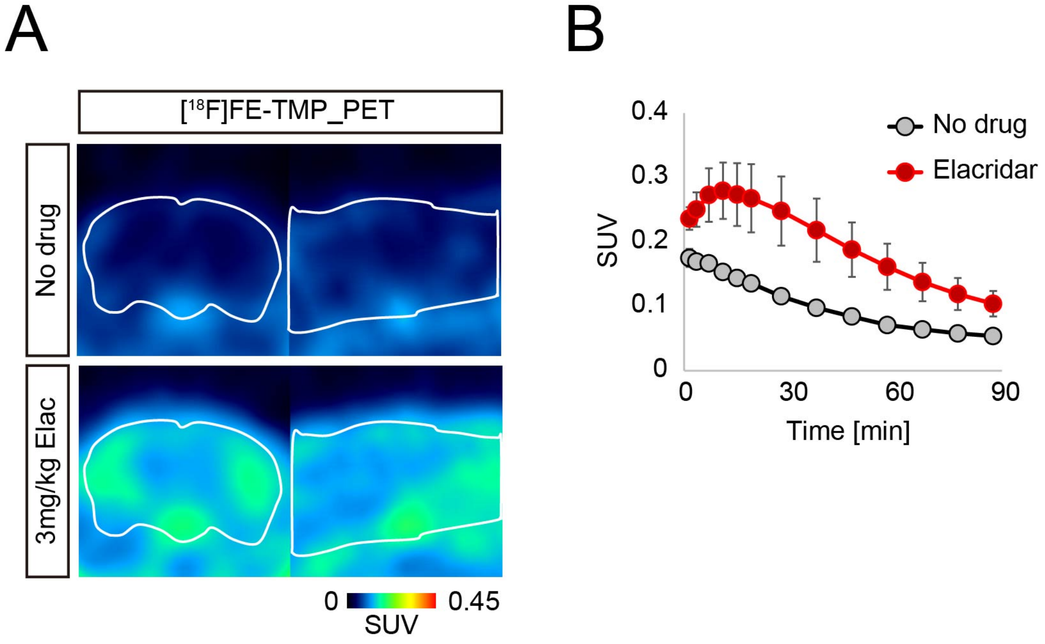

Appendix Figure S6. [^{18}F]FE-TMP PET imaging with P-glycoprotein inhibition

[^{18}F]FE-TMP PET scan of C57BL/6j mice was conducted before and 45 min after i.v. injection of 3mg/kg Elacridar, a selective inhibitor of the multidrug efflux transporter P-glycoprotein (Pgp).

A) Averaged PET images (coronal and sagittal slices from left) of dynamic scan data at 60-90 min after i.v. injection of [^{18}F]FE-TMP are shown. White lines denote outlines of brain. Note accumulation of radioactive signals by Elacridar pre-administration.

B) Time-radioactivity curves of cortical radioactive signals during 0-90 min dynamic scans in individual animals w/ or w/o 3mg/kg Elacridar treatment (n = 5 each). Data were plotted as Mean ± S.D.

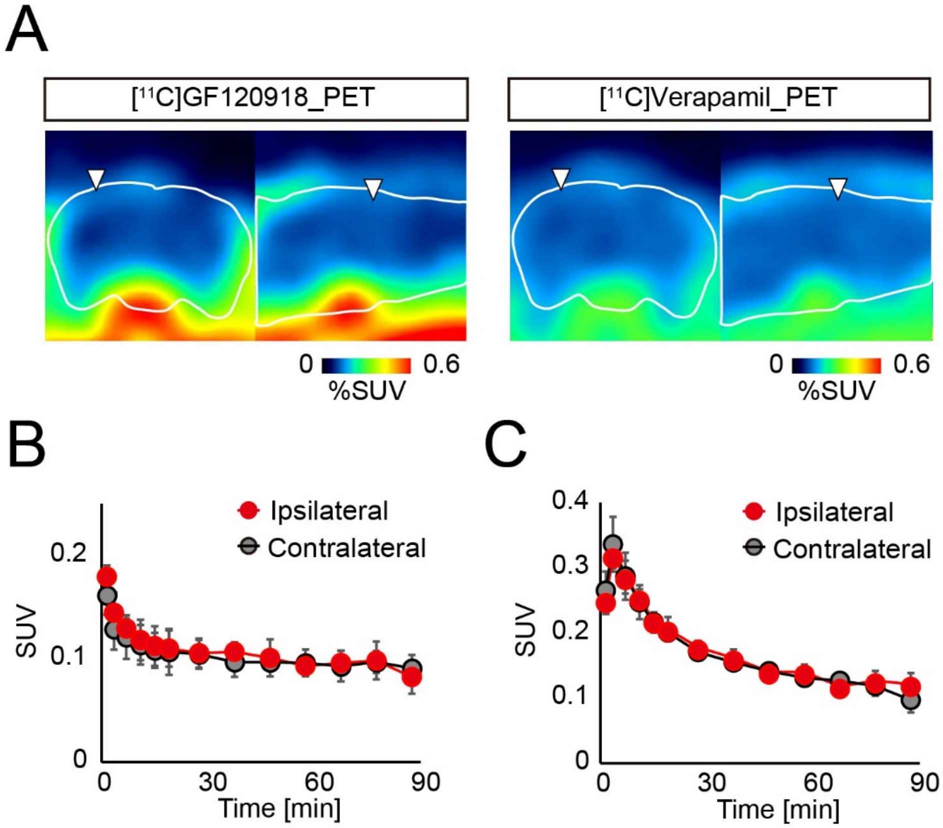
A
[11C]GF120918_PET
[11C]Verapamil_PET
0
0.6
%SUV
0
0.6
%SUV
B
Ipsilateral
Contralateral
SUV
0.2
0.1
0
0
30
60
90
Time [min]
C
Ipsilateral
Contralateral
SUV
0.4
0.3
0.2
0.1
0
0
30
60
90
Time [min]

Appendix Figure S7. BBB integrity in mouse brains after local AAV delivery

BBB integrity in brains of AAV-injected mice was assessed by PET scans with radiolabeled substrates for p-glycoprotein [^{11}C]GF120918 and [^{11}C]verapamil (1 month post-injection).

A) Representative PET images (coronal and sagittal sections from left) generated by averaging dynamic scan data at 0 – 90 min after i.v. injection of [^{11}C]GF120918 (left) and [^{11}C]verapamil (right). White lines mark whole brain area as determined by MRI. Arrowheads indicate areas of AAV delivery. Scale bar represents SUV.

B) Time-radioactivity curves in ipsilateral (red symbols) and contralateral (black symbols) cortices after administration of [^{11}C]GF120918. Note that AAV inoculation did not induce alteration of radiotracer retention in ipsilateral versus contralateral cortex. Data are Mean ± SD (n = 5 mice).

C) Time-radioactivity curves in ipsilateral (red symbols) and contralateral (black symbols) cortices after administration of [^{11}C]verapamil. Data are Mean ± SD (n = 4 mice).

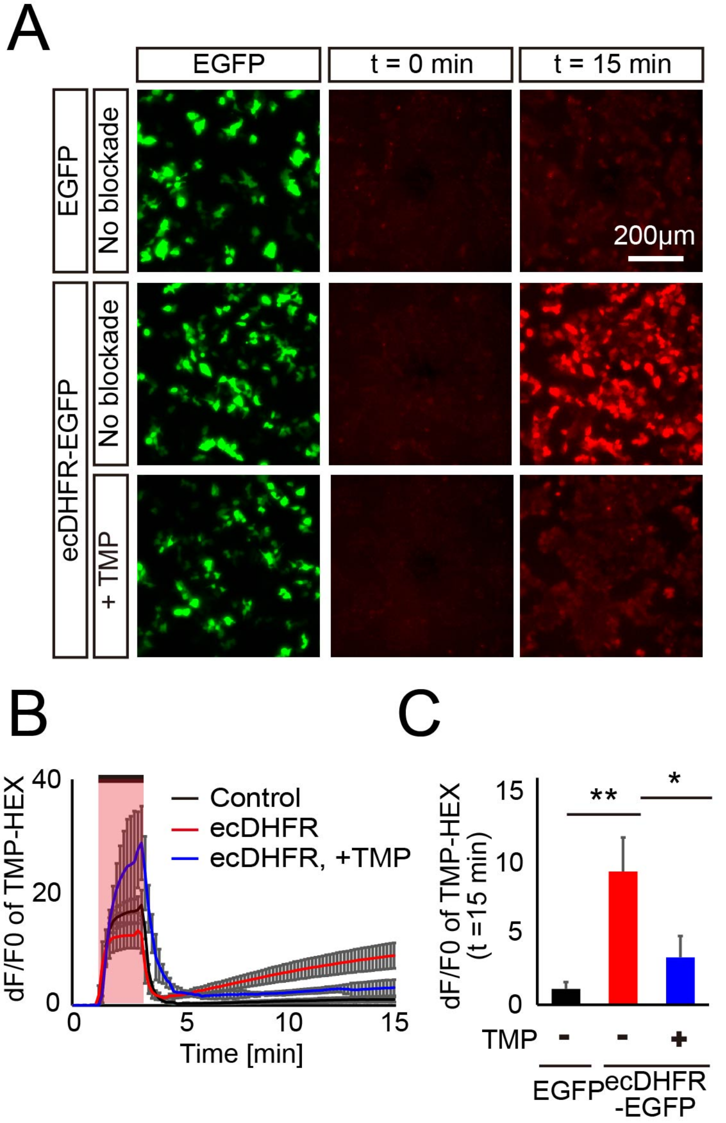
A
EGFP
t = 0 min
t = 15 min
EGFP
No blockade
200μm
ecDHFR-EGFP
No blockade
+ TMP
B
dF/F0 of TMP-HEX
40
20
0
0
5
10
15
Time [min]
Control
ecDHFR
ecDHFR, +TMP
C
dF/F0 of TMP-HEX (t =15 min)
15
10
5
0
**
*
TMP
-
-
+
EGFP
ecDHFR -EGFP

Appendix Figure S8. ***In vitro*** **fluorescent labeling of ecDHFR with TMP-HEX**

Cultured HEK293T cells expressing EGFP or ecDHFR-EGFP were imaged in time-lapse mode before, during, and after transient perfusion of 200 nM TMP-Hexachlorofluorescein (TMP-HEX). The ligand was applied for 2 min followed by wash-out.

A) Representative images show selective retention of TMP-HEX (red) in cells carrying ecDHFR-EGFP (middle). Note that labeling is strongly suppressed in the presence of excess conventional TMP (10 μM, lower).

B) TMP-HEX fluorescence intensities during time-lapse image sessions, plotted as dF/F0 ratios. The bar marks the 2-min window of TMP-HEX perfusion. Data from three independent experiments are indicated as Mean ± SD. At 5-15 min, $F(2,6) = 23.08$; $p < 0.01$ (two-way, repeated measure ANOVA).

C) dF/F0 ratios of TMP-HEX fluorescence intensities at endpoint of the assay (t = 15 min). Data from three independent experiments are indicated as Mean ± SD. $F(2,6) = 19.80$; $*p < 0.05$, $**p < 0.01$ (one-way ANOVA followed by Tukey-Kramer post-hoc test).

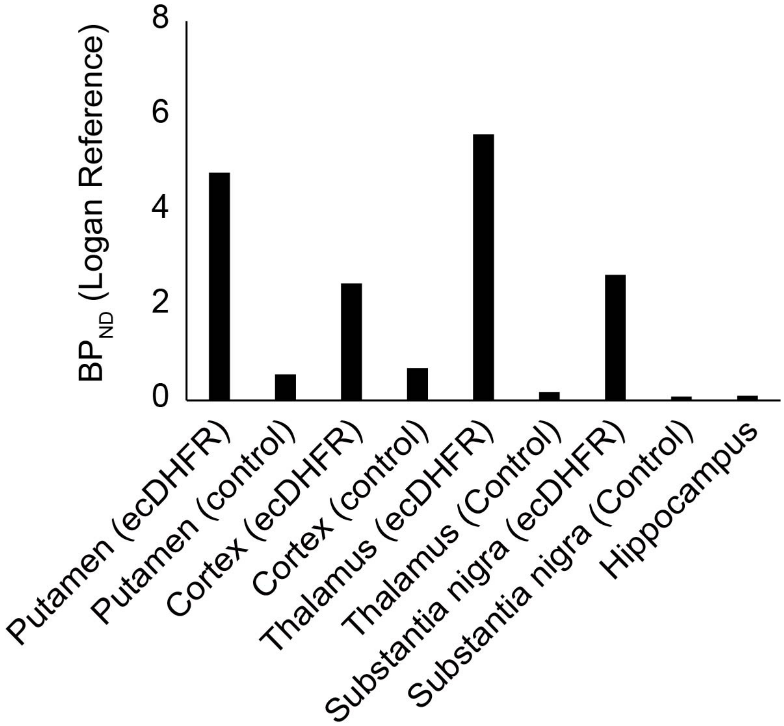

Appendix Figure S9. BP$_{ND}$ in PET imaging with [^{18}F]FE-TMP

Binding potential (BP$_{ND}$) with [^{18}F]FE-TMP PET imaging of ecDHFR-EGFP-expressing common marmoset was determined by Logan reference models with cerebellum reference.

AAV ID	Serotype	Injected titer [vg]	Promoter	5'UTR	3'UTR	Related Figures	Application
AAV-Syn-mCherry	DJ	2.3×10^{9}	rat synapsin	-	WPRE, polyA	Fig 1	adult mouse, cortex
AAV-Syn-ecDHFR-EGFP	DJ	2.9×10^{9}	rat synapsin	-	WPRE, polyA	Fig 1	adult mouse, cortex
AAV-RAM-ecDHFR-d2Venus	DJ	3.9×10^{9}	RAM/cFos	sythetic intron	WPRE, polyA	Fig 2	adult mouse, cortex and hippocampus
AAV-Syn-DD-EGFP	DJ	2.5×10^{9}	rat synapsin	-	WPRE, polyA	Fig 4	adult mouse, cortex
AAV-Syn-ecDHFR-hPDE10_CD-EGFP	DJ	1.9×10^{9}	rat synapsin	-	WPRE, polyA	Fig 4	adult mouse, cortex
AAV-Syn-DD-hPDE10_CD-EGFP	DJ	2.0×10^{9}	rat synapsin	-	WPRE, polyA	Fig 4	adult mouse, cortex
AAV-Syn-ZIP-ecDHFR(CTF)-HA	DJ	2.0×10^{9}	rat synapsin	-	WPRE, polyA	Fig 5	adult mouse, cortex
AAV-Syn-hTauRD-FLAG-ecDHFR(NTF)	DJ	2.3×10^{9}	rat synapsin	-	WPRE, polyA	Fig 5, Fig EV5	adult mouse, cortex
AAV-Syn-hTauRD-ecDHFR(CTF)-HA	DJ	3.4×10^{9}	rat synapsin	-	WPRE, polyA	Fig 5, Fig EV5	adult mouse, cortex
AAV-Syn-EGFP	DJ	2.4×10^{9}	rat synapsin	-	WPRE, polyA	Fig EV1	adult mouse, cortex
AAV-Syn-EGFP	DJ	0.8×10^{9}	rat synapsin	-	WPRE, polyA	Fig EV1	neonatal mouse, lateral ventricle
AAV-Syn-tdTomato	DJ	1.0×10^{9}	rat synapsin	-	WPRE, polyA	Fig EV2, Fig EV3	neonatal mouse, lateral ventricle
AAV-Syn-ecDHFR-EGFP	DJ	1.9×10^{9}	rat synapsin	-	WPRE, polyA	Fig EV2, Fig EV3	neonatal mouse, lateral ventricle
AAV-Syn-ecDHFR-EGFP, for marmoset	DJ	7.2×10^{9}	rat synapsin	-	WPRE, polyA	Fig 3	marmoset
AAV-hSynI-KORD-IRES2-AcGFP	1	2.1×10^{11}	human synapsin	-	WPRE, polyA	Fig 3	marmoset

Appendix Table S1. Summary of Adeno associated Virus vectors in this study
