# Supplementary material for: A genetically targeted reporter for PET imaging of deep neuronal circuits in mammalian brains
Source: EMBO J. 2021 Oct 12;40(22):e107757. doi: 10.15252/embj.2021107757 (PMC8591537; doi:10.15252/embj.2021107757)
Supplement: Supplementary file 2 — Expanded View Figures PDF [file EMBJ-40-e107757-s009.pdf]

Expanded View Figures

Figure EV1. Comparative assessment of transgene expression by AAV injection into rodent brain with two different protocols.

A Schematic diagram of AAV injection into one side of somatosensory cortex with cranial window in adult mouse brain. EGFP expression was introduced under control of synapsin promoter. Spatial distribution of EGFP was analyzed in whole brain (upper) and coronal brain slices (lower). Note that EGFP expression was highly restricted to injection site in this protocol. Arrowhead indicates AAV injection site.

B Schematic diagram of AAV injection into one side of lateral cerebral ventricle at postnatal day 0. Spatial distribution of EGFP was analyzed in whole brain (upper) and coronal slices (lower). Note that high-level expression of EGFP is observed in circumferentially arranged area of the ventricle system including corpus callosum, hippocampus, and parietal cortex.

C Neuron-specific expression of EGFP under control of synapsin promoter is verified by immunostaining using anti-GFAP antibody. Representative images of neurons (green) and astrocytes (red) with DAPI staining (blue) in hippocampal CA1 captured by confocal microscopy are displayed.

D AAVs encoding ecDHFR-EGFP were unilaterally injected into somatosensory cortex. Enriched neuronal expression of ecDHFR-EGFP at injection site was assessed by fluorescence imaging of brain slices. Note arrowhead indicates that ecDHFR-EGFP was also weakly distributed to axonal fibers derived from neurons of injection site.

▶

© 2021 The Authors

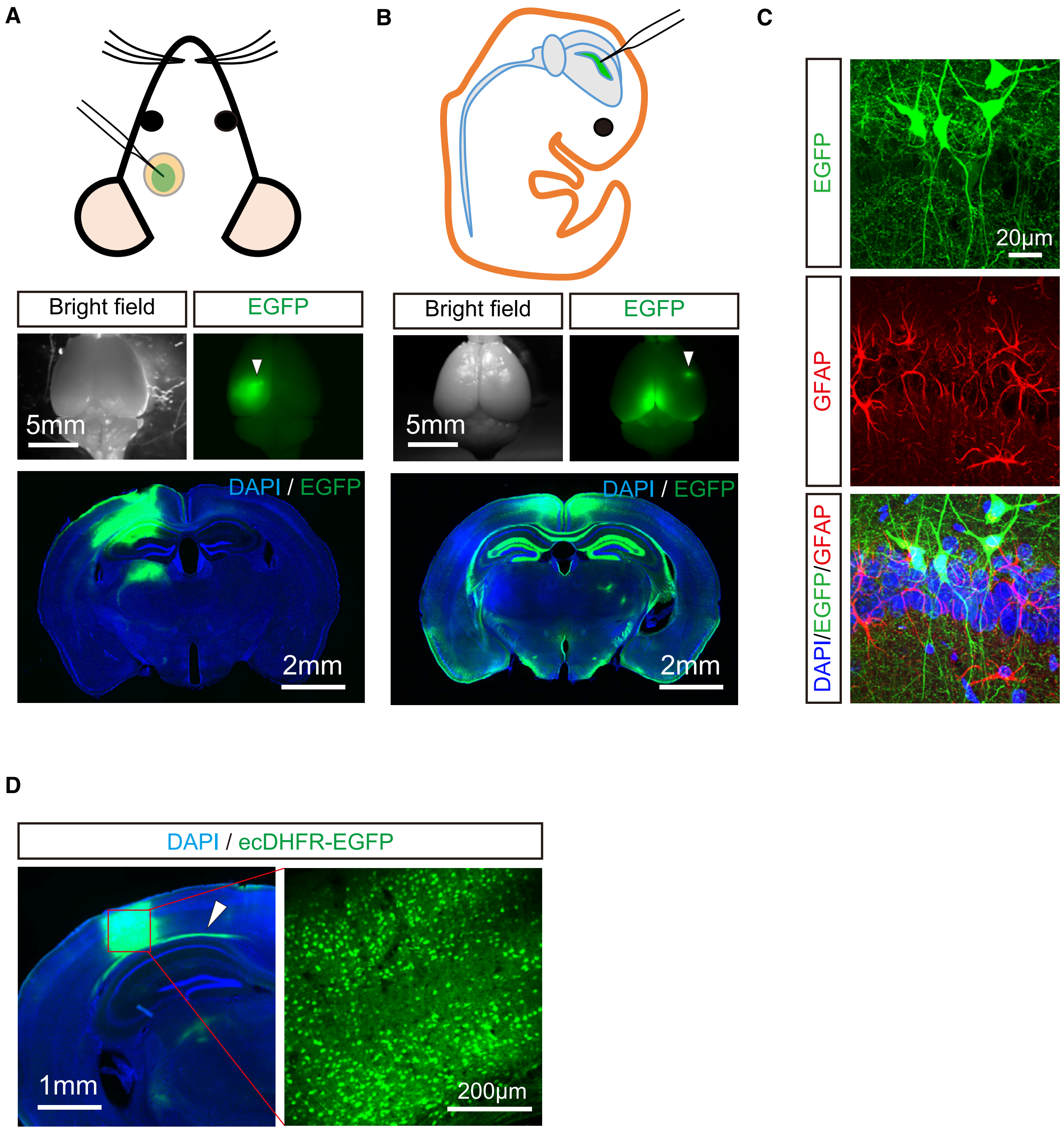

Figure EV1.

 © 2021 The Authors

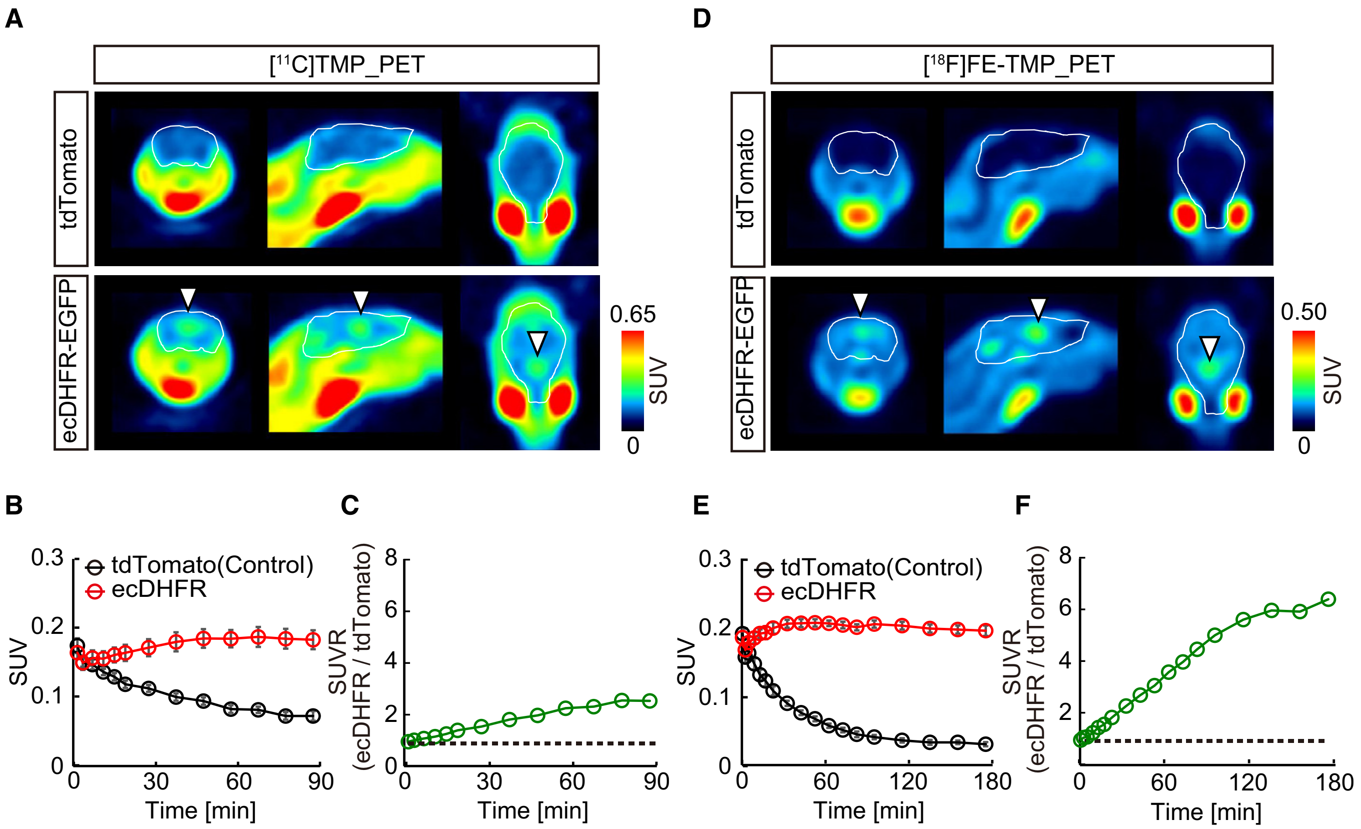

Figure EV2. PET imaging of ecDHFR in mouse brain with radioactive TMP analogs.

Mice expressing ecDHFR-EGFP or tdTomato (as control) from AAVs in forebrain were subjected to PET scans after peripheral administration of radioactive TMP analogs [^{11}C]TMP and [^{18}F]FE-TMP (40 MBq/mouse).

A Representative PET images (coronal, sagittal, and horizontal sections from left) generated by averaging dynamic scan data at 0–90 min after i.v. injection of [^{11}C]TMP. Arrowheads indicate areas of accumulation of radioactive ligand in animals carrying ecDHFR-EGFP (lower). White lines mark whole brain area as determined by MRI.

B [^{11}C]TMP labeling kinetics. Volumes of interest (VOI) of fixed sizes were manually placed on paraventricular region exhibiting high-level radioactive signals. Data from control (n = 7) and ecDHFR-EGFP-expressing mice (n = 7) were plotted as mean ± SEM. $F(1, 12) = 22.05$; $P < 0.01$ (two-way ANOVA).

C Ratios of averaged [^{11}C]TMP radioactive signals in ecDHFR versus control brains.

D Representative PET images (coronal, sagittal, and horizontal sections from left) generated by averaging dynamic scan data at 0–180 min after i.v. injection of [^{18}F]FE-TMP. Arrowheads indicate areas of accumulation of radioactive ligand in animals carrying ecDHFR-EGFP (lower). White lines mark whole brain.

E [^{18}F]FE-TMP labeling kinetics. VOI analysis was performed as described in panel c. Data from control (n = 6) and ecDHFR-EGFP-expressing mice (n = 6) were plotted as mean ± SEM. $F(1, 10) = 326.1$; $P < 0.01$ (two-way ANOVA).

F Ratios of averaged [^{18}F]FE-TMP radioactive signals in ecDHFR versus control brains.

Source data are available online for this figure.

© 2021 The Authors

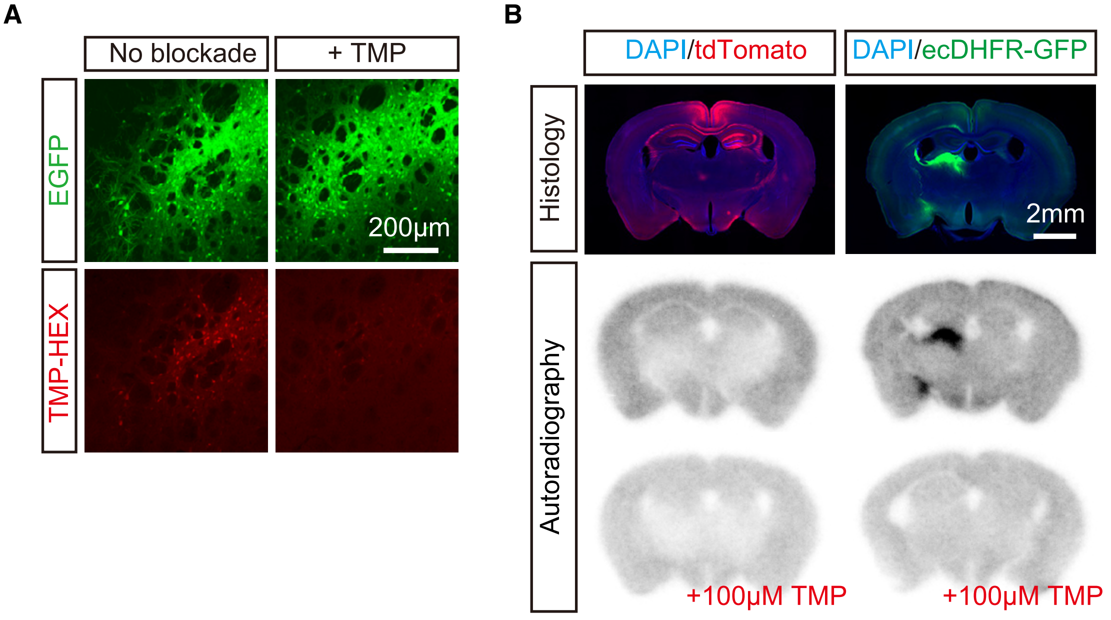

Figure EV3. ***In vitro* validation of TMP-HEX and [^{18}F]FE-TMP for ecDHFR.**

A Representative images of striatal neurons expressing high-level ecDHFR-EGFP in fixed brain slices labeled with TMP-HEX. Note that incubation with excess amount of non-labeled TMP markedly inhibits fluorescence labeling.

B *In vitro* autoradiography of mouse brain sections with [^{18}F]FE-TMP. Expression of transgenes in samples collected from mice treated with control and ecDHFR-EGFP vectors is fluorescently visualized with DAPI counterstaining (upper). Representative images of *in vitro* autoradiography using [^{18}F]FE-TMP demonstrate that this radioligand specifically labels brain areas overexpressing ecDHFR-EGFP but not tdTomato (middle), and that this radioligand binding to putative ecDHFR is profoundly blocked by an excess amount of non-labeled TMP (lower).

 © 2021 The Authors

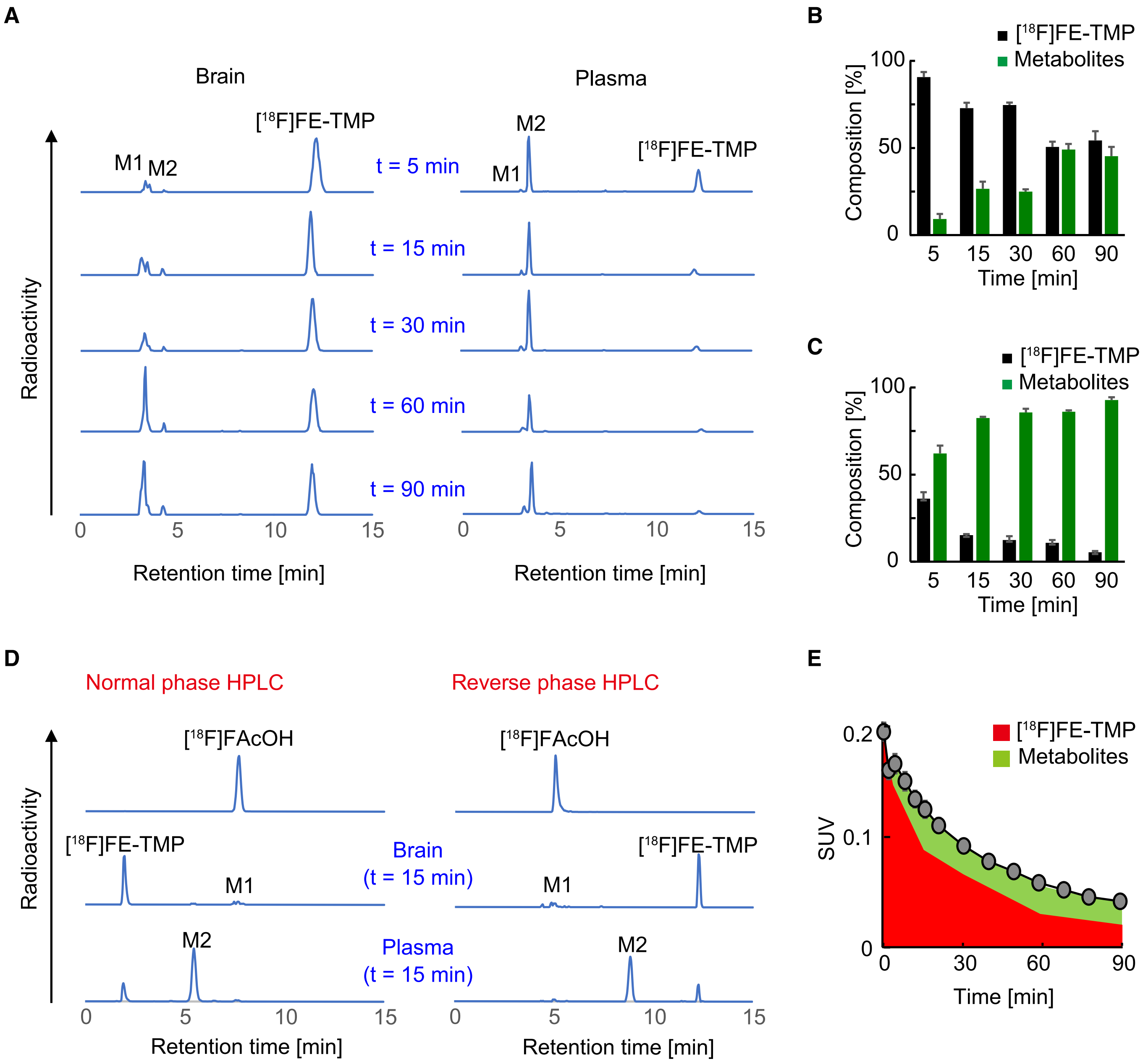

Figure EV4. Characterization of [18F]FE-TMP radioactive metabolites.

A Representative reverse phase radio-HPLC charts showing [18F]FE-TMP and its radiometabolites, M1 and M2, in brain (left) and plasma (right) of mice at 5, 15, 30, 60, and 90 min after i.v. injection of [18F]FE-TMP. M1 and M2 are detected as major radiometabolites in brain and plasma, respectively.

B, C Time-course changes in the composition of [18F]FE-TMP and its radiometabolites in brain (B) and plasma (C). Relative amounts of radiomaterials are indicated as % of total radioactivities at each time point. Data from 3 mice at each time point were plotted as mean ± SD.

D Identification of a major metabolite, M1, by normal (left) and reverse (right) phase radio-HPLC. Retention times in HPLC charts were compared between radiosynthesized [18F]fluoroacetate ([18F]FAcOH) and radioactive metabolites derived from [18F]FE-TMP in mouse brain and plasma at 15 min after i.v. administration of [18F]FE-TMP. Retention time of [18F]FAcOH was identical to that of M1, a major radiometabolite of [18F]FE-TMP in brain.

E Radioactivity derived from [18F]FE-TMP (red area) and its radiometabolites (green area) in control cortex was calculated by applying temporal changes in their relative abundance (B) to the time-radioactivity curve shown in Fig EV2E. Data from control mice (n = 6) were plotted as mean ± SEM.

Source data are available online for this figure.

© 2021 The Authors

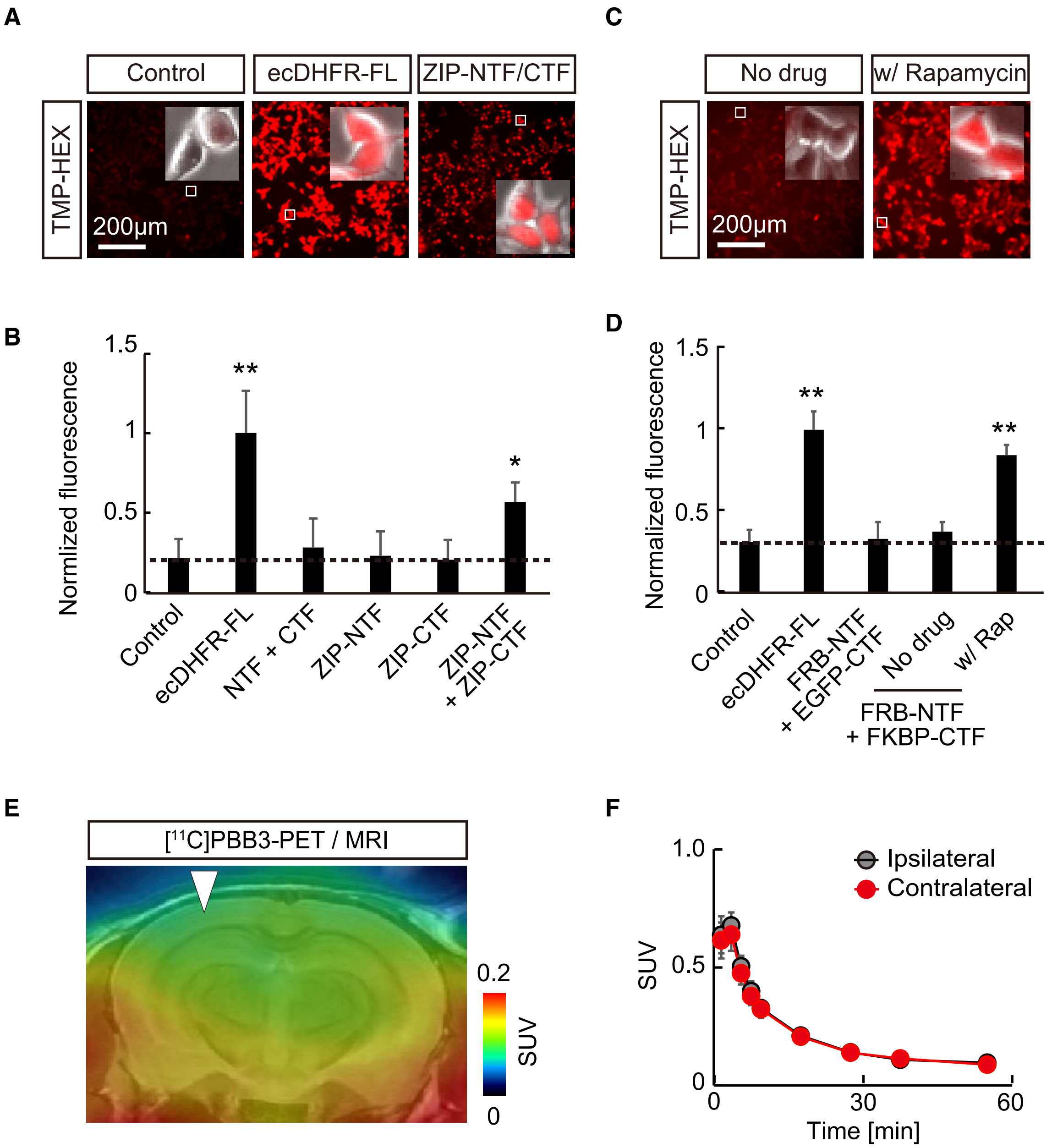

Figure EV5.

 © 2021 The Authors

Figure EV5. Design and characterization of protein fragment complementation assay using a split DHFR system.

A–D Cultured HEK293 cells expressing various constructs were incubated with 100 nM TMP-HEX and analyzed by fluorescent microscopy. TMP-HEX efficiently labeled cultured cells co-expressing NTF and CTF conjugated to a self-assembling leucine zipper (ZIP) motif, or a rapamycin-dependent heterodimerization motifs FKBP12-rapamycin binding domain (FRB) and FK506 binding protein (FKBP) in the rapamycin-dependent manner, indicating that the cDHFR-PCA system functions under these conditions as designed. (A) Representative images illustrate a selective retention of TMP-HEX in cells carrying full-length ecDHFR (as positive control) or a combination of ZIP-tagged ecDHFR-NTF and ecDHFR-CTF. Insets demonstrate high-magnification fluorescence images overlaid with phase-contrast pictures of individual cells. Note that ZIP-tagged ecDHFR-NTF and CTF preferentially localize in nucleus. (B) Normalized fluorescence intensities of TMP-HEX in cells transfected with indicated constructs. Mean value of full-length ecDHFR (ecDHFR-FL) was set as 1. Note that only ecDHFR-FL and the split protein that reassembles via ZIP interaction retain the marker above background levels. Data from six independent experiments are plotted as mean ± SD. $F(5, 28) = 21.12$; $^{*}P < 0.05$, $^{**}P < 0.01$ (one-way ANOVA followed by Dunnett post hoc test). (C) HEK293 cells expressing various constructs were incubated with 100 nM TMP-HEX with or without 500 nM rapamycin (LC Laboratories) and imaged by fluorescence microscopy. Representative images illustrate selective labeling of TMP-HEX in cells co-expressing ecDHFR-NTF and CTF tagged with FRB and FKBP in the presence of rapamycin. Insets demonstrate high-magnification fluorescence photomicrographs overlaid with phase-contrast images. (D) Rapamycin (Rap)-induced FRB-FKBP interaction was assessed as TMP-HEX labeling efficiency in cells expressing various combinations of ecDHFR-NTF and ecDHFR-CTF fragments. Fluorescence intensities were normalized by mean value of labeling of full-length ecDHFR (ecDHFR-FL) with TMP-HEX. Data from four independent experiments are presented as mean ± SD. $F(4, 15) = 55.08$; $^{**}P < 0.01$ (one-way ANOVA followed by Dunnett post hoc test).

E A representative coronal PET image captured with [^{11}C]PBB3, a potent PET tracer for aggregated tau fibrils, in mice expressing TRD-NTF and TRD-CTF by AAV injection into one side of somatosensory cortex. Averaged image of dynamic scan data at 0-60 min after i.v. injection of [^{11}C]PBB3 is shown. A template MRI image was overlaid for spatial alignment of the PET image. Arrowhead indicates injection site.

F Kinetics of [^{11}C]PBB3 in mouse brain during 60-min dynamic PET scan. VOIs were manually placed on ipsilateral and contralateral cortical areas for quantification. Data from four mice expressing TRD-NTF and TRD-CTF are plotted as mean ± SEM. Note there is no significant difference in radioactive signals of [^{11}C]PBB3 between ipsilateral and contralateral sides.

Source data are available online for this figure.
