# Supplementary material for: A genetically targeted reporter for PET imaging of deep neuronal circuits in mammalian brains
Source: EMBO J. 2021 Oct 12;40(22):e107757. doi: 10.15252/embj.2021107757 (PMC8591537; doi:10.15252/embj.2021107757)
Supplement: Supplementary file 9 [file EMBJ-40-e107757-s007.pdf]

THE EMBO JOURNAL

A genetically targeted reporter for PET imaging of deep neuronal circuits in mammalian brains

Masafumi Shimojo, Maiko Ono, Hiroyuki Takuwa, Koki Mimura, Yuji Nagai, Masayuki Fujinaga, Tatsuya Kikuchi, Maki Okada, Chie Seki, Masaki Tokunaga, Jun Maeda, Yuhei Takado, Manami Takahashi, Takeharu Minamihisamatsu, Ming-Rong Zhang, Yutaka Tomita, Norihiro Suzuki, Anton Maximov, Tetsuya Suhara, Takafumi Minamimoto, Naruhiko Sahara, and Makoto Higuchi
DOI: 10.15252/embj.2021107757

Corresponding author(s): Makoto Higuchi (higuchi.makoto@qst.go.jp) , Masafumi Shimojo (shimojo.masafumi@qst.go.jp)

Review Timeline:

Submission Date:	17th Jan 21
Editorial Decision:	29th Mar 21
Revision Received:	12th Jun 21
Editorial Decision:	6th Aug 21
Revision Received:	24th Aug 21
Accepted:	31st Aug 21

Editor: Karin Dumstrei

Transaction Report:

(Note: With the exception of the correction of typographical or spelling errors that could be a source of ambiguity, letters and reports are not edited. Depending on transfer agreements, referee reports obtained elsewhere may or may not be included in this compilation. Referee reports are anonymous unless the Referee chooses to sign their reports.)

1st Editorial Decision 29th Mar 2021

Dear Makoto,

Thank you for submitting your point-by-point response and for the follow up discussion about what can be done to address the raised concerns.

I appreciate your response and would like to invite you to submit a revised manuscript. Let me know if we need to discuss anything further

When preparing your letter of response to the referees' comments, please bear in mind that this will form part of the Review Process File, and will therefore be available online to the community. For more details on our Transparent Editorial Process, please visit our website:
https://www.embopress.org/page/journal/14602075/authorguide#transparentprocess

with best wishes

Karin

Karin Dumstrei, PhD
Senior Editor
The EMBO Journal

Instructions for preparing your revised manuscript:

Please make sure you upload a letter of response to the referees' comments together with the revised manuscript.

Please also check that the title and abstract of the manuscript are brief, yet explicit, even to non-specialists.

When assembling figures, please refer to our figure preparation guideline in order to ensure proper formatting and readability in print as well as on screen:
https://bit.ly/EMBOPressFigurePreparationGuideline

IMPORTANT: When you send the revision we will require
- a point-by-point response to the referees' comments, with a detailed description of the changes made (as a word file).
- a word file of the manuscript text.
- individual production quality figure files (one file per figure)
- a complete author checklist, which you can download from our author guidelines (https://www.embopress.org/page/journal/14602075/authorguide).
- Expanded View files (replacing Supplementary Information)
Please see out instructions to authors
https://www.embopress.org/page/journal/14602075/authorguide#expandedview

Please remember: Digital image enhancement is acceptable practice, as long as it accurately represents the original data and conforms to community standards. If a figure has been subjected to significant electronic manipulation, this must be noted in the figure legend or in the 'Materials and Methods' section. The editors reserve the right to request original versions of figures and the original images that were used to assemble the figure.

Further information is available in our Guide For Authors:
https://www.embopress.org/page/journal/14602075/authorguide

The revision must be submitted online within 90 days; please click on the link below to submit the revision online before 27th Jun 2021.

https://emboj.msubmit.net/cgi-bin/main.plex

Referee #1:

The manuscript by Shimojo et al describes a PET-fluorescent bimodal reporter system that leverages the use of ecDHFR variants and TMP ligands to monitor neuronal ensemble activities, protein turnover and complex formation in mice and to trace anatomical circuits in a marmoset. This work is impressive, creative and novel and the applications illustrate the versatility of this reporter system. However, the manuscript currently has several weaknesses, mainly in how the findings are reported and I believe that addressing these would profoundly improve this work.

Title: Consider changing "deep neuronal network" due to recent use of this term in machine learning/AI fields.
Figure 1 panel C: according to the legend this panel only shows average values and lacks STDEV or SEM values. The graph should be modified to show the error.
Figure 1 panel A-C: One key advantage of using in vivo 2-photon imaging is that one can track the same cells longitudinally, in response to differing treatments. Can the authors specify whether they were able to track the same cells during these experiments or whether they assessed different cells for each condition? If not, why did the authors choose to not assess TMP-HEX accumulation, washout, and blocking with TMP in the same mice/cells? Specifically, do the authors have data from the same cells from mice across all conditions, showing that they can track the kinetics of TMP-HEX and then once TMP-HEX washes out, re-inject TMP-HEX along with a saturating TMP dose to show the blockade in those same cells. The washout experiment is key because having knowledge of the full kinetics of TMP-HEX in necessary if it is to be used as an optical reporter system.
Figure 1 panel E: The hypothalamus seems to show a faint TMP signal here. This is also seen in Fig S2D and in Figure 2D (a bit more ventral here). What do the authors think this signal is? Do the authors think this signal is meaningful?
Figure 1 panels F, G: Can the authors pls specify the time points used to calculate the SUV data?
Figure S2: The mice were injected with the AAV in the forebrain but radiolabeled 18F-TMP signal is observed and quantified in the paraventricular region. In panel D signal is also seen in the brainstem and in another region (more ventral part of the brain, close to midbrain). Can the authors clarify why they think these other regions are showing up and whether they injected the AAV in the same region as where the SUV values are being calculated?
Figure 2 panel E: Why are the authors choosing to normalize over brainstem here and not over the

contralateral region as in Figure 1? Why is the no drug condition showing an SUVR over 1? Is there 18F-TMP enrichment in the ipsilateral hippocampus that was injected with AAV? The PET image in Fig 2D does not show that in the absence of CNO. Finally, how exactly did the authors measure postmortem fluorescent expression of ecDHFR and what value is being represented on the x-axis?
In Figure 2, why did the authors have to use two very different doses of CNO (0.3 and 10 mg/kg)?
In Figures 2 and S3, why did the authors not assess Fos or other IEG induction in the mouse?
Figure 3: In panel B, why is putamen noted with ecDHFR in parentheses and cortex is not? Also why is the hippocampus SUV not shown here, if the hippocampus is used to show lack of ecDHFR in panel C. Similarly, why is cerebellum ecDHFR-EGFP expression not shown in panel C? Whichever region is used a reference should be consistent for both measures. Also do the authors know why the cortex show relatively higher accumulation that putamen? Both regions received AAV but the SUVs (cortex and putamen) are not consistent with the representative image.
Figure 4: In panel D, there is a loss of 18F-MNI signal in the ipsilateral hemisphere in the no drug condition. In general, this hemisphere has lower accumulation of radioligand compared to the contralateral hemisphere. Is this image representative of the entire group of mice in this condition? If so, do the authors know what causes it?
Figure 4: In panel I, the images represent radioligand accumulation between 60-90 min but in panel J, the SUVR is calculated using accumulation data from 15-60 min. Why?
Do the authors know where ecDHFR-EGFP localizes at the subcellular level?
Did the authors experiment with any kinetic models to see if they could improve the TMP PET signal/images?
Page 2 , Line 7: consider changing "expressions" to "expression".
Page 3, line 5: consider changing "fluorescence" to "fluorescence-based"
Page 3, line 8: same as above
Page 7 line 17: should be "of a deep neural circuit"
Page 8, line 8: consider changing to " increased by a factor of 4"
Page 12 line 8: consider changing to " it validates a new strategy"
Page 12 line 10: conider changing "network" to "networks"

Referee #2:

This paper describes the evaluation and application of a PET tracer targeting ecDHFR in the rodent and primate brain. Until today, a suitable PET tracer monitoring gene expression in the brain is highly desirable. This paper describes [18F]FE-TMP to image ecDHFR expression in the mammalian brain. Although the paper and topic is interesting, the PET tracer suffers from major limitations:

1. The affinity of a suitable CNS PET tracer should be in the single digit nanomolar range (depending on the Bmax, which was not determined). The Kd is 29.1 nM and thus the affinity too low for a suitable PET tracer (Figure 1D).
2. Low BBB penetration and slow clearance from the brain (SUV should be >1.5): Peak SUV in tissue with no binding sites is 0.2 in mice (Figure 1E, Figure S2D) and 0.5 in primates (Figure 3B), which shows that the brain uptake is too low. The authors should check if the radioligand is a substrate of

efflux transporters by Pgp inhibition with elacridar.
3. Clearance half-time of a suitable CNS tracer should be less than 30 minutes: here the kinetics of the PET tracer are much too slow (Figure 1F, Figure S2E, Figure 3B).
4. Tracer should reach equilibrium conditions during the time span of a PET experiment for a reliable quantification and comparison of binding parameters between subjects: [18F]FE-TMP does not reach equilibrium conditions until 180 min after injection (Figure S2F)and can thus not be used for quantification.
5. A CNS PET tracer should have minimal evidence of radiometabolites in brain (via BBB passage or production in brain) for a reliable quantification: At 60 min, 50% of the [18F]FE-TMP signal is from metabolites and PET scans were performed until 180 min (Figure S10, Figure S2D)

Due to these reasons, I do not see a wide application of the tracer in preclinical or clinical studies.

Referee #3:

Shimojo and colleagues present data on the design and validation of ecDHFR-based reporters and their ligands for bimodal fluorescence and PET imaging of the spatial distribution, stability, and aggregation of genetically-targeted proteins in vivo in the brains of animals. Specifically, they labelled ecDHFR antagonist TMP-analogues and demonstrated potential to visualise these in vivo in mouse using fluorescence and PET imaging. They went on to explore whether TMP-PET can visualise neuronal networks in non-human primate and whether the concept can be translated to tracking and quantifying protein turnover (phosphodiesterase 10A) and complex formation (oligomerisation of tau protein). The concept of bimodal ecDHFR/TMP PET and fluorescence imaging is not new (see e.g. work by Selmyer et al.), however, application to image neuronal networks and protein dynamics seems novel and a specialty of this research group. The results of robust TMP-PET signal (using a novel, fluorinated TMP tracer) in neuronally interconnected (deep) brain regions is intriguing as are findings of tracer uptake that can be associated with protein interactions. This paper describes a complex study, whose results seem promising and of general interest to a variety of neuroscience fields.

I have a few rather conceptual questions and comments.

Major comments:
1. The authors are encouraged to explain the specific benefits of bimodal fluorescence and PET imaging within the discussed areas of application with regards to the joint and individual pros and cons of each modality. Specifically, discuss differences in field of view (superficial (how superficial, implications for application) vs whole-brain, concrete resolution, partial volume effects, and aspects of potential quantification of image/uptake signal. Would it be possible to quantify and compare information provided by each modality in regions where both can be employed (as in first part of experiments)?
2. Given that the authors propose parts of their methodology to be used for imaging resting and activated brain networks, how does their method compare to functional MRI given the availability of high-field small animal MRI? This would have been an informative addition to strengthen the network aspect of their work and to emphasize the unique contribution of their methodology to this aspect.
3. Could the proposed approach be modified as to image cell-specific contributions to brain networks and their activation (i.e. neuronal sub-types, neurons vs glia)?
4. What was the rationale behind assessing PDE10A and tau? Was it their role in neurodegenerative diseases? While that is mentioned for tau, nothing is mentioned about PDE10A

and its role in e.g. Huntington's disease. Regarding imaging soluble forms of self-aggregating proteins, another obvious candidate (also imageable with PET) would have been amyloid-beta.
5. The tau oligomerisation aspect of the paper is highly intriguing but quite speculative. I could not find any information on when exactly the PBB3 PET scans were performed, i.e. could fibrillisation of tau be expected at that timepoint?
6. The authors are encouraged to briefly discuss potential ways and challenges when translating their findings into application in humans.

Minor comments:
7. Please provide more information (kinetics, dynamics) on the new ligand [18F]FE-TMP in comparison to the established 11C-version.
8. Could marmoset not have been employed as model for the tau part of the study? Previous work has detailed tau pathology in marmoset (PMID: 31171723).

Point-by-point response to the referees' comments:

Please note that our replies to the reviewers' concerns are highlighted *in blue*.

Referee #1

1. "Title: Consider changing "deep neuronal network" due to recent use of this term in machine learning/AI fields."

We have accordingly changed this terminology to "deep neuronal circuit" in the title of the revised manuscript.

2. "Figure 1 panel C: according to the legend this panel only shows average values and lacks STDEV or SEM values. The graph should be modified to show the error."

According to this referee's suggestion, we have plotted the graph as Mean ± SD in this panel. Since the original ROI assay of initial experimental data from an animal was underpowered, we have included additional data which minimally changed the graph. We apologize for this confusion.

3. "Figure 1 panel A-C: One key advantage of using in vivo 2-photon imaging is that one can track the same cells longitudinally, in response to differing treatments. Can the authors specify whether they were able to track the same cells during these experiments or whether they assessed different cells for each condition? If not, why did the authors choose to not assess TMP-HEX accumulation, washout, and blocking with TMP in the same mice/cells? Specifically, do the authors have data from the same cells from mice across all conditions, showing that they can track the kinetics of TMP-HEX and then once TMP-HEX washes out, re-inject TMP-HEX along with a saturating TMP dose to show the blockade in those same cells. The washout experiment is key because having knowledge of the full kinetics of TMP-HEX in necessary if it is to be used as an optical reporter system."

Although we originally performed a time-course analysis of fluorescence on 10

cells per mouse in these experiments, the labeling kinetics of TMP-HEX with or without saturating TMP dose were assessed independently, with target cells differing among experiments. Following this referee's suggestion, we assessed the additional labeling kinetics of TMP-HEX in the same mouse/cells, followed by a re-assessment of these cells in the blocking condition with pre-administration of saturating dose of TMP the next day. As shown in Appendix Fig S1, fluorescence signals of TMP-HEX in neurons peaked at around 60 min post-injection and then gradually washed out, becoming close to a background level at 3 hours. This binding of TMP-HEX to ecDHFR in the same cells was also blocked by i.p. administration of a saturating dose of unlabeled TMP before the imaging session, supporting our original conclusion. Since TMP-HEX reagent is currently not available from Active Motif Inc., we could only coordinate this pilot analysis in a single mouse using our limited stock. This additional information has also been provided in the Results section of the revised manuscript (Page 4, line 28).

4. "Figure 1 panel E: The hypothalamus seems to show a faint TMP signal here. This is also seen in Fig S2D and in Figure 2D (a bit more ventral here). What do the authors think this signal is? Do the authors think this signal is meaningful?"

Since we frequently obtained similar findings even in the control mouse brain, which does not express ecDHFR reporters, we believe that this faint signal is attributable to a non-specific radioligand accumulation in the hypothalamus or radioactivity spillover from adjacent extracranial space. This point has been stated in the revised legend for Figure 1 (Page 35, line 25).

5. "Figure 1 panels F, G: Can the authors pls specify the time points used to calculate the SUV data?"

In these panels, we plotted the data at 1.5, 3.5, 7, 11, 15, 19, 27.5, 37.5, 47.5, 57.5, 67.5, 77.5, 87.5 min. This information has been added to the revised legend for Figure 1 (Page 36, line 1 and Page 36, line 5).

6. “Figure S2: The mice were injected with the AAV in the forebrain but radiolabeled 18F-TMP signal is observed and quantified in the paraventricular region. In panel D signal is also seen in the brainstem and in another region (more ventral part of the brain, close to midbrain). Can the authors clarify why they think these other regions are showing up and whether they injected the AAV in the same region as where the SUV values are being calculated?”

As stated in the Materials and Methods section, our procedure of AAV injections into a single side of the lateral cerebral ventricle in neonatal pups constantly produces an enriched distribution of reporter genes in regions adjacent to ventricles, including the retrosplenial cortex and hippocampus. This transduction technique also induces broad expression of reporters into neurons throughout the brain by the spreading of AAVs via the ventricular CSF. Moreover, as shown in Fig. EV1B, we also observed enriched expression of the reporter in several ventral parts of the brain. These uniform and enriched expression patterns in this condition reasonably match the distribution of the radioactive signal in PET, supporting the specificity and sensitivity of our reporter imaging technique with [^{18}F]FE-TMP. We have appended this information to the Results (Page 5, line 25) and Material and Methods (Page 16, line 3) sections in the revised manuscript.

7. “Figure 2 panel E: Why are the authors choosing to normalize over brainstem here and not over the contralateral region as in Figure 1? Why is the no drug condition showing an SUVR over 1? Is there 18F-TMP enrichment in the ipsilateral hippocampus that was injected with AAV? The PET image in Fig 2D does not show that in the absence of CNO. Finally, how exactly did the authors measure postmortem fluorescent expression of ecDHFR and what value is being represented on the x-axis?”

When we transduced the expression of ecDHFR-d2Venus reporters on the unilateral hippocampus by AAV, we observed a faint accumulation of radioactive signals on the non-injected contralateral side, which could reflect [^{18}F]FE-TMP binding to the reporters localized at the terminals of the commissural axonal projection originating from the injection site. For this reason, we chose the brainstem but not the contralateral hippocampus as a reference for SUVR

estimations, in light of the fact that the brainstem exhibited stably low background signals. In addition, we noted that there was a low-level but PET-detectable basal expression of the ecDHFR-d2Venus reporter in the ipsilateral hippocampus before CNO-mediated hM3Dq activation. This may be due to the reporter expression induced by physiological activation of hippocampal neurons related to animal behaviors. We are currently attempting to improve the performance of the RAM-mediated expression system by genetic engineering for the suppression of the basal gene expression, but such works will be beyond the scope of the current research. Finally, we apologize for the oversight in the x-axis of Fig. 2E. In these experiments, we sacrificed mice for a histochemical analysis at about 2 weeks after the PET scan with the activation of hM3Dq by CNO administration. To determine the expression level of the reporter, postmortem brain slice images were captured by a confocal microscope, as shown in Appendix Fig. S3. ROIs were then manually placed on the hippocampal region, and average fluorescence intensities of ecDHFR-d2Venus were measured. The background value of the non-infected hippocampal region was set as 1. This technical information has also been incorporated in the legend for Fig. 2 (Page36, line 28) in the revised manuscript.

8. “In Figure 2, why did the authors have to use two very different doses of CNO (0.3 and 10 mg/kg)?”

We initially characterized the activity-dependent time-course changes of ecDHFR-d2Venus expression by two-photon microscopic imaging as shown in Panels B and C of Fig. 2. In this experiment, it was required to activate the neocortical neurons by stimulating hM3Dq with 10mg/kg of CNO for constant inductions of the reporter gene expression. In contrast, we noted that the activation of hM3Dq in the hippocampal neurons following 10mg/kg of CNO administration frequently caused epileptic seizures and immediate death of the mice. This observation well matches the findings in the previous study describing the CNO dose-dependent seizure phenotype of hM3Dq transgenic mice (Alexander GM, et al. (2009) doi: 10.1016/j.neuron.2009.06.014), leading us to minimize the dose of CNO to 0.3mg/kg for the experiments shown in Panels D and E. We presume that this is probably due to the difference in the susceptibility to epileptic hyperexcitabilities between neocortical and

hippocampal neurons. We added this explanation in the legend for Fig. 2E (Page 36, line 26).

9. “In Figures 2 and S3, why did the authors not assess Fos or other IEG induction in the mouse?”

As stated above, the fluorescence signal from overexpressed ecDHFR-d2Venus could still be detectable in the injected site of the postmortem brain collected at about 2 weeks after the activation PET session, while the endogenous IEG induction quickly returned to basal level and did not provide precise spatiotemporal information of neuronal activity in this condition.

10. “Figure 3: In panel B, why is putamen noted with ecDHFR in parentheses and cortex is not? Also why is the hippocampus SUV not shown here, if the hippocampus is used to show lack of ecDHFR in panel C. Similarly, why is cerebellum ecDHFR-EGFP expression not shown in panel C? Whichever region is used a reference should be consistent for both measures. Also do the authors know why the cortex show relatively higher accumulation that putamen? Both regions received AAV but the SUVs (cortex and putamen) are not consistent with the representative image.”

According to this referee’s suggestion, we have added hippocampal SUVs to the graph shown in Panel B of Fig. 3 as reference data. In relation to this amendment, the legend for this figure has also been updated (Page 37, line 24). In this experimental condition, the regional expression level of the ecDHFR-EGFP reporter in the cortex was higher than that of the putamen, which matched the data obtained from postmortem brain slices as shown in Panel C. We manually placed VOIs for quantification of averaged %SUV in each region. Since the regional volume expressing the reporter protein at the injection site is relatively small in the putamen, the radioactive signal can be underestimated because of the partial volume effect. In contrast, the reporter protein can distribute broad volume in the cortex, and therefore, we speculate that the radioactive signal was less sensitive to the partial volume effect.

11. “Figure 4: In panel D, there is a loss of 18F-MNI signal in the ipsilateral hemisphere in the no drug condition. In general, this hemisphere has lower accumulation of radioligand compared to the contralateral hemisphere. Is this image representative of the entire group of mice in this condition? If so, do the authors know what causes it?”

To circumvent any misinterpretations of the PET findings, we have replaced the two images in Panel D of Fig. 4 with those representing the observations more adequately. In addition, it was described in the original legend for Fig. 4D that the images were generated by averaging dynamic data at 0 - 30 min, but the data were actually averaged at 30 – 60 min. This error has been amended in the revised manuscript (Page 38, line 23).

12. “Figure 4: In panel I, the images represent radioligand accumulation between 60-90 min but in panel J, the SUVR is calculated using accumulation data from 15-60 min. Why?
Do the authors know where ecDHFR-EGFP localizes at the subcellular level?
Did the authors experiment with any kinetic models to see if they could improve the TMP PET signal/images?

We apologize for causing this confusion. We have presented PET images generated by averaging dynamic scan data at 30 - 90 min in new Fig. 5D, and the SUVR graph has been updated with data from 30 - 90 min in new Fig. 5E (Page 40, line 15). Both changes minimally affected the primary findings and their indications. When we unilaterally transduce the overexpression of ecDHFR-EGFP in neurons of somatosensory cortex by AAV injection, we usually observe that the fluorescence signals derived from ecDHFR-EGFP are relatively enriched in somatodendritic compartments of these neurons but that they are also distributed to axonal fibers derived from these neurons, implicating relatively uniform subcellular distribution of ecDHFR-EGFP. This information was included in new Fig. EV1D and related legend in the revised manuscript (Page 41, line 12). Moreover, to address this reviewer’s concern, the %SUV data

shown in Fig. 1F were re-analyzed with the reference tissue Logan models and displayed as a scatterplot (Appendix Fig. S2). The linearity of the plot indicates the reversible radioligand binding to the target, and SUVR was closely correlated with the non-displaceable binding potential estimated by Logan's plot, supporting the validity of the SUVR measurement as a simplified quantitative method. This information has been included in the Results section of the revised manuscript (Page 6, line 10)

Page 2, Line 7: consider changing "expressions" to "expression".
Page 3, line 5: consider changing "fluorescence" to "fluorescence-based"
Page 3, line 8: same as above
Page 7 line 17: should be "of a deep neural circuit"
Page 8, line 8: consider changing to " increased by a factor of 4"
Page 12 line 8: consider changing to " it validates a new strategy"
Page 12 line 10: conider changing "network" to "networks"

We corrected the text according to this referee's suggestions on (Page 2, line 7; Page 3, line 4 and 8; Page 8, line 7; Page 8, line 20; Page 13, line 16 and 18)

Referee #2

1. "The affinity of a suitable CNS PET tracer should be in the single digit nanomolar range (depending on the Bmax, which was not determined). The Kd is 29.1 nM and thus the affinity too low for a suitable PET tracer (Figure 1D).

We agree that a large subset of PET radiotracers reacts with their target molecules with a Kd value below 10 nM. Our prototypical radioligand, [^{11}C]TMP, almost fulfilled this criterion (Kd ~ 10 nM), and the affinity of [^{18}F]FE-TMP for ecDHFR (~30 nM) was considerably lower than that of [^{11}C]TMP (Fig. 1D). Meanwhile, [^{18}F]FE-TMP yielded a much larger in vivo contrast for ecDHFR expressed by intraventricular injection of AAV vector than [^{11}C]TMP (new Fig. EV2), primarily owing to fast washout of the tracer in a non-displaceable

compartment. Indeed, the ratios of radioactivity between ecDHFR-expression and control brains in a steady-state were ~2.5 and ~6.0 in PET imaging with [^{11}C]TMP and [^{18}F]FE-TMP, respectively (Panels C and F in Fig. EV2). The in vivo contrast for the site-directed radiotracer binding could depend on the Bmax/Kd value and levels of background (non-displaceable) tracer retentions. According to previous reviews (Patel & Gibson, Nucl Med Biol 2008, doi 10.1016/j.nucmedbio.2008.10.002), Bmax/Kd ≥ 10 is considered preferable for in vivo imaging, whereas several radioligands as exemplified by [^{11}C]cocaine and [^{11}C]raclopride with in vitro and in vivo Bmax/Kd values below 10 have been utilized for PET investigations. The non-displaceable binding potential of [^{18}F]FE-TMP, which could be equivalent to Bmax/Kd and could be estimated as [(target-to-reference ratio of radiotracer retention) – 1.0], was ~5.0 in the mouse brain following intraventricular AAV injection (Fig. EV2F) and may vary as a function of ecDHFR expression levels. In addition, we are currently performing structural modifications of FE-TMP to obtain compounds with a higher affinity for ecDHFR, which will be documented in a separate report. These technical considerations have been included in the Results (Page 5, line 8) and Discussion (Page 11, line 27) sections of the revised manuscript.

2. “Low BBB penetration and slow clearance from the brain (SUV should be >1.5): Peak SUV in tissue with no binding sites is 0.2 in mice (Figure 1E, Figure S2D) and 0.5 in primates (Figure 3B), which shows that the brain uptake is too low. The authors should check if the radioligand is a substrate of efflux transporters by Pgp inhibition with elacridar.”

In accordance with the referee’s suggestion, we conducted PET imaging with [^{18}F]FE-TMP following pre-treatment of animals with 3 mg/kg of elacridar, a potent inhibitor of Pgp. As shown in Appendix Fig S6, the elacridar treatment resulted in elevated radiotracer uptake into the brain, indicating the possibility that [^{18}F]FE-TMP is a moderate substrate for Pgp. This information was included in the discussion (Page 12, line 13). Although several PET tracers, including [^{11}C]WAY-100635, [^{11}C](R)-RWAY, and [^{18}F]MPPF, are known to be substrates for Pgp (Pike, Trends Pharmacol Sci 2009, doi: 10.1016/j.tips.2009.05.005), a preferable imaging agent would be designed not to react with this and other efflux transporters. As mentioned above, we are currently working on further

chemical engineering of FE-TMP derivatives to enhance the BBB permeability, and therefore identification of compounds with resistance to Pgp transporter would improve the dynamic range of reporter PET imaging.

3. “Clearance half-time of a suitable CNS tracer should be less than 30 minutes: here the kinetics of the PET tracer are much too slow (Figure 1F, Figure S2E, Figure 3B).”

As the referee commented, faster clearance of the radioligand from the brain would be more suitable for high-contrast imaging and robust pharmacokinetic assessments. In mice, about half of [^{18}F]FE-TMP radiosignals were cleared from the brain in approximately 30 min after their peak uptake (Fig. EV2E), which may be suitable for imaging of the target with reasonably high contrasts. In a marmoset, the radiotracer uptake was higher, but its clearance was slower than in mice, raising the possibility that the marmoset Pgp reacts with [18F]FE-TMP more weakly than does the murine Pgp. Although the washout of [^{18}F]FE-TMP from the marmoset brain is not rapid, the relatively long radioactivity half-life of ^{18}F (~110 min) allowed the pursuit of the kinetics of specifically bound versus non-displaceable tracers over a long period of up to 180 min. We have added more discussion about the capacity of [^{18}F]FE-TMP in the revised manuscript. (Page 11, line 23 and Page 12, line 13)

4. “Tracer should reach equilibrium conditions during the time span of a PET experiment for a reliable quantification and comparison of binding parameters between subjects: [18F]FE-TMP does not reach equilibrium conditions until 180 min after injection (Figure S2F)and can thus not be used for quantification.”

As stated above, we have provided more detailed discussion on the kinetics of [^{18}F]FE-TMP in the revised manuscript. In most of the PET experiments, radioligand binding reached a pseudo-equilibrium state within 90 min after the intravenous injection. In the plots shown in Fig EV2, SUV ratios fluctuated at late time points due to very low radioactivity in the control animal but plateaued at ~150 min (Page 11, line 26).

5. “A CNS PET tracer should have minimal evidence of radiometabolites in brain (via BBB passage or production in brain) for a reliable quantification: At 60 min, 50% of the [18F]FE-TMP signal is from metabolites and PET scans were performed until 180 min (Figure S10, Figure S2D).”

As described in the Materials and Methods section of the original manuscript, we conducted a detailed analysis of radiometabolites derived from [^{18}F]FE-TMP. There are indeed two major radiometabolites of [^{18}F]FE-TMP, termed M1 and M2, as shown in new Fig EV4. M1 is a major radiometabolite in the brain, and the fraction of this metabolite gradually increased over 90 min. In contrast, M2 is a major radioactive metabolite in plasma, and its fraction reached a plateau at 15 min. Since the amount of M2 detected in the brain is very low, it is unlikely that this radiometabolite undergoes efficient transfer from plasma to the brain. Besides, M1 is almost undetectable in plasma and is accordingly presumed to be converted from [^{18F}]FE-TMP in the brain. We have also identified M1 as [^{18}F]fluoroacetate ([^{18}F]FAcOH), and this radiochemical should not react with ecDHFR in consideration of its chemical structure. The calculated contributions of [^{18}F]FE-TMP and its metabolites to total radioactivity in the brain were appended to the control time-radioactivity curve in Fig EV4E. Moreover, we found no marked increase of radiosignals in PET scans of mice receiving control AAV vector encoding mCherry only (Fig 1E), supporting the notion that the generation of [^{18}F]FAcOH in the brain does not non-specifically accumulate in the lesioned site. We now moved this information into the Results section (Page 6, line 28) and added more discussion (Page 12, line 26) in the revised manuscript.

6. “Due to these reasons, I do not see a wide application of the tracer in preclinical or clinical studies.”

As stated in the original manuscript, this is the first generation of a reporter system, and the pharmacokinetics and pharmacodynamics of the tracer along with the reactivity of ecDHFR can be largely improved by chemical and protein engineering. Regarding this point, we are currently working on the combinatorial screening of ecDHFR mutants and FE-TMP derivatives, and preliminary data

have strongly encouraged us to construct an advanced reporter system with much better performance. Although those findings are still beyond the scope of the present manuscript, future continuous efforts may allow us to coordinate more advanced utilities of brain reporter imaging in preclinical and clinical studies. In addition, we believe that the present work has already demonstrated diverse applications of the FE-TMP-ecDHFR system, including gene expression reporting, pharmacological switching of PDE10A, assessment of tau oligomerization, and visualization of neuronal activations.

Referee #3

Major comments:

1. “The authors are encouraged to explain the specific benefits of bimodal fluorescence and PET imaging within the discussed areas of application with regards to the joint and individual pros and cons of each modality. Specifically, discuss differences in field of view (superficial (how superficial, implications for application) vs whole-brain, concrete resolution, partial volume effects, and aspects of potential quantification of image/uptake signal. Would it be possible to quantify and compare information provided by each modality in regions where both can be employed (as in first part of experiments)?

We thank the referee for the insightful comments. We have added more information and discussion on this topic in the revised manuscript (Page 10, line 26).

2. “Given that the authors propose parts of their methodology to be used for imaging resting and activated brain networks, how does their method compare to functional MRI given the availability of high-field small animal MRI? This would have been an informative addition to strengthen the network aspect of their work and to emphasize the unique contribution of their methodology to this aspect.”

We are preliminarily developing genetically engineered mouse models expressing ecDHFR reporter genes regulated by IEG promoters which will enable the non-invasive imaging of physiological and pathological (e.g. epileptic)

brain network activations. The advantage of this and related approaches is that we can potentially label a specific neuronal population, such as glutamatergic versus GABAergic systems, in response to various sensory stimuli or behavioral tasks, and the in vivo observation can also be validated by ex vivo assessments of the reporter expression that were not possible by conventional functional MRI. In addition, once functional connectivity between two brain regions is indicated by functional MRI, the actual fiber projection linking these areas, along with plasticity changes of such a network, could be clearly demonstrated by IEG reporter PET following injection of an AAV vector into the putative origin of the target neural pathway. We have added more information on this topic in the Discussion section of the revised manuscript (Page 14, line 18).

3. “Could the proposed approach be modified as to image cell-specific contributions to brain networks and their activation (i.e. neuronal sub-types, neurons vs glia)?”

We believe that cell-specific reporter imaging is performable. A transgenic animal expressing ecDHFR under the control of a cell-type-specific promoter would offer a more advanced physiological or pathophysiological analysis of a neuronal or glial subtype of interest across brain regions. For instance, the utility of the other type of IEG promoters, such as Arc and NPAS4, may enable analysis of activations of different neuronal subtypes. Alternatively, incorporation of a cell-type-specific promoter in conjunction with the Cre-loxP system may also allow us to selectively visualize activations of a neural network in a single neurotransmission system. We have included this information in the Discussion section of the revised manuscript (Page 14, line 11).

4. “What was the rationale behind assessing PDE10A and tau? Was it their role in neurodegenerative diseases? While that is mentioned for tau, nothing is mentioned about PDE10A and its role in e.g. Huntington's disease. Regarding imaging soluble forms of self-aggregating proteins, another obvious candidate (also imageable with PET) would have been amyloid-beta.”

We apologize for the somewhat misleading presentations of separate assays on PDE10A and tau. The experiments displayed in Fig 4A-4E and Fig 4F-4J were conducted based on totally independent rationales. When we submitted the original manuscript, we expected that both topics were related to protein engineering and should be included in the same figure. However, to avoid confusion, we have split these panels into two isolated figures in the revised manuscript. Although the current proof-of-concept study was focused on monitoring tau protein aggregation as a model case, this technology is potentially applicable to a PET assessment of amyloid-beta and alpha-synuclein fibrillogenesis in the brain of a living animal.

5. “The tau oligomerisation aspect of the paper is highly intriguing but quite speculative. I could not find any information on when exactly the PBB3 PET scans were performed, i.e. could fibrillisation of tau be expected at that timepoint?”

In this experiment, we co-injected AAVs encoding TRD-NTF and TRD-CTF in the somatosensory cortex of 2-month-old mice and performed a sequential PET scan with [^{11}C]PBB3 and [^{18}F]FE-TMP a month after the surgical procedure. As demonstrated in Fig EV5E, fibrillation of tau has not been detected by PET with [^{11}C]PBB3, and this observation was also confirmed by negative PBB3 fluorescence staining of brain slices derived from these mice (data not shown). We previously reported that the rTg4510 mouse model of tauopathy develops progressive tau depositions, which were successfully captured by histological analysis and PET scan with [^{11}C]PBB3 at around 4-5 months of age (Ishikawa A et al. J Alzheimer Dis 2018). Given the robust overexpression of disease-associated mutant tau from early postnatal periods in rTg4510 mice, we speculate that AAV-mediated tau expression may require a longer period to provoke [^{11}C]PBB3-PET-visible tau depositions. In this regard, future efforts would be necessary to determine the precise time-course change of tau pathologies from oligomerization to mature fibril formation in AAV-injected model animals. Moreover, biochemical assays for sensitive detection of tau oligomers in the excised brain tissue need to be established since only a small amount of TRD multimers was yielded from this model. We added this point in the Discussion section of the revised manuscript (Page 13, line 27).

6. “The authors are encouraged to briefly discuss potential ways and challenges when translating their findings into application in humans.”

We thank this referee for indicating this important perspective. We have added discussion on this point in the revised manuscript (Page 14, line 26).

Minor comments:

7. “Please provide more information (kinetics, dynamics) on the new ligand [18F]FE-TMP in comparison to the established 11C-version.

The brief information about the comparison between [^{11}C]TMP and [^{18}F]FE-TMP was described in the section of Materials and Methods in the original manuscript. According to the referee’s suggestion, we moved this paragraph with expanded information to the Results section of the revised manuscript (Page 5, line 8).

8. “Could marmoset not have been employed as model for the tau part of the study? Previous work has detailed tau pathology in marmoset (PMID: 31171723).”

We appreciate this suggestion by the referee. Sharma et al. investigated the expression and phosphorylation status of marmoset tau proteins. Their study showed that tau isoform expression in the marmoset was similar to that in the mouse, and tau phosphorylation in the adult marmoset was much less than that in the newborn marmoset. We expect that marmosets may not cause pathological tau accumulation during their lifespan. It would require a future investigation to introduce tau oligomerization in marmoset brains.

1st Revision - Editorial Decision 6th Aug 2021

Dear Dr. Higuchi,

Thank you for submitting your revised manuscript to The EMBO Journal. Your study has now been seen by referees #1 and 2. As you can see both referees find that the analysis has been strengthened. Referee #2 still has some outstanding issues. I believe that most of them should be straightforward to address. Please get back to me so that we can discuss the remaining concerns further.

When you submit the revised version will you also take care of the following points:

- The author Yuji Nagai needs to be entered in the online system.

- In the Data availability section just state This study contains no data deposited in external repositories.

- In the author contribution section please differentiation between Takafumi Minamimoto and Takeharu Minamihisamatsu & Norihiro Suzuki and Naruhiko Sahara

- In the appendix file there is a typo in the ToC 'legened'

- Figure callouts to Appendix figures S9&10 should be "Appendix Figure S9" etc.

- For Figure 1C as n=2 best to provide both data points and not include statistic

- For Figs 3C & EV5A/C, please mark the magnified area (shown in the inset) in the low magnification image.

- We include a synopsis of the paper (see http://emboj.embopress.org/). Please provide me with a general summary statement and 3-5 bullet points that capture the key findings of the paper.

- We also need a summary figure for the synopsis. The size should be 550 wide by [200-400] high (pixels). You can also use something from the figures if that is easier.

- Please upload source data as one file per figure

That should be all.

with best wishes

Karin

Karin Dumstrei, PhD
Senior Editor
The EMBO Journal

Guide For Authors: https://www.embopress.org/page/journal/14602075/authorguide

Revision to The EMBO Journal should be submitted online within 90 days, unless an extension has been requested and approved by the editor; please click on the link below to submit the revision online before 4th Nov 2021:

https://emboj.msubmit.net/cgi-bin/main.plex

Referee #1:

The authors have done a great job addressing my comments. Thank you

Referee #2:

The authors have thoroughly addressed all the concerns of the reviewers. I think the idea of the study is very good, however the tracer is not appropriate in his current form and does therefore not support the hypothesis. However it gives a good indication what can be done with a better PET reporter in place. My concerns are addressed below.

Major concerns:

Point by point response:
Appendix Figure 6: Elacidar experiment is missing in the revised version of the manuscript.
The authors indicate that equilibrium conditions (target versus ref region) were obtained for most PET experiments 90 min after i.v. injection. This is not shown in the manuscript and representative DVR-1 curves should be provided.

Figure 1E-G:
E: the sagittal MR image shown for the control is more in the middle of the brain. Please show a comparable slice for both groups. Please also show ipsi- and contralateral TACs and SUVRs of the control group for a better comparison. In addition, please add a representative fluorescence image indicating EGFP and mCherry expression in the figure to show that the transgene expression was the same in both groups. Why did the authors choose mCherry and not EGFP as control vector?

Figure 3:
What is the control-AAV?
DVR-1 values (target versus ref. region)-1 should be provided.

Figure 5E:
There seems to be one outlier in the TRD-NTF + TRD-CTF group with a much higher SUV than the remaining animals in the group.
1. Was this animal chosen in Figure 5E? If yes, I would not say it is a representative animal and should be exchanged.
2. What happens to the significance if this animal is removed from the statistical analysis? This should be stated in results and discussion.

M&M:
The authors state that the virus titer was 2x109-8x109 for the AAV experiments.

1. This is a rather low titer and will reveal a low transgene expression. Why did the authors use this low expression?
2. The authors should indicate the exact titer injected for ecDHFR and control groups in the respective experiments. A titer of 8x109 is 4-times higher than a titer of 2x109 and will thus result in a 4-times higher transgene expression. If the control animals have a 4-times lower transgene expression, it would not be a valid comparison.

Results:
The authors should provide IHC of ecDHFR expression not just IF to show the ecDHFR expression. Please show the viral vector constructs used for ecDHFR expression and controls (Promoter, enhancer elements etc.) in the figures.

Figure EV2/SUVR
The authors show the tracer uptake in the brain of ecDHFR and Tomato control mice and take the ratio of these values from two different animals.
1.) Why is the NSB in the Tomato-injected control rats different from ecDHFR rats?
2.) To calculate the specific binding as DVR-1, the reference region needs to be selected in the same animal, f.e. is the cerebellum often used as reference region. Also in this model, the cerebellum is not affected by the AAV expression. Please show the DVR-1 in Tomato-expressing rats versus ecDHFR-expressing rats.
3.) Please also indicate in the Figure EVF2 what the control exactly is, f.e. tdTomato (control) on the left side of the images.

In all the figures, the %SUV is shown, instead of SUV. Please change this in the whole manuscript, as SUV is the gold standard and makes it easier to compare the compound to other published PET tracers.

EVF5:
What was the rationale to measure Tau-PET? I think it is not surprising that the authors did not observe a signal with 11C-PBB3 PET as the tracer does not bind to dimers but fibrillary structures. Did the authors expect that the dimers form aggregates in vivo? The rationale of this experiment is not clear to me.

EVF3B:
Fluorescent images: The transgene expression of dTomato seems to be much weaker and more distributed over the brain compared to EGFP and at a different location (cortex and right hippocampus). Looks like an injection into the cerebral ventricle as shown in figure EV1B.
If this is the case, the dTomato group is not a proper control group as the protein expression should be the same. This emphasizes that the control should also not be used to calculate SUVRs as in EVF2. A better control would be animals injected with AAV-EGFP in the same brain area.

Point-by-point responses to the editor’s and referee’s comments:

Please note that our replies are highlighted *in blue*.

According to Editorial suggestion, we have changed following points in the revised manuscript.

- The author Yuji Nagai needs to be entered in the online system.

We will add his name to the online system.

- In the Data availability section just state This study contains no data deposited in external repositories.

We have corrected this statement in the revised version of the manuscript.

- In the author contribution section please differentiation between Takafumi Minamimoto and Takeharu Minamihisamatsu & Norihiro Suzuki and Naruhiko Sahara

We have differentiated these authors as T. Minamimoto, T. Minamihisamatsu, N. Suzuki, and N. Sahara in the author contribution section of the revised manuscript.

- In the appendix file there is a typo in the ToC 'legened'

We have corrected them in the revised version of the manuscript.

- Figure callouts to Appendix figures S9&10 should be "Appendix Figure S9" etc.

Please provide us with more detailed information on how we should amend the callouts, such as “Appendix Fig 1” in the current manuscript.

- For Figure 1C as n=2 best to provide both data points and not include statistic

We have accordingly corrected this in Fig1C and related legend and source data.

- For Figs 3C & EV5A/C, please mark the magnified area (shown in the inset) in the low magnification image.

We have accordingly marked the magnified area with a white square in these figures. Since it was difficult to re-identify the same cells shown in the inset of the left panel in the original Fig EV5C, we have set a new area and have updated the inset image in the revision of this figure.

- We include a synopsis of the paper (see http://emboj.embopress.org/). Please provide me with a general summary statement and 3-5 bullet points that capture the key findings of the paper.

This information is submitted as the synopsis file separately.

- We also need a summary figure for the synopsis. The size should be 550 wide by [200-400] high (pixels). You can also use something from the figures if that is easier.

A summary figure is also included in the synopsis document.

- Please upload source data as one file per figure

The source data file per each figure is uploaded separately.

Referee #2:

The authors have thoroughly addressed all the concerns of the reviewers. I think the idea of the study is very good, however the tracer is not appropriate in his current form and does therefore not support the hypothesis. However it gives a good indication what can be done with a better PET reporter in place. My concerns are addressed below.

Major concerns:

Point by point response:
Appendix Figure 6: Elacidar experiment is missing in the revised version of the manuscript. The authors indicate that equilibrium conditions (target versus ref region) were obtained for most PET experiments 90 min after i.v. injection. This is not shown in the manuscript and representative DVR-1 curves should be provided.

We have confirmed that Appendix Fig 6, which contained the results of the elacidar experiment, existed in the previous revision of the manuscript (Page12, lines 9-24). Since the radioligand was administered to wild-type mice without ecDHFR expressions, there were no specific binding components for the ligand. Hence, this experiment was not aimed to examine the equilibrium of the ligand binding to ecDHFR.

Figure 1E-G:
E: the sagittal MR image shown for the control is more in the middle of the brain. Please show a comparable slice for both groups. Please also show ipsi- and contralateral TACs and SUVRs of the control group for a better comparison. In addition, please add a representative fluorescence image indicating EGFP and mCherry expression in the figure to show that the transgene expression was the same in both groups. Why did the authors choose mCherry and not EGFP as control vector?

According to this referee's suggestion, we have replaced the PET/MR image in the right panel of Fig 1E and have updated panels 1G, 1H, and 1I to include TACs and SUVRs of the mCherry group in the revised Manuscript (Page 6, line 6). We have also added representative fluorescence images of the postmortem brains that had received AAVs encoding ecDHFR-EGFP or mCherry to panel 1F.

We incorporated mCherry in the control vector since this enabled easy visual identification of the genotype based on the fluorescence color in the postmortem analysis, as shown in Fig 1F. Prior to this study, we had already tested PET imaging of control mice that received either PBS or AAVs encoding diverse fluorescence proteins, including EGFP, in the same experimental condition. We had found that none of these mice demonstrated any significant accumulation of radioactive signals in the injected site.

Figure 3:
What is the control-AAV? DVR-1 values (target versus ref. region)-1 should be provided.

In this experiment, control AAV encoding kappa-opioid receptor DREADD (KORD) with hSyn promoter was injected into the other side of the same animal brain. This information was included in the revised version of the manuscript (Page 17, line 7) and Appendix Table S1. Since the primary purpose of this experiment was to visualize the neural network connections containing the injection site but not to measure the radioligand binding in each region, we displayed SUV's in several brain area in the previous revision of the manuscript. However, according to this referee's suggestion, we have also demonstrated BP_{ND} *(DVR-1, Logan Reference) value in Appendix Fig S9 (Page 8, line 17) which further support the our findings and conclusion.*

Figure 5E:
There seems to be one outlier in the TRD-NTF + TRD-CTF group with a much higher SUV than the remaining animals in the group.
1. Was this animal chosen in Figure 5E? If yes, I would not say it is a representative animal and should be exchanged.
2. What happens to the significance if this animal is removed from the statistical analysis? This should be stated in results and discussion.

According to the referee's suggestion, we have replaced the image with a new one that may be more representative. The radioligand SUV in this experiment may exhibit substantial variability among individuals since only a subset of cells expressing TRD at very high levels could yield abundant dimers, resulting in a profound intensification of PET radiosignals. Hence, we believe that the individual showing the highest SUV is not an outlier but reflects particularly high concentrations of tau assemblies that may occur with a certain probability. This notion would be proven by examining the imaging-histology relationship, but

such additional assays would require substantive time. In addition, it is currently difficult to handle the efficiency of the AAV infection to each cell, and therefore the individual variability of the transgene expression could not be pronouncedly reduced.

M&M:
The authors state that the virus titer was 2x109-8x109 for the AAV experiments.
1. This is a rather low titer and will reveal a low transgene expression. Why did the authors use this low expression?
2. The authors should indicate the exact titer injected for ecDHFR and control groups in the respective experiments. A titer of 8x109 is 4- times higher than a titer of 2x109 and will thus result in a 4-times higher transgene expression. If the control animals have a 4-times lower transgene expression, it would not be a valid comparison.

In this study, we prepared homemade AAVs, and our purification protocol constantly produced the titer range shown in the Material and Methods section of the manuscript. We agree that our virus titer may be slightly lower than those of commercially available AAVs, which approximate 1-2 x10^{10}vg/μL. Nonetheless, we observed that our AAVs packaged with serotype DJ transduced high expression levels of transgene similar to commercial products with the same set of promoter and enhancers, and therefore, our AAV system may have advantages regarding the infection toxicity and transduction efficiency. According to this referee's suggestion, we have added a summary of viral vector constructs with information on the injected virus titer in Appendix Table S1.

Results:
The authors should provide IHC of ecDHFR expression not just IF to show the ecDHFR expression. Please show the viral vector constructs used for ecDHFR expression and controls (Promoter, enhancer elements etc.) in the figures.

Since we could not find any good commercially available antibody against the amino acid sequence of ecDHFR, it would be very difficult to coordinate this experiment promptly. Also, it is a straightforward strategy to utilize fluorescent protein fused to uncharacterized proteins or reporter gene products for investigation, and this technique is widely accepted and commonly utilized. We have provided information on the viral vector constructs in Appendix Table S1 as stated above.

Figure EV2/SUVR
The authors show the tracer uptake in the brain of ecDHFR and Tomato control mice and take the ratio of these values from two different animals.
1.) Why is the NSB in the Tomato-injected control rats different from ecDHFR rats?
2.) To calculate the specific binding as DVR-1, the reference region needs to be selected in the same animal, f.e. is the cerebellum often used as reference region. Also in this model, the cerebellum is not affected by the AAV expression. Please show the DVR-1 in Tomato- expressing rats versus ecDHFR-expressing rats.
3.) Please also indicate in the Figure EVF2 what the control exactly
is, f.e. tdTomato (control) on the left side of the images.

As we replied to the sixth comment by the first referee in the first revision, this neonatal injection technique constantly transduces enriched transgene expression in regions adjacent to the ventricles, including the retrosplenial cortex and hippocampus, in addition to lower but uniform expressions in widespread brain regions, including the cerebellum. Hence, increased radiosignals in extensive areas of the ecDHFR-expressing mouse brain are not attributable to non-specific radioligand retention but do stem from specific radioligand binding to the target molecules. This information was already described in previous publications (Chakrabarty et al, 2013; Kim et al, 2013), and our PET data also clearly demonstrate that the radioactive signals are also intensified in the cerebellum (please compare the top and bottom panels of Fig EV2D). Therefore, it would be virtually impossible to define a reference (control) region for the estimation of the radioligand binding as BP_{ND} or (DVR - 1) in this experimental model, giving a rationale for the calculation of SUVR between the ecDHFR-expressing and control mouse brains. According to this referee's suggestion, we have clearly indicated "tdTomato" as the control index in the revised Fig EV2.

In all the figures, the %SUV is shown, instead of SUV. Please change this in the whole manuscript, as SUV is the gold standard and makes it easier to compare the compound to other published PET tracers.

According to this referee's suggestion, we have indicated SUV instead of %SUV in all figures showing the radioactivity uptake. Related source data have also been updated.

EVF5:
What was the rationale to measure Tau-PET? I think it is not surprising that the authors did not observe a signal with 11C-PBB3 PET as the tracer does not bind to dimers but fibrillary structures. Did the authors expect that the dimers form aggregates in vivo? The rationale of this experiment is not clear to me.

Low-order tau aggregates have been recently hypothesized to be key components of the tau-triggered neurodegenerative pathway. Detection of tau dimers and oligomers would be accordingly beneficial for early diagnosis and efficacy assessments of candidate drugs counteracting the neurotoxic tau species, but in vivo monitoring of the formation of tau oligomers has not been achieved by standard tau-PET imaging technologies, which preferentially visualize densely packed high-order tau fibrils. We coordinated the current experiment to demonstrate the advantages of our ecDHFR-based PET reporter assay over existing tau PET assays for capturing initial tau assemblies putatively related to neuronal deteriorations. This methodology is also applicable to assess the tau dimerization in the presence or absence of endogenous or exogenous modifiers of the tau fibrillogenesis for elucidation of the etiological and therapeutic mechanisms.

EVF3B:
Fluorescent images: The transgene expression of dTomato seems to be much weaker and more distributed over the brain compared to EGFP and at a different location (cortex and right hippocampus). Looks like an injection into the cerebral ventricle as shown in figure EV1B. If this is the case, the dTomato group is not a proper control group as the protein expression should be the same. This emphasizes that the control should also not be used to calculate SUVRs as in EVF2. A better control would be animals injected with AAV-EGFP in the same brain area.

This experiment aims to make sure the specific binding ability of radioactive PET tracer onto the brain region expressing ecDHFR reporter transgene. For this reason, we selected brain slices with a clear enrichment of ecDHFR expression to facilitate our understanding and conclusion. As the quantum yield of EGFP and tdTomato may differ, transgene expressions in these slices could not be compared by quantifying fluorescence signals derived from the two fluorescence proteins. Even if we inject AAV-ecDHFR-EGFP and AAV-EGFP into the same

area of the two different mice, there is a possibility of considerable differences in the gene expression levels and/or fluorescence intensities between these individuals, primarily due to the variability of the infection efficiencies. In addition, the autoradiographic analysis implemented here is generally considered to be a sensitive method to detect radioligand binding to a very small area. Besides these facts, we could not find any rationale for the use of AAV-EGFP as the control instead of AAV encoding mCherry, tdTomato, and other proteins, none of which would bind to the PET radioligands.

2nd Revision - Editorial Decision 31st Aug 2021

Dear Makoto,

Thank you for submitting your revised manuscript. I have now had a chance to take a careful look at it and all looks good!

I am therefore very pleased to accept the manuscript for publication here.

With best wishes

Karin

Karin Dumstrei, PhD
Senior Editor
The EMBO Journal

Please note that it is EMBO Journal policy for the transcript of the editorial process (containing referee reports and your response letter) to be published as an online supplement to each paper. If you do NOT want this, you will need to inform the Editorial Office via email immediately. More information is available here:
https://www.embopress.org/page/journal/14602075/authorguide#transparentprocess

Your manuscript will be processed for publication in the journal by EMBO Press. Manuscripts in the PDF and electronic editions of The EMBO Journal will be copy edited, and you will be provided with page proofs prior to publication. Please note that supplementary information is not included in the proofs.

Please note that you will be contacted by Wiley Author Services to complete licensing and payment information. The 'Page Charges Authorization Form' is available here:
https://www.embopress.org/pb-assets/embo-site/tej_apc.pdf

Should you be planning a Press Release on your article, please get in contact with embojournal@wiley.com as early as possible, in order to coordinate publication and release dates.

If you have any questions, please do not hesitate to call or email the Editorial Office. Thank you for your contribution to The EMBO Journal.

** Click here to be directed to your login page: https://emboj.msubmit.net

EMBO PRESS

YOU MUST COMPLETE ALL CELLS WITH A PINK BACKGROUND ➔

PLEASE NOTE THAT THIS CHECKLIST WILL BE PUBLISHED ALONGSIDE YOUR PAPER

Corresponding Author Name: Makoto Higuchi, Masafumi Shimojo
Journal Submitted to: EMBO Journal
Manuscript Number: EMBOJ: EMBOJ-2021-107757R

USEFUL LINKS FOR COMPLETING THIS FORM

http://www.antibodypedia.com
http://1degreebio.org
http://www.equator-network.org/reporting-guidelines/improving-bioscience-research-repor
http://grants.nih.gov/grants/olaw/olaw.htm
http://www.mrc.ac.uk/Ourresearch/Ethicsresearchguidance/Useofanimals/index.htm
http://ClinicalTrials.gov
http://www.consort-statement.org
http://www.consort-statement.org/checklists/view/32-consort/66-title
http://www.equator-network.org/reporting-guidelines/reporting-recommendations-for-tumo
http://datadryad.org
http://figshare.com
http://www.ncbi.nlm.nih.gov/gap
http://www.ebi.ac.uk/ega
http://biomodels.net/
http://biomodels.net/miriam/
http://jjj.biochem.sun.ac.za
http://oba.od.nih.gov/biosecurity/biosecurity_documents.html
http://www.selectagents.gov/

Reporting Checklist For Life Sciences Articles (Rev. June 2017)

This checklist is used to ensure good reporting standards and to improve the reproducibility of published results. These guidelines are consistent with the Principles and Guidelines for Reporting Preclinical Research issued by the NIH in 2014. Please follow the journal's authorship guidelines in preparing your manuscript.

A- Figures
1. Data
The data shown in figures should satisfy the following conditions:

- the data were obtained and processed according to the field's best practice and are presented to reflect the results of the experiments in an accurate and unbiased manner.
- figure panels include only data points, measurements or observations that can be compared to each other in a scientifically meaningful way.
- graphs include clearly labeled error bars for independent experiments and sample sizes. Unless justified, error bars should not be shown for technical replicates.
- if n< 5, the individual data points from each experiment should be plotted and any statistical test employed should be justified
- Source Data should be included to report the data underlying graphs. Please follow the guidelines set out in the author ship guidelines on Data Presentation.

2. Captions

Each figure caption should contain the following information, for each panel where they are relevant:

- a specification of the experimental system investigated (eg cell line, species name).
- the assay(s) and method(s) used to carry out the reported observations and measurements
- an explicit mention of the biological and chemical entity(ies) that are being measured.
- an explicit mention of the biological and chemical entity(ies) that are altered/varied/perturbed in a controlled manner.

- the exact sample size (n) for each experimental group/condition, given as a number, not a range;
- a description of the sample collection allowing the reader to understand whether the samples represent technical or biological replicates (including how many animals, litters, cultures, etc.).
- a statement of how many times the experiment shown was independently replicated in the laboratory.
- definitions of statistical methods and measures:
  - common tests, such as t-test (please specify whether paired vs. unpaired), simple χ2 tests, Wilcoxon and Mann-Whitney tests, can be unambiguously identified by name only, but more complex techniques should be described in the methods section;
  - are tests one-sided or two-sided?
  - are there adjustments for multiple comparisons?
  - exact statistical test results, e.g., P values = x but not P values < x;
  - definition of 'center values' as median or average;
  - definition of error bars as s.d. or s.e.m.

Any descriptions too long for the figure legend should be included in the methods section and/or with the source data.

In the pink boxes below, please ensure that the answers to the following questions are reported in the manuscript itself. Every question should be answered. If the question is not relevant to your research, please write NA (non applicable). We encourage you to include a specific subsection in the methods section for statistics, reagents, animal models and human subjects.

B- Statistics and general methods

Please fill out these boxes ➔ (Do not worry if you cannot see all your text once you press return)

1.a. How was the sample size chosen to ensure adequate power to detect a pre-specified effect size?	Standard sample sizes were chosen according to previous studies.
1.b. For animal studies, include a statement about sample size estimate even if no statistical methods were used.	No statistical methods were used to estimate the sample size before the experiment.
2. Describe inclusion/exclusion criteria if samples or animals were excluded from the analysis. Were the criteria pre-established?	No samples or animals were excluded in this study.
3. Were any steps taken to minimize the effects of subjective bias when allocating animals/samples to treatment (e.g. randomization procedure)? If yes, please describe.	All animals/samples were randomly allocated to groups according to the order of ID numbers.
For animal studies, include a statement about randomization even if no randomization was used.	All animal experiments were performed according to the order of ID number. No further randomization procedure was performed in all experiments.
4.a. Were any steps taken to minimize the effects of subjective bias during group allocation or/and when assessing results (e.g. blinding of the investigator)? If yes please describe.	No blinding of the investigator was performed in this study.
4.b. For animal studies, include a statement about blinding even if no blinding was done	No blinding was performed in all animal experiments
5. For every figure, are statistical tests justified as appropriate?	Statistical tests were justified in every figure
Do the data meet the assumptions of the tests (e.g., normal distribution)? Describe any methods used to assess it.	Kolmogorov-Smirnov test was used to assess normality of the data.
Is there an estimate of variation within each group of data?	NA

Is the variance similar between the groups that are being statistically compared?	For comparison of groups with similar variance, data were analyzed by Unpaired Student's t-test or ANOVA with post-hoc test. Mann-Whitney U test was used for comparison of two-group shown in Fig 5E.

C- Reagents

6. To show that antibodies were profiled for use in the system under study (assay and species), provide a citation, catalog number and/or clone number, supplementary information or reference to an antibody validation profile. e.g., Antibodypedia (see link list at top right), 1DegreeBio (see link list at top right).	The following antibodies were commercially purchased and used in this study: chicken anti-GFP (AVES, GFP-1020); mouse anti-βActin (Sigma, A1978); mouse anti-FLAG, clone M2 (Sigma, F1804); mouse anti-HA (Covance, MMS-101R); rabbit anti-NeuN (Cell Signaling Tech, #12943S); rabbit anti-HA (Cell Signaling Tech, #3724); rabbit anti-cFos (Cell Signaling Tech, #2250); mouse anti-GFAP (Millipore, MAB360); rabbit anti-PDE10 (Protein Tech, 18078-1-AP); chicken anti-MAP2 (Abcam, AB5392).
7. Identify the source of cell lines and report if they were recently authenticated (e.g., by STR profiling) and tested for mycoplasma contamination.	human embryonic kidney 293T cells (ATCC, Cat No. CRL-3216)

* for all hyperlinks, please see the table at the top right of the document

D- Animal Models

8. Report species, strain, gender, age of animals and genetic modification status where applicable. Please detail housing and husbandry conditions and the source of animals.	We used postnatal day 0 or 2 month-old C57BL/6j mice (Japan SLC) for the genetic manipulation by Adeno Associate Virus (AAV) mediated gene delivery followed by two-photon microscopy, PET scan, histology, and metabolite analysis. We used both gender for the injection experiments with postnatal day 0 mice. We used male mice for all the other experiments unless otherwise stated. All mice were housed with littermates (2-5 mice depending on litter size) and maintained in a 12 h light/dark cycle with ad libitum access to standard diet and water. In Figure 3, A 3.4-year-old male common marmoset was used as model for tracing the neuronal circuit in primate brain. The animal was born at the National Institutes for Quantum and Radiological Science and Technology and was housed as one of a pair, with one family member of the same sex, in a cage.
9. For experiments involving live vertebrates, include a statement of compliance with ethical regulations and identify the committee(s) approving the experiments.	The animal studies here were maintained and handled following the National Research Council's Guide for the Care and Use of Laboratory Animals and our institutional guidelines. Protocols for the present animal experiments were approved by the Animal Ethics Committees of the National Institutes for Quantum and Radiological Science and Technology.
10. We recommend consulting the ARRIVE guidelines (see link list at top right) (PLoS Biol. 8(6), e1000412, 2010) to ensure that other relevant aspects of animal studies are adequately reported. See author guidelines, under 'Reporting Guidelines'. See also: NIH (see link list at top right) and MRC (see link list at top right) recommendations. Please confirm compliance.	Experiments were performed and reported in accordance with the ARRIVE (Animal Research: Reporting in Vivo Experiments) guidelines.

E- Human Subjects

11. Identify the committee(s) approving the study protocol.	NA
12. Include a statement confirming that informed consent was obtained from all subjects and that the experiments conformed to the principles set out in the WMA Declaration of Helsinki and the Department of Health and Human Services Belmont Report.	NA
13. For publication of patient photos, include a statement confirming that consent to publish was obtained.	NA
14. Report any restrictions on the availability (and/or on the use) of human data or samples.	NA
15. Report the clinical trial registration number (at ClinicalTrials.gov or equivalent), where applicable.	NA
16. For phase II and III randomized controlled trials, please refer to the CONSORT flow diagram (see link list at top right) and submit the CONSORT checklist (see link list at top right) with your submission. See author guidelines, under 'Reporting Guidelines'. Please confirm you have submitted this list.	NA
17. For tumor marker prognostic studies, we recommend that you follow the REMARK reporting guidelines (see link list at top right). See author guidelines, under 'Reporting Guidelines'. Please confirm you have followed these guidelines.	NA

F- Data Accessibility

18: Provide a "Data Availability" section at the end of the Materials & Methods, listing the accession codes for data generated in this study and deposited in a public database (e.g. RNA-Seq data: Gene Expression Omnibus GSE39462, Proteomics data: PRIDE PXD000208 etc.) Please refer to our author guidelines for 'Data Deposition'.<br><br>Data deposition in a public repository is mandatory for:<br>a. Protein, DNA and RNA sequences<br>b. Macromolecular structures<br>c. Crystallographic data for small molecules<br>d. Functional genomics data<br>e. Proteomics and molecular interactions	This study includes no data deposited in external repositories.
19. Deposition is strongly recommended for any datasets that are central and integral to the study; please consider the journal's data policy. If no structured public repository exists for a given data type, we encourage the provision of datasets in the manuscript as a Supplementary Document (see author guidelines under 'Expanded View' or in unstructured repositories such as Dryad (see link list at top right) or Figshare (see link list at top right).	NA
20. Access to human clinical and genomic datasets should be provided with as few restrictions as possible while respecting ethical obligations to the patients and relevant medical and legal issues. If practically possible and compatible with the individual consent agreement used in the study, such data should be deposited in one of the major public access-controlled repositories such as dbGAP (see link list at top right) or EGA (see link list at top right).	NA
21. Computational models that are central and integral to a study should be shared without restrictions and provided in a machine-readable form. The relevant accession numbers or links should be provided. When possible, standardized format (SBML, CellML) should be used instead of scripts (e.g. MATLAB). Authors are strongly encouraged to follow the MIRIAM guidelines (see link list at top right) and deposit their model in a public database such as Biomodels (see link list at top right) or JWS Online (see link list at top right). If computer source code is provided with the paper, it should be deposited in a public repository or included in supplementary information.	NA

G- Dual use research of concern

22. Could your study fall under dual use research restrictions? Please check biosecurity documents (see link list at top right) and list of select agents and toxins (APHIS/CDC) (see link list at top right). According to our biosecurity guidelines, provide a statement only if it could.	NA
